# Multiomics Mendelian randomization identifies serpin family G member 1 as a chronic obstructive pulmonary disease modulator

**DOI:** 10.1038/s41392-025-02547-7

**Published:** 2026-01-21

**Authors:** Erkang Yi, Jieda Cui, Hairong Wang, Fan Wu, Qiyang Hong, Qingyang Li, Chengshu Xie, Huahua Xu, Yu Liu, Xinru Ran, Xiaohui Wu, Qi Wan, Gaoying Tang, Leqing Zhu, Junling Pang, Yumin Zhou, Erping Long, Pixin Ran

**Affiliations:** 1 State Key Laboratory of Respiratory Disease, National Clinical Research Center for Respiratory Diseases, National Center for Respiratory Medicine, Guangzhou Institute of Respiratory Health, the First Affiliated Hospital of Guangzhou Medical University, Guangzhou, Guangdong China; 2 Guangzhou National Laboratory, Guangzhou, Guangdong China; 3 State Key Laboratory of Respiratory Health and Multimorbidity, Institute of Basic Medical Sciences, Chinese Academy of Medical Sciences and Peking Union Medical College, Beijing, China; 4 Department of Medical Oncology, The Second Affiliated Hospital of Guangzhou Medical University, Guangzhou, Guangdong China; 5 GMU-GIBH Joint School of Life Sciences, Guangzhou Medical University, Guangzhou, Guangdong China

**Keywords:** Respiratory tract diseases, Prognostic markers, Genome, Functional genomics

## Abstract

Chronic obstructive pulmonary disease (COPD), the third leading cause of death worldwide, lacks effective disease-modifying therapies, partly because of complex gene–environment interactions and extensive missing heritability. Here, we applied a multiomics Mendelian randomization (MR) framework—integrating proteome- and transcriptome-wide association analyses (pQTLs/eQTLs) with genome-wide association summary statistics, sensitivity analyses, and colocalization—to assign evidence levels to genes and prioritize those with higher causal likelihoods across diverse cohorts. We identified serpin family G member 1 (SERPING1) as a robust causal candidate, with consistent pQTL associations with COPD (β = –0.038 to –0.006) and with lung function measures, including FEV₁ (β = 0.008 to 0.015) and FEV₁/FVC% (β = 0.014 to 0.026). Longitudinal analyses in the UK Biobank (n = 46,369) and ECOPD cohort (n = 576) revealed that higher circulating SERPING1 protein levels were causally linked to slower FEV₁ decline during early follow-up (UKB: adjusted difference = –22.1 mL/year per standardized unit; ECOPD: –0.73 mL/year per ng/mL), accompanied by marked expression differences between European (higher) and Asian (lower) smokers and COPD patients. In a murine model exposed to cigarette smoke, AAV-mediated SERPING1 overexpression improved lung function, reduced alveolar destruction, and upregulated the expression of fibroblast elastic fiber–related genes. Collectively, these findings identify SERPING1 as a complement pathway regulator that may function both as a short-term biomarker of lung function decline and as a population specific, disease-modifying therapeutic target for COPD.

## Introduction

Chronic obstructive pulmonary disease (COPD), a leading cause of mortality and disability worldwide, is characterized by persistent airflow limitation driven by heterogeneous pathological processes, including chronic bronchitis, emphysema, and small airway remodeling.^1^ Despite its continued status as the third leading cause of death worldwide, COPD remains devoid of disease-modifying pharmacotherapies.^2^ Current therapeutic paradigms are confined to symptomatic management and reduction of acute exacerbations via the use of bronchodilators and inhaled corticosteroids,^1^ with no interventions currently capable of altering the progressive trajectory of the disease.^3^ COPD is typically diagnosed at advanced stages (GOLD stages 3 and 4) and is frequently associated with poor prognosis. However, a substantial number of individuals with mild COPD (GOLD stages 1 and 2) exist; due to the absence of overt respiratory symptoms, diagnosis is often delayed in this population, resulting in missed opportunities for optimal treatment and possible progression to more severe stages of the disease. This grim situation highlights the urgent need to elucidate the molecular mechanisms underlying COPD and to identify potential predictive and therapeutic targets.^4^ The complexity of COPD arises from its multifactorial etiology, where oxidative stress, protease‒antiprotease imbalance, and chronic inflammation interact with genetic susceptibility and environmental exposure, such as smoking and air pollution.^5^ However, the precise causal pathways linking these factors to disease progression remain poorly defined, hindering the development of targeted interventions.

COPD pathogenesis is shaped by intricate gene‒environment interactions; however, the genetic architecture of the disease remains incompletely understood.^6^ Although genome-wide association studies (GWASs) have identified loci such as *CHRNA3/5* and *FAM13A* as susceptibility loci associated with COPD pathogenesis,^7,8^ they explain less than 10% of heritability, leaving substantial “missing heritability” attributable to rare variants, regulatory elements, or gene‒environment interplay.^6^ Mendelian randomization (MR), a causal inference method that uses genetic variants as instrumental variables, has emerged as a powerful tool for prioritizing therapeutic targets by reducing confounding factors and avoiding reverse causation.^9^ To address these inherent limitations of traditional observational studies, MR leverages the random allocation of genetic variants at conception—mimicking a natural randomized trial—to provide stronger evidence for causal inference. Recent advances in proteome- and transcriptome-wide MR (pQTL-/eQTL-MR) have further enabled the integration of genetic variants with protein or RNA expression data, thereby offering deeper insights into the underlying molecular mechanisms of complex diseases.^10^ For example, proteome-wide MR (pQTL-MR) has successfully identified drug targets, such as *IL-6R*, for sepsis and sepsis-related mortality,^11^ demonstrating its potential to bridge genetic associations with therapeutic discovery. However, the application of MR-based screening of therapeutic targets remains limited.^12^ Existing studies are often constrained by small sample sizes, overlapping cohorts, or a lack of validation, resulting in inconsistent findings, weak evidence of causal relationships, and potential bias.

Despite substantial advances in elucidating the genetic basis of COPD in recent years, critical and long-standing gaps remain in translating these genetic association findings into interpretable molecular mechanisms and clinically actionable biomarkers. Most existing GWASs lack sufficient population diversity, with study cohorts redominantly composed of individuals of European ancestry, thereby limiting the generalizability and applicability of the results across global populations. In addition, these studies often fail to adequately adjust for key environmental covariates closely linked to COPD pathogenesis—such as cumulative smoking exposure, long-term occupational inhalant stimuli, and ambient air pollution levels^13^—which may introduce confounding and bias in the estimation of genetic effects. Moreover, COPD shares extensive genetic risk with several common comorbid conditions, including lung cancer and atherosclerotic cardiovascular disease, and this highly overlapping genetic architecture substantially increases the difficulty of distinguishing COPD-specific etiological signals from comorbidity-related signals.^14^ Collectively, these limitations point to the need for large-scale, multi-ancestry multiomics research frameworks that integrate genomic, transcriptomic, and proteomic data and systematically validate key molecular findings in experimental models, in order to establish robust causal chains and clarify their biological significance.

To address the methodological gaps highlighted above—particularly those related to population heterogeneity and limitations in causal inference, we established and implemented a systematic, integrative multiomics strategy. Specifically, we incorporated: (1) harmonized GWAS meta-analyses conducted across discovery, validation, and replication cohorts from international consortia to increase statistical power and enhance the robustness and reproducibility of findings across diverse populations; (2) multidimensional molecular expression and protein quantitative trait loci (eQTL/pQTL) resources spanning multiple ancestries and tissue types to finely characterize the regulatory effects of genetic variants on downstream transcriptomic and proteomic phenotypes; and (3) pQTL-/eQTL-based Mendelian randomization analyses integrated with multiple sensitivity frameworks for causal evidence grading, allowing us to distinguish correlation from causation and prioritize candidate pathways. For the prioritized targets generated through this pipeline, we conducted cross-cohort replication analyses, single-cell transcriptomic profiling of specimens derived from COPD patients to resolve fine-grained expression patterns within diseased cell populations, and mechanistically oriented functional assays—including in vitro cellular models and in vivo murine systems—to perform multilayered, closed-loop validation. This integrated workflow aims to redefine the pathogenic landscape of COPD and identify predictive and therapeutic targets with potential for clinical translation.

## Results

### Multiomics MR identifies causal targets for COPD

We performed summary-data-based Mendelian randomization (SMR) analyses via two eQTL and eight pQTL datasets (Supplementary Data 1, Supplementary Fig. 1) across COPD, forced expiratory volume in 1s (FEV₁), FEV₁ percent predicted (FEV₁% predicted), and FEV1/forced vital capacity (FEV₁/FVC) GWAS cohorts (Supplementary Data 2, Supplementary Fig. 2a). Significant positive and negative gene–trait associations were observed for all COPD phenotypes (Supplementary Fig. 2b–e, Supplementary Data 3–10). The eQTLGen results revealed partially inconsistent gene directionality across cohorts (Supplementary Fig. 3a), reflecting COPD heterogeneity and underscoring the need for independent validation.

**Fig. 1:**
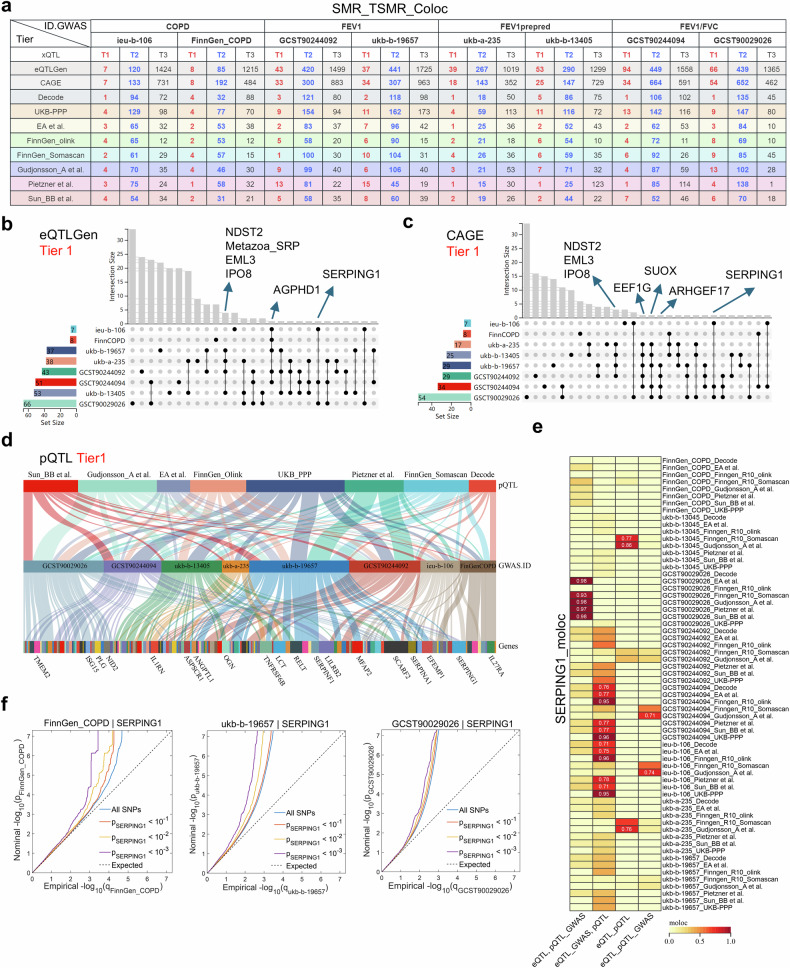
Multiomics MR identifies SERPING1 as a causal target for COPD. **a** This table quantifies the evidence-tiered gene counts derived from integrated analyses of multiple independent datasets. SMR, TSMR, and colocalization analyses were performed on COPD (ieu-b-106, and FinnGen_COPD), FEV₁ (GCST90244092, and ukb-b-19657), FEV₁% predicted (ukb-a-235, and ukb-b-13405), and FEV₁/FVC% (GCST90244094, and GCST90029026) GWAS summary statistics via eQTL data from Cap Analysis of Gene Expression (CAGE) and eQTLGen Consortium, plus pQTL data from Decode, UKB-PPP, EA et al., FinnGen_Olink, FinnGen_Somascan, Gudjonsson_A et al., Pietzner et al., and Sun_BB et al. Genes were stratified into three evidence tiers: Tier 1 (red), concordant support from SMR, TSMR, and colocalization; Tier 2 (blue), significant in *any* of the *two* methods; and Tier 3 (black), significant *only* in SMR. Upset plots displaying intersections of cis-eQTL-derived Tier 1 genes identified in the eQTLGen consortium (**b**) and CAGE project (**c**) datasets. **d** Sankey diagram visualizing shared Tier 1 pQTL genes identified through integrated analysis of multiple pQTL datasets against distinct GWAS datasets. **e**. Heatmap illustrating the moloc colocalization results for *SERPING1* eQTLs, pQTLs, and GWASs of COPD/pulmonary function traits, where darker color intensity indicates stronger colocalization; grid cells with numerical labels signify significant colocalization events (*PPH4* > *0.7*). **f** Conditional Q‒Q plots for polygenic overlap analyses: (left) FinnGen_COPD conditioned on *SERPING1* pQTL, (middle) ukb-19657 (FEV₁) conditioned on *SERPING1* pQTL, (right) GCST90029026 (FEV₁/FVC) conditioned on *SERPING1* pQTL; x-axis: Empirical –log₁₀(*p*); y-axis: Nominal -log₁₀(*p*) after genomic control adjustment; solid black line: all SNPs; dashed gray lines: conditional SNPs with *SERPING1* pQTL significance thresholds (*p* < 0.1*, p* < 0.01, and *p* < 0.001); dotted gray line: Expected null distribution

Genes from SMR analyses were subjected to two-sample MR [TSMR; Inverse Variance Weighted or Wald ratio] and colocalization (*PPH4* > 0.7) (Supplementary Data 11–18). We categorized genes into three evidence tiers: Tier 1 (supported by both the TSMR and coacalization), Tier 2 (supported by either), and Tier 3 (supported by neither) (Fig. 1a). The Tier 1 eQTL genes included *NDST2*, *EML3*, *IPO8*, and *SERPING1*, whereas the Tier 1 pQTL genes included SERPING1, EFEMP1, SERPINA1, SCARF2, MFAP2, LILRB2, LCT, and IL1RN (Fig. 1b–d). Functional enrichment indicated convergence in the differentiation, adhesion, extracellular matrix, and cytokine pathways (Supplementary Fig. 3b–d).

Seven genes (*SERPING1*, *RGMB*, *DNAJB12*, *C1orf198*, *DNAJB4*, *IRF3*, *and ASPSCR1*) achieved Tier 1 status in both eQTL and pQTL analyses (Supplementary Fig. 3c). mQTL analysis excluded *RGMB* and *IRF3* because of a lack of exposure instruments. Strong evidence suggests that *SERPING1* methylation is associated with COPD and FEV₁/FVC; *ASPSCR1*, with FEV₁% predicted and FEV₁; and *DNAJB4*, with FEV₁ and FEV₁/FVC, through SMR, TSMR, and colocalization analyses (Supplementary Data 19). SER*PING1* was prioritized as the core gene because of its recurrent Tier 1 status and its consistent association with COPD and lung function.

Divergent causal directions emerged: *SERPING1* eQTL was positively associated with COPD risk but negatively associated with lung function. In contrast, pQTLs for *SERPING1* are negatively associated with COPD and positively associated with lung function; these associations are unaffected by horizontal pleiotropy or heterogeneity (Supplementary Fig. 4a, b). SMR and colocalization analyses indicated a high degree of overlap among eQTLs, pQTLs, and the trait (Supplementary Fig. 5a, b, Supplementary Fig. 6). Moloc colocalization confirmed robust *SERPING1* associations (*H3* + *H4* + *H5* > *0.7* for COPD and FEV₁/FVC) (Fig. 1e, Supplementary Data 20), which were corroborated by Hyprcoloc analysis (*posterior probability* > *0.7*; Supplementary Fig. 7, Supplementary Data 21). Reverse MR analysis revealed no significant associations between COPD, lung function, and *SERPING1* expression (Supplementary Data 22).

Linkage disequilibrium score regression (LDSC) revealed a significant genetic correlation between the *SERPING1* pQTL and FEV₁ (Supplementary Fig. 8a). The pleiotropy false discovery rate (PleioFDR) identified shared loci for *SERPING1* and COPD risk/lung function (Fig. 1f), whereas no associations emerged when conditioning for COPD risk/lung function, with *SERPING1* as the primary trait (Supplementary Figs. 8–11). These findings support the causal influence of SERPING1 on COPD risk/lung function. PleioFDR further highlighted colocalized *single nucleotide polymorphisms (SNPs) (conjunctive false discovery rate [conjFDR]<0.01*) on chromosome 11 (Supplementary Figs. 8–11), validating the existence of cis-regulatory mechanisms. Overall, the *SERPING1* pQTL-mediated genetic variants modulated COPD risk and lung function.

### Clinical validation of *SERPING1* as a biomarker of COPD progression in the UKB cohort

**Fig. 2:**
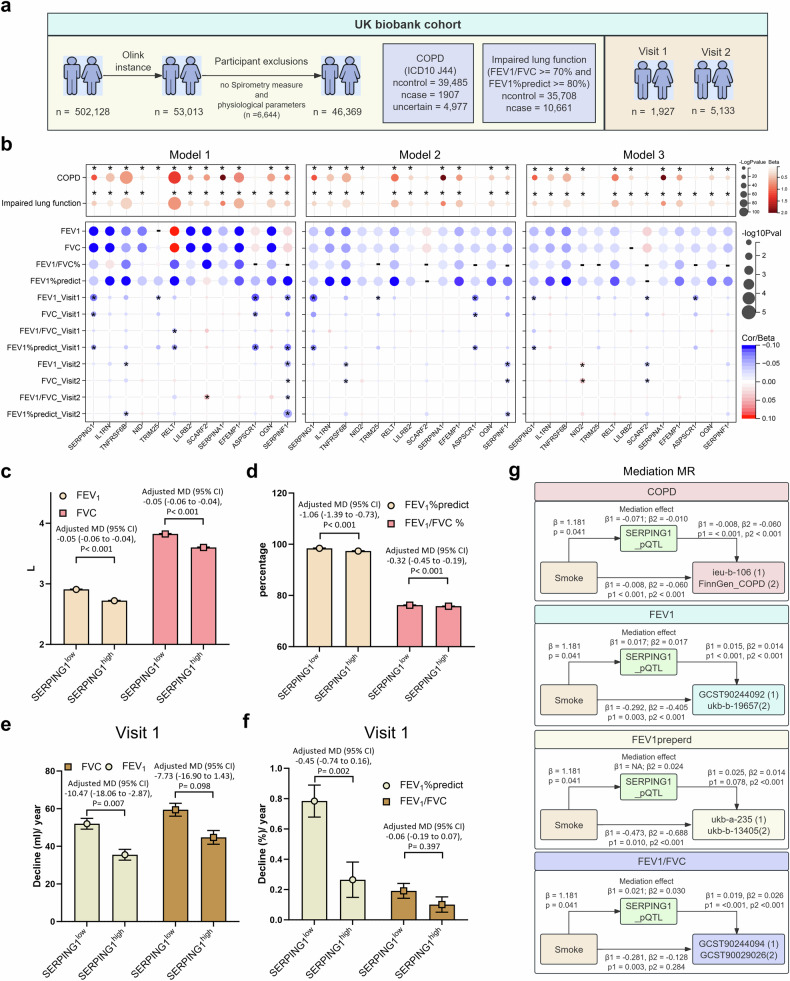
Clinical validation of SERPING1 as a biomarker of COPD progression in the UKB cohort. **a** UKB cohort selection flowchart: Initially, 502,128 Olink instances; excluded *n* = 6644 lacking spirometry; retained *n* = 46,369 stratified as COPD (ICD-10: J44; cases = 1907; controls = 39,485), impaired lung function (FEV₁/FVC ≥ 70% and FEV₁% pred ≥ 80%; cases = 10,661; controls = 35,708), and uncertain (*n* = 4977); longitudinal cohorts: Visit 1 (*n* = 1927), Visit 2 (*n* = 5133). **b** Dot plot of Tier 1 pQTL gene associations (SERPING1, IL1RN, TNFRSF6B, NID, TRIM25, RELT, LILRB2, SCARF2, SERPINGA1, EFEMP1, ASPSCR1, OGN, and SERPINF1) with COPD status and impaired lung function in the UKB cohort, including baseline parameters (FEV₁, FVC, FEV₁/FVC ratio, and FEV₁% predicted) and annual decline rates at Visits 1 and 2 (FEV₁, FVC, FEV₁/FVC ratio, and FEV₁% predicted), analyzed by univariate regression (Model 1: unadjusted) and multivariable regression (Model 2: adjusted for age, sex, body mass index (BMI), and smoking status; Model 3: adjusted for age, sex, BMI, smoking status, alcohol intake frequency, and income). Red circles indicate positive associations, blue circles indicate negative associations, and the circle size is proportional to −log₁₀(*p* value) (larger circles denote stronger statistical significance). Bar charts displaying adjusted differences in (**c**) baseline FEV₁ and FVC and (**d**) FEV₁% predicted and FEV₁/FVC ratios between the high- and low-SERPING1 expression groups in the UKB cohort. The groups were stratified by median serum SERPING1 levels (Olink assay). Bar charts displaying adjusted annual decline rates in FEV₁ and FVC (**e**) and the FEV₁% predicted and FEV₁/FVC ratios (**f**) between the high- and low-SERPING1 expression groups at Visit 1 in the UKB cohort. The groups were stratified by median serum SERPING1 levels (Olink assay). **g** Schematic diagram of the MR mediation framework displaying causal pathways from smoking (exposure) to COPD and pulmonary function measures (FEV₁, FEV₁% predicted, and FEV₁/FVC ratio) through *SERPING1* pQTL (mediator), with four colored sections representing COPD (pink), FEV₁ (blue), FEV₁% predicted (green), and the FEV₁/FVC ratio (purple). Each section displays bidirectional arrows connecting the exposure, mediator, and outcome elements annotated with β coefficients and *p* value labels, with supplementary data sources labeled (1) and (2), as displayed. Analyses were adjusted for age, sex, BMI, self-reported race, smoking status (never, former, or current), alcohol intake frequency, and household income (c-f). The error bars represent the and SEM (c-f)

To validate the core risk gene *SERPING1* identified through MR analyses, we included 46,369 individuals from the UK Biobank (UKB) cohort with both Olink proteomic data and spirometry measurements. Among them, 1,927 and 5,133 participants completed follow-up visits 1 and 2, respectively (Fig. 2a, Supplementary Data 23). We further analyzed the protein expression levels of 13 biomarkers (SERPING1, IL1RN, TNFRSF6B, NID2, TRIM25, RELT, LILRB2, SCARF2, SERPINA1, EFEMP1, ASPSCR1, OGN, and SERPINF1) that were recurrently observed as Tier 1 proteins in prior pQTL-based MR analyses. Three regression models were employed to assess associations with COPD risk, lung function impairment, and baseline spirometric indices: (1) univariable/correlation analysis; (2) multivariable analysis adjusted for physiological confounders; and (3) multivariable analysis adjusted for both physiological and subjective confounders. Most proteins exhibited significant associations with COPD, impaired lung function, and baseline spirometry (FEV₁, FEV₁% predicted, FVC, and FEV₁/FVC), with robustness sustained after confounder adjustment—these findings are consistent with our pQTL-MR results (Fig. 2b, Supplementary Data 24, 25). Exceptions included *TRIM25*, *LILRB2*, *ASPSCR1*, and *SCARF2*, for which the associations were nonsignificant.

Longitudinal analyses revealed that *SERPING1*, *SCARF2*, and *ASPSCR1* levels were inversely associated with the annual rate of FEV₁ decline at visit 1. Only *SERPING1* exhibited a significant inverse association with the predicted decrease in FEV₁%, which persisted after adjusting for confounders (Fig. 2b, Supplementary Data 25). Notably, the results at visit 2 differed; *SERPINF1* was inversely correlated with lung function decline rates, whereas confounder-adjusted *SCARF2* exhibited inverse associations with FEV₁ and FVC decline rates, and *NID2* demonstrated positive associations (Fig. 2b, Supplementary Data 25). These discrepancies may have arisen from protein-level instability and extended follow-up intervals.

Analysis of *SERPING1* further revealed elevated expression in patients with COPD and individuals with impaired lung function (Supplementary Fig. 12a, b). Grouped regression (stratifying participants into high and low SERPING1 expression subgroups) corroborated the linear regression results, confirming associations independent of confounders (Supplementary Fig. 3c–f, Supplementary Data 26). UKB data indicated that although *SERPING1* expression was increased in COPD patients, it was inversely correlated with the decline rates of most lung function indices, findings aligned with the pQTL-MR results for *SERPING1*.

Smoking is the most common pathogenic factor for COPD. We used the TSMR to analyze the causal relationships between smoking and *SERPING1* expression, COPD risk, and changes in lung function. We found that smoking was positively correlated with elevated *SERPING1* levels and an increased risk of COPD. It was negatively correlated with pulmonary function indicators (Supplementary Fig. 12c, Supplementary Data 27). The Mediation MR method further verified the mediating effect of the SERPING*1* pQTL on smoking-mediated COPD risk and most changes in FEV₁ (Fig. 2g, Supplementary Data 28), indicating that SERPING1 plays a protective role.

### Divergent *SERPING1* expression in European versus Asian populations

**Fig. 3:**
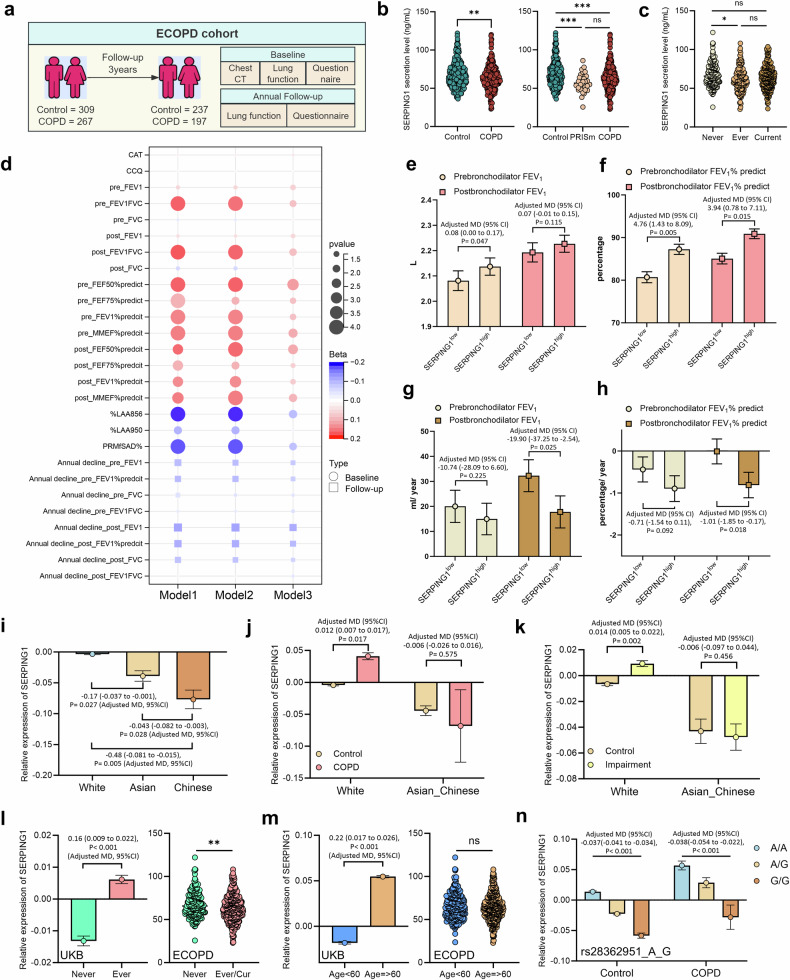
ECOPD Cohort Reveals Differential SERPING1 Expression in European and Asian Populations. **a** Flowchart of the ECOPD cohort plasma sample collection: Baseline peripheral plasma samples (*n* = 576) for *SERPING1* quantification and longitudinal subcohort completing 3-year follow-up (*n* = 434). **b** Serum *SERPING1* expression distribution in controls versus patients with COPD. **c** *SERPING1* distribution across the control, PRISM, and COPD groups. **d** Dot plot of associations between *SERPING1* and lung function metrics in the ECOPD cohort: baseline parameters (pre- and postbronchodilator FEV₁, FVC, FEV₁/FVC ratio, and FEV₁% predicted) and annual decline rates at visit (pre/postbronchodilator FEV₁, FVC, FEV₁/FVC ratio, and FEV₁% predicted), analyzed by correlation analysis (Model 1), univariate regression (Model 2: unadjusted), and multivariate regression (Model 3: adjusted for age, sex, BMI, smoking status, and smoking index). Red circles indicate positive associations, blue circles indicate negative associations, and the circle size is proportional to −log₁₀ (*p* value). Bar charts displaying adjusted differences in baseline pre/postbronchodilator FEV₁ (**e**) and FEV₁% predicted (**f**) and the annual decline in pre/postbronchodilator FEV₁ (**g**) and FEV₁% predicted (**h**) between the high- and low-*SERPING1* expression groups in the ECOPD cohort. The groups were stratified by median serum *SERPING1* levels (via ELISA) with analyses adjusted for age, sex, BMI, smoking status, and smoking index. **i** Bar chart depicting adjusted differences in serum *SERPING1* levels between White, Asian, and Chinese ethnic groups within the UKB cohort. Bar charts displaying adjusted differences in serum *SERPING1* levels between the control and COPD groups (**j**) and between the control and impaired lung function groups (**k**), stratified by the White and Asian_Chinese ethnic groups in the UKB cohort. **l** Adjusted differences in serum *SERPING1* levels between never smokers and ever smokers in the UKB cohort (left) and differences in serum *SERPING1* levels between never smokers and Ever/Current smokers in the ECOPD cohort (right). **m** Adjusted differences in serum *SERPING1* levels between ages <60 years and ≥60 years in the UKB cohort (left) and differences in serum *SERPING1* levels between ages <60 years and ≥60 years in the ECOPD cohort (right). **n** Bar chart displaying adjusted differences in serum *SERPING1* expression levels among the rs28362951 genotype groups (wild-type, heterozygous, and homozygous variants) in the control and COPD populations within the UKB cohort. Analyses were adjusted for age, sex, BMI, race, smoking status (never, former, or current), alcohol intake frequency, and household income (i-n). The error bars represent the standard deviations (SDs; b, c) and Standard Error of the Mean (SEM, e-n)

To further validate the UKB findings, we utilized the Early COPD cohort as an external validation set and randomly enrolled 576 participants (controls = 309, COPD = 267) for peripheral plasma *SERPING1* protein quantification, with 434 participants completing the 3-year follow-up period (Fig. 3a, Supplementary Data 29). In contrast to the UKB results, *SERPING1* expression was significantly reduced in COPD patients within the ECOPD cohort, a pattern also observed in the preserved ratio impaired spirometry (PRISm) cohort (Fig. 3b) and exclusively among ever-smokers and current smokers (Fig. 3c). Three regression models (correlation analysis, univariable regression, and multivariable regression adjusted for confounders) revealed that *SERPING1* levels were positively correlated with baseline pre- and postbronchodilator FEV₁/FVC, FEV₁% predicted, and small airway function indices (maximal mid-expiratory flow [MMEF]% predicted, forced expiratory flow at 50% of FVC [FEF₅₀]% predicted, FEF₇₅% predicted) but inversely correlated with CT-based Percentage of Lung Area with Attenuation less than -856 Hounsfield Units (%LAA-856) and parametric response mapping-functional small airway disease (PRMfSAD)%; however, no associations with COPD assessment test (CAT) or Clinical COPD Questionnaire (CCQ) scores (Fig. 3d, Supplementary Fig. 12d, Supplementary Data 30).

Longitudinal analyses revealed inverse correlations between *SERPING1* and the annual decline rates of pre- and postbronchodilator FEV₁ and FEV₁% predicted (Fig. 3d), which was in line with UKB trends. Grouped regression (stratifying participants by high versus low *SERPING1* expression) demonstrated directional effects consistent with continuous variable analyses, although statistical significance was achieved only for prebronchodilator FEV₁, pre- and postbronchodilator FEV₁% predicted, subsets of small airway indices, and PRMfSAD% (Fig. 3e, f, Supplementary Fig. 12e, f; Supplementary Data 31). After confounder adjustment, postbronchodilator FEV₁ and FEV₁% were significantly associated with decline rates (Fig. 3g, h; Supplementary Data 31).

Both cohorts demonstrated a slower decline in lung function in patients with high *SERPING1* expression, supporting its potential protective role. The direction of expression changes in patients with COPD differed between UKB (upregulation) and ECOPD (downregulation), possibly because of UKB’s predominantly European composition versus ECOPD’s exclusively Chinese cohort. To test this hypothesis, we analyzed UKB subgroups and found that *SERPING1* expression progressively decreased from Europeans to Asians to Chinese individuals (Fig. 3i); compared with that in controls, SERPING1 expression was elevated in European COPD/impaired lung function groups but reduced in Asian counterparts (Fig. 3j, k). Compared with Europeans, Asians presented significantly lower expression levels across all subgroups (Supplementary Fig. 12g, h), suggesting that ethnicity-dependent expression dynamics may mediate prognostic heterogeneity. TSMR and SMR analyses revealed no causal links between *SERPING1* and IPF or asthma (Supplementary Fig. 13a, Supplementary Data 32), implying that *SERPING1* may be a COPD-specific biomarker.

The highly colocalized SNP *rs28362951* showed a greater mutation frequency in Europeans than in East Asians and Africans (gnomAD; Supplementary Fig. 13b). SERPING1 expression showed a stepwise decrease from the wild-type genotype to the heterozygous and homozygous mutant genotypes (Supplementary Fig. 13c). The heterozygous carriers presented elevated baseline FEV₁/FVC%, whereas the homozygous mutants presented accelerated lung function decline (Supplementary Fig. 13d). The lowest *SERPING1* expression in the latter suggests that this SNP may drive a decline in risk via the regulation of *SERPING1*. However, this variant alone cannot explain the divergent SERPING1 expression patterns across different populations.

### *SERPING1* participates in COPD pathogenesis via the complement cascade

*SERPING1* has been reported to be a specific inhibitor of the complement system, selectively targeting *the complement C1s/C1r subcomponent* (*C1S/C1R*) to regulate downstream complement activation (Supplementary Fig. 14a). To investigate this relationship, we analyzed key complement proteins (C1QA, C1S, C1R, C2, and C3) and their associations with COPD risk and lung function. The results indicated that *SERPING1* expression was inversely correlated with C1QA but positively correlated with other complement components. All complement components, except C1QA, demonstrated positive correlations with COPD risk and impaired lung function, whereas negative correlations were observed with the baseline lung function indices (Supplementary Fig. 14b, c; Supplementary Data 33, 34). Although C1QA was negatively correlated with the rate of decrease in lung function, C2 and C3 were positively correlated, but the correlations were not statistically significant. However, these proteins are unsuitable predictors of lung function decline.

Stratified regression revealed lower *C1QA* expression in Asian populations with normal lung function than in European populations, along with higher *C2* and *C3* expression. This pattern persists in individuals with impaired lung function. Among patients with COPD, only *C3* exhibited significant ancestry-dependent differences (Supplementary Fig. 14d–g, Supplementary Data 35). Two-sample MR analyses demonstrated that *C1QA* was positively correlated with lung function indices, whereas *C2* was negatively correlated with UKB trends (Supplementary Fig. 15a). A multivariable MR incorporating *C1QA, C1S, C1R, C2, C3*, and *SERPING1* indicated that adjusting for complement factors attenuated the effects of SERPING1 on COPD risk and lung function. After adjustment, *SERPING1* lost statistical significance for COPD risk and several lung function metrics (Supplementary Fig. 15b, Supplementary Data 36), suggesting that its influence on COPD pathogenesis and lung function trajectories is partially mediated by the complement system.

We further validated these findings via lung tissue RNA sequencing and peripheral blood transcriptomics in two independent COPD cohorts. *SERPING1* expression was significantly upregulated in both the lung tissue and peripheral blood of patients with COPD (Supplementary Fig. 16a), demonstrating negative correlations with lung function and positive correlations with emphysema indices (Supplementary Fig. 16d–d). Stratifying patients by high versus low *SERPING1* expression via GSEA revealed that high *SERPING1* expression (across both tissue and blood) was significantly enriched in inflammatory effector pathways, metabolic pathways, complement cascades, and interferon signaling (Supplementary Fig. 16e). These results implicate *SERPING1* in modulating complement-driven inflammatory responses during COPD progression.

### *SERPING1* dysregulation triggers fibroblast-driven lung inflammation

**Fig. 4:**
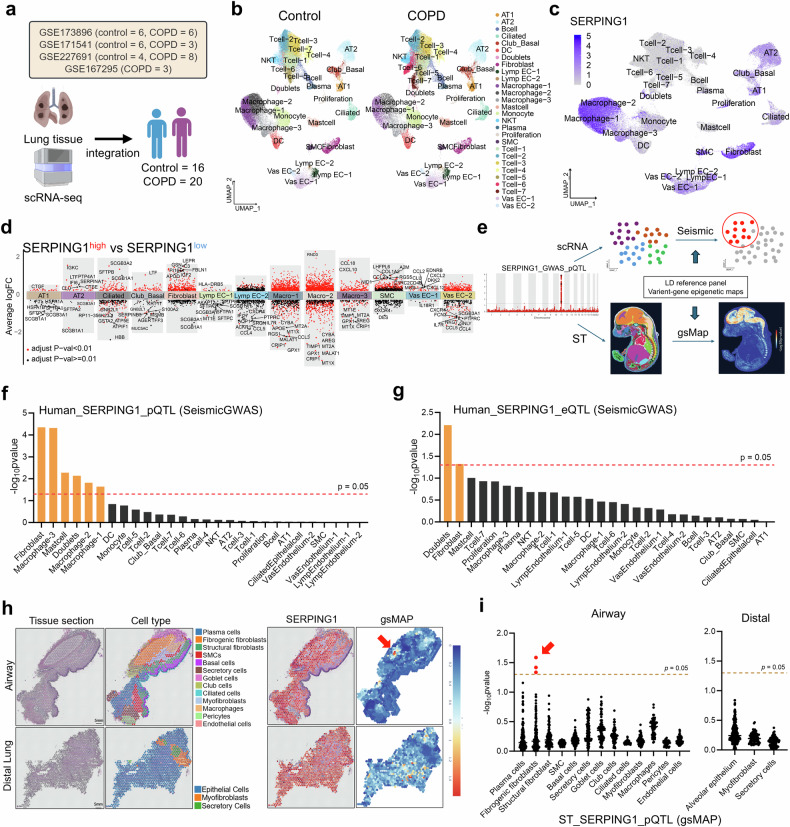
Multiomics profiling identifies effector cell populations targeted by SERPING1. **a** Integrated scRNA-seq analysis of lung tissues from patients with COPD and controls across datasets GSE173896, GSE171541, GSE227691, and GSE167295 (16 control and 20 COPD samples). UMAP visualization of integrated cell clusters in controls versus patients with COPD (**b**) and the distribution of *SERPING1* expression across cell types (**c**). **d** Multigroup volcano plots displaying DEGs between *SERPING1*^*high*^ and *SERPING1*^*low*^ cells across subpopulations: black points (adjusted *p* ≥ 0.05) and red points (adjusted *p* < 0.05). **e** Multiomic integration frameworks: SeismicGWAS for *SERPING1* eQTL/pQTL and single-cell RNA-seq data; gsMAP for *SERPING1* eQTL/pQTL and spatial transcriptomics. Integration of *SERPING1* pQTLs from Decode (**f**) and eQTLs from eQTLGen (**g**) with human lung tissue scRNA-seq profiles via the seismic GWAS framework. The red dashed line indicates the nominal significance threshold (*p* = 0.05), and the orange bars highlight cell subpopulations with significant seismic GWAS associations. Spatial transcriptomics of human airway/distal lung tissues from the STOmics DB: H&E-stained sections with capture spots color-coded by annotated cell type and *SERPING1* expression overlaid (**h**) and gsMAP-based multiomic integration of *SERPING1* pQTL data (deCODE genetics) with spatial transcriptomics (**i**); the color gradient indicates −log₁₀ (*p* value) significance levels per vertical scale bar (blue: low; red: high)

To elucidate the functional role of SERPING1 in COPD progression, we integrated single-cell RNA sequencing (scRNA-seq) data from four independent datasets, including 16 controls and 20 COPD patients (Fig. 4a). After reclustering and cell annotation (Fig. 4b, Supplementary Fig. 17a, b), SERPING1 was predominantly expressed in pulmonary fibroblasts, smooth muscle cells (SMCs), macrophages, and subsets of endothelial and club/basal cells (Fig. 4c, Supplementary 17c). Differential gene expression analysis between *SERPING1*^*high*^ and *SERPING1*^*low*^ cells (Fig. 4d) revealed marked downregulation in fibroblasts, SMCs, alveolar type 2 cells (AT2), macrophages, and certain endothelial subsets but upregulation in club/basal cells (Supplementary Fig. 17d, Supplementary Data 37). Integrative DEG analysis linked SERPING1 to cell adhesion, inflammatory effector responses, oxidative stress, and extracellular matrix (ECM) remodeling, particularly within interstitial cells and macrophages (Supplementary Fig. 17e, f).

To determine the cellular targets of SERPING1-associated variants, Single-cell Expression Integration System for Mapping genetically Implicated Cell types (SeismicGWAS) analysis^13^ (Fig. 4e) revealed significant pQTL correlations with pulmonary fibroblasts, macrophages, mast cells, and doublets, whereas eQTLs were restricted to fibroblasts and doublets (Fig. 4f, g). Spatial transcriptomics (ST) demonstrated widespread SERPING1 expression across airways and alveoli, whereas genetically informed spatial mapping (gsMAP)^14,15^ localized pQTL signals specifically to airway fibroblasts (Fig. 4h, i), with minimal eQTL associations (Supplementary Fig. 18a–c). Murine lung scRNA-seq data (GSE168299)^15^ confirmed SERPING1 expression in stromal cells, and SeismicGWAS indicated pQTL enrichment in macrophages and fibroblasts (Supplementary Fig. 18d–g). Mouse spatial gsMAP further linked both pQTLs and eQTLs to adventitial fibroblasts (Supplementary Fig. 19a–c), implicating fibroblasts as the main cellular hub for SERPING1 activity, with secondary roles in macrophages.

In human scRNA-seq, COPD-derived fibroblasts and macrophages with low SERPING1 expression presented elevated IL-6 and CXCL8 levels, unlike those in normal controls (Supplementary Fig. 20), suggesting disease-specific anti-inflammatory effects. The complement factors C1QA, C1S, C1R, and *C3* were localized mainly to macrophages and fibroblasts (Supplementary Fig. 21a–c). COPD lungs presented decreased C1QA but elevated C1S, C1R, and C3 (Supplementary Fig. 21b, Supplementary Data S38). CellChat analysis confirmed enhanced fibroblast–macrophage complement signaling (Supplementary Fig. 21d, e), supporting *SERPING1*-mediated regulation of complement hyperactivity.

Fibroblast subclustering revealed five subsets (Supplementary Fig. 21f, Supplementary Data S39), with the numbers of matrix fibroblasts and mesenchymal stem cells (MSCs) reduced in COPD patients (Supplementary Fig. 21g). Pseudotime analysis mapped transitions from progenitor (State 1) to mesenchymal (States 2–3) and inflammatory (States 4–7) states (Supplementary Fig. 22a, b). In State 1, ECM organization and elastin assembly were enriched, whereas inflammatory marker expression peaked in State 7, where *SERPING1* expression was lowest (Supplementary Fig. 23a). Notably, *SERPING1*^*high*^ cells were most abundant in State 1 (Supplementary Fig. 23b), implicating *SERPING1* in mesenchymal stem cell function and its ability to suppress inflammatory fibroblasts.

### SERPING1 modulates inflammatory effects in pulmonary fibroblasts in vitro

**Fig. 5:**
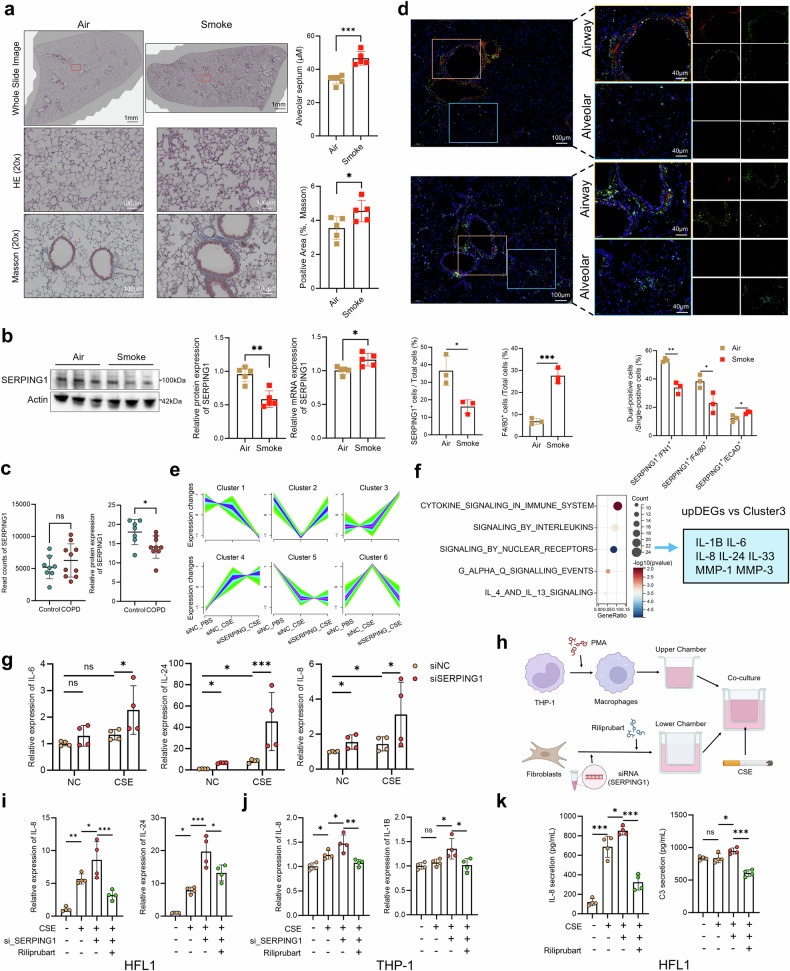
SERPING1 modulates inflammatory effects in pulmonary fibroblasts in vitro. **a** Histopathological alterations in lung tissues from CS-exposed (6 months) versus air-exposed mice, as shown by H&E and Masson’s trichrome staining (*n* = 5). **b** Comparison of *SERPING1* expression at the RNA and protein levels in the lung tissues of CS-exposed (6 months) and air-exposed mice (*n* = 5 per group). **c** Expression of *SERPING*1 at both the mRNA and protein levels in human lung tissue. **d** Multiplex immunofluorescence showing the expression and colocalization of Serping1 (red), Fn1 (green), Ecad (yellow), and F4/80 (light blue) in lung tissue sections from air-exposed and CS-exposed (CSE) mice. Quantification of Serping1⁺, F4/80⁺, Serping1⁺/Fn1⁺, Serping1⁺/F4/80⁺, and Serping1⁺/Ecad⁺ double-positive cells relative to the total number of cells (*n* = 3 per group). Nuclei were counterstained with DAPI (blue). **e** Mfuzz analysis of transcriptome sequencing data from the *siNC* + *PBS*, *siNC* + *CSE*, and *siSERPING1* + *CSE* groups identified six distinct gene clusters. **f** Enrichment analysis of intersecting genes between Mfuzz cluster 3 and significantly upregulated genes; blue boxes highlight genes associated with inflammation and COPD pathogenesis. **g** Expression of *IL-6*, *IL-24*, and *IL-8* in si-SERPING1-transfected HFL1 cells stimulated with CSE, as measured via qRT‒PCR (*n* = 4 per group). **h** Schematic diagram illustrating the coculture system established between HFL1 human lung fibroblasts and macrophages differentiated from Tohoku Hospital Pediatrics-1 (THP-1) cells via PMA induction. qRT‒PCR analysis of cytokine expression: *IL-8* and *IL-24* in SERPING1-knockdown HFL1 fibroblasts cocultured with THP-1 macrophages under CSE + rilzabrutinib (**i**); *IL-8* and *IL-1β* in THP-1 macrophages from the coculture system (**j**) (*n* = 4 per group). **k** IL-8 and C3 secretion levels in fibroblast supernatants from the coculture system were quantified via ELISA (*n* = 4 per group). The data are expressed as the means ± standard deviations (SDs). *P* values displayed in charts were determined via two-tailed Student’s *t* tests (**a**–**c** and **e**), one-way ANOVA (**h**–**k**), and multiple paired *t* tests (**f**)

We established a cigarette smoke (CS)-induced COPD mouse model, and histopathological analysis confirmed successful induction of the disease (Fig. 5a). In the lungs of CS-exposed mice, the RNA expression of *Serping1* was increased, while its protein expression was decreased (Fig. 5b). In line with our murine data, human lung tissues (E-MTAB-8251) showed upregulated *SERPING1* mRNA but downregulated protein expression (Fig. 5c). Immunofluorescence costaining of the lung sections revealed a high degree of colocalization of SERPING1 and FN1, primarily in airway-adjacent interstitial cells and macrophages. In mice with COPD, the fluorescence intensity was markedly reduced in the peribronchial regions, which was characterized by decreased proportions of SERPING1^+^/FN1^+^ and SERPING1^+^/F4/80^+^ double-positive cells but an increased proportion of SERPING1^+^/ECAD^+^ cells (Fig. 5d). Consistently, analysis of human lung tissue sections revealed a similar pattern to the single-cell findings: *SERPING1* was broadly expressed across the lung, with reduced expression in peribronchial fibroblasts and alveolar septa but slightly increased expression in the airway epithelium of COPD patients (Supplementary Fig. 23c). Additionally, Serping1 strongly colocalized with C1qa and C1r, with increased proportions of C1r^+^, C1s^+^, and C1r^+^/C1s^+^ double-positive cells in CS-exposed mice (Supplementary Fig. 24a).

In human fetal lung fibroblast 1 (HFL1) cells, we determined the optimal cigarette smoke extract (CSE) stimulation conditions (0.6% for 48 h) (Supplementary Fig. 24b) and validated *SERPING1*-specific siRNA (Supplementary Fig. 24c, d). Transcriptome sequencing following siRNA transfection and CSE stimulation (Supplementary Fig. 24e) demonstrated via GSEA that *SERPING1* knockdown was positively correlated with inflammation-related pathways such as the *TNF-α*, *IL-6*, and *IL-2* signaling pathways (Supplementary Fig. 24f).

The genes were clustered into six groups on the basis of their dynamic expression trends across the experimental conditions (Fig. 5e). Intersection analysis of significantly up- and downregulated DEGs revealed upregulated genes predominantly in Cluster 3 (with 429 overlapping genes), whereas downregulated genes were localized in Clusters 1, 5, and 6 (with 277, 184, and 114 genes, respectively) (Supplementary Fig. 25a). The upregulated genes were enriched for inflammatory mediators (*IL-1β, IL-8, IL-24, IL-33, MMP-1*, and *MMP-3*) (Fig. 5f). The downregulated genes in Cluster 1 were associated with amino acid metabolism, those in Cluster 5 were associated with ECM/elastin degradation, and those in Cluster 6 were associated with metabolic pathways (Supplementary Fig. 25b–d). These findings indicate that *SERPING1* knockdown exacerbates CSE-induced inflammation (cluster 3), glycosylation barrier disruption (cluster 1), and ECM/elastic fiber degradation (cluster 5) in fibroblasts. Validation confirmed that *SERPING1* knockdown promoted the CSE-triggered upregulation of these inflammatory factors (with the exception of IL-33) (Fig. 5g, Supplementary Fig. 26a).

Given that previous CellChat results implicated fibroblast–macrophage crosstalk via complement signaling, we cocultured *SERPING1*-knockdown HFL1 cells with Phorbol 12-myristate 13-acetate (PMA)-differentiated THP-1 macrophages and administered the C1-specific inhibitor Riliprubart to the lower chambers (Fig. 5h). Riliprole suppressed the *SERPING1* knockdown-mediated increase in inflammatory factors in HFL1 cells (Fig. 5i, Supplementary Fig. 26b) and moderately reduced cytokine expression in cocultured macrophages (Fig. 5J, Supplementary Fig. 26c). Secreted IL-6 and IL-8 levels were reduced in both the HFL1 and THP-1 supernatants, with notably reduced secretion of C3 from the HFL1 cells (Fig. 5k, Supplementary Fig. 26d, e). These results indicate that *SERPING1* modulates inflammatory responses in fibroblasts and exerts downstream effects on macrophages, a process effectively suppressed by the C1s inhibitor Riliprole.

### *SERPING1* as a potential therapeutic target for CSE-induced COPD

**Fig. 6:**
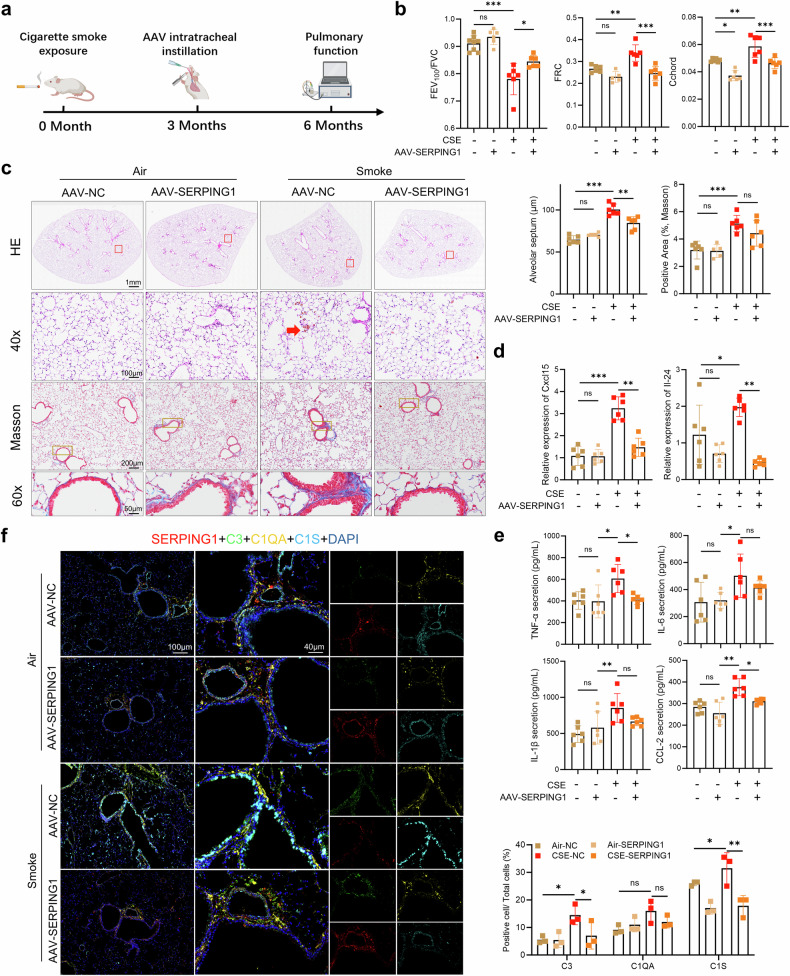
SERPING1-containing AAVs alleviate alveolar septal destruction and suppress inflammatory cytokines in CS-exposed mice. **a** Schematic diagram of AAV-mediated full-length *Serping1* gene therapy in CS-exposed mice. **b** Pulmonary function evaluation in four experimental groups: forced expiratory volume in 100 ms/forced vital capacity (FEV₁₀₀/FVC), functional residual capacity, and chord compliance (*n* = 6 per group). **c** Histopathological alterations in the lung tissues across the four experimental groups were observed via hematoxylin and eosin (H&E) and Masson’s trichrome staining (*n* = 6 per group). **d** Expression levels of *Cxcl-15* and *Il-24* mRNAs in the lung tissues of the four experimental groups (*n* = 6 per group). **e** IL-6, CCL-2, TNF-α, and IL-1β levels in lung tissue homogenates quantified by ELISA across four experimental groups (*n* = 6 per group). **f** Multiplex immunofluorescence showing the expression and colocalization of *SERPING1* (red), C3 (green), C1qa (yellow), and C1s (light blue) in mouse lung tissue sections from the four experimental groups. Bar plot: Quantification of C1s⁺-, C1qa⁺-, and C3⁺-positive cells relative to total cells (*n* = 3 per group). Nuclei were counterstained with DAPI (blue). The data are expressed as the means ± SDs. *P* values displayed in the charts were determined via one-way ANOVA (**b**–**e**) or two-way ANOVA (**f**)

To investigate SERPING1 function in vivo, a lung-specific adeno-associated virus (AAV) vector (LungM3) encoding full-length *Serping1* was constructed. The efficacy of AAV delivery was confirmed in mice (Supplementary Fig. 27a, b). In CS-exposed mice subjected to intratracheal instillation (Fig. 6a), treatment with AAV-*Serping1* improved lung function (Fig. 6b, Supplementary Fig. 27c) and attenuated alveolar septal destruction and inflammatory cell infiltration; however, it did not reduce collagen deposition (Fig. 6c). *Serping1* overexpression suppressed the pulmonary RNA levels of inflammatory mediators (*Cxcl-15* [human *CXCL-8* homolog], *Il-24*, *Il-6*, *Il-33*, *Tnf*, *Il-1b*, and *Ccl-2*; Fig. 6d, Supplementary 27d) and reduced the secretion of *Tnf* and *Ccl-2* (but not of *Il-6* or *Il-1β*) (Fig. 6e). Multiplex immunofluorescence demonstrated that AAV treatment increased *Serping1* expression in the peribronchial and alveolar regions, with significant peribronchial suppression of *C1s* and *C3* but not vascular endothelial C3 (Fig. 6f). These results indicate that *SERPING1* attenuates CS-induced pathology.

**Fig. 7:**
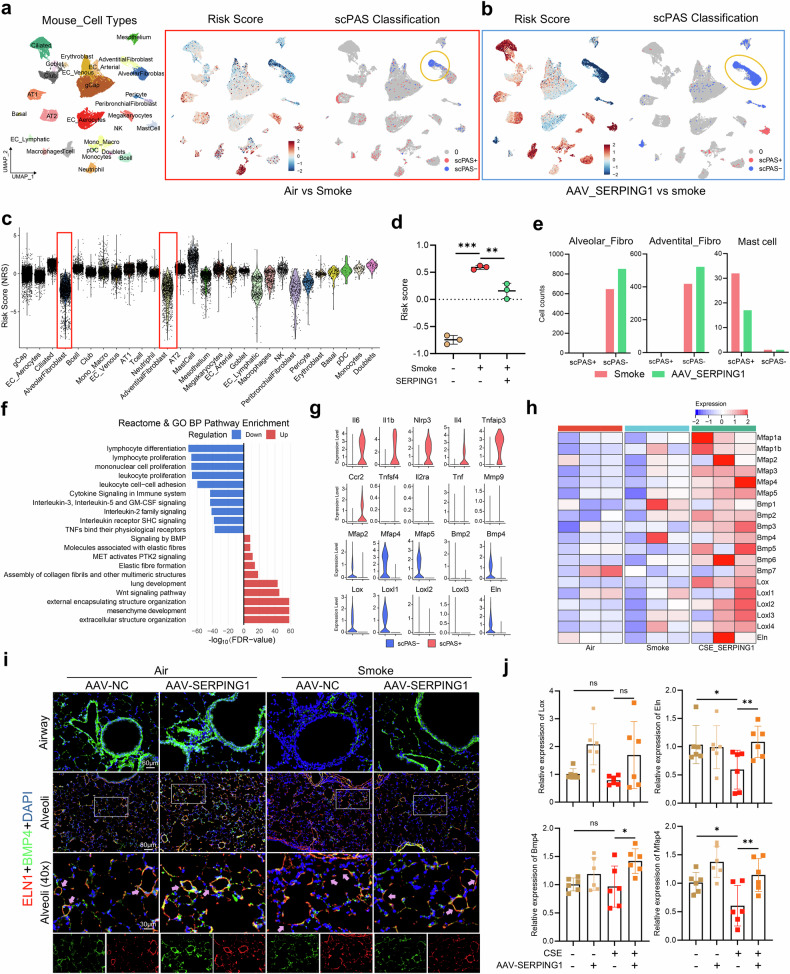
*SERPING1* Exhibits the Potential to Protect Pulmonary Elastin. **a** UMAP visualization of GSE168299 (left), single-cell phenotype-associated subpopulation identifier (scPAS)-derived risk scores (middle), and scPAS-selected cells (right) from murine lung tissue scRNA-seq data, comparing air-exposed versus CS-exposed mice. The colors reflect the risk score gradients (red: high; blue: low). **b** UMAP visualization of scPAS-derived risk scores (left) and scPAS-selected cells (right) from single-cell data from murine lung tissue comparing AAV-mediated *SERPING1* gene therapy-treated and control CS-exposed mice. Clusters denote therapeutic efficacy. **c** Distribution of scPAS-derived risk scores across air-exposed controls, CS-exposed mice, and AAV therapy-treated CS-exposed mice. Higher scores reflect elevated disease susceptibility gradients (*n* = 3 per group). **d** Violin plot comparing scPAS-calculated risk scores across distinct cell types. **e** Quantitative changes in the number of scPAS-negative and scPAS-positive alveolar fibroblasts, adventitious fibroblasts, and mast cells in the CS-exposed group versus the AAV-treated group. **f** Reactome and GO enrichment of DEGs (*FDR* < 0.05, *fold change* [*FC*] ≥ 0.25) in scPAS-positive versus negative cells; bar length = *-log₁₀ (FDR)*. **g** Violin plots demonstrating the expression distributions of inflammation-related and elastin-related genes from enriched pathways in scPAS-positive versus negative cells. **h** Heatmap displaying the expression levels of the Mfap family, Bmp family, Lox family, and Eln genes in air- and CS-exposed and AAV-treated mice. **i** Multiplex immunofluorescence showing the expression of elastin (Eln1; red) and Bmp4 (green) in mouse lung tissue sections from the four experimental groups. Nuclei were counterstained with DAPI (blue). **j** qRT‒PCR analysis of *Lox*, *Eln*, *Bmp4* and *Mfap4* mRNA in the lung tissues of the four experimental groups. (*n* = 6). The data are expressed as the means ± SDs. *P* values displayed in the charts were determined via one-way ANOVA (**D**, **J**)

RNA-seq of mouse lung tissue (Supplementary Fig. 27e) revealed enrichment of complement pathway genes via GSEA (Supplementary Fig. S27f), which is consistent with previous results and indicates that *SERPING1* modulates complement activation. To identify the specific cell subpopulations targeted by AAV therapy, we integrated bulk-seq and single-cell data via single-cell phenotype-associated subpopulation identifiers (scPASs). In the CSE group compared with the air group, neutrophils, AT1 cells, T cells, and macrophages were positively correlated with the CSE risk score, whereas adventitial fibroblasts were negatively correlated with the CSE risk score (Fig. 7a, Supplementary Fig. 27g). In the comparison of the CSE- and AAV-treated groups, adventitial and alveolar fibroblasts (demonstrating the strongest negative effect), the mesothelium, and pericytes were negatively correlated with CSE risk but positively correlated with treatment, whereas mast cells were positively correlated with CSE risk (Fig. 7b, c). AAV treatment significantly reduced CSE risk scores (Fig. 7d) and resulted in an increase in scPAS⁻ (negatively linked to CSE risk) adventitial and alveolar fibroblasts, whereas scPAS⁺ (positively linked to CSE risk) mast cells decreased (Fig. 7e). These findings confirm that lung fibroblasts are the primary targets of AAV, which is consistent with our hypothesis.

Notably, enrichment analysis of the scPAS⁺ and scPAS⁻ DEGs revealed that the downregulated genes were enriched in inflammatory pathways, whereas the upregulated genes were associated with elastic fiber function, BMP/Wnt signaling, and pulmonary/stromal development (Fig. 7f). scPAS⁺ cells highly expressed *Il-6*, *Il-1β*, and *Il-4*, whereas scPAS⁻ cells upregulated genes involved in elastic fiber assembly, such as *Mfap4*, *Bmp4*, and *Eln* (Fig. 7g, Supplementary Data 40). RNA-seq revealed that AAV treatment increased the expression of elastic fiber-associated genes in lung fibroblasts and mouse tissue (Fig. 7h), whereas *SERPING1* knockdown exacerbated CSE-mediated downregulation of these genes in HFL1 cells (Supplementary Fig. 27h).

The integration of bulk RNA-seq data with human lung scRNA-seq data via scPAS mirrored the mouse findings (Supplementary Fig. 28a). Here, scPAS⁺ cells were chiefly macrophages and monocytes—supporting SERPING1-mediated fibroblast–macrophage complement interactions (Supplementary Fig. 28b)—and scPAS⁻ cell proportions were reduced in COPD samples (Supplementary Fig. 28c). The genes whose expression was upregulated in the scPAS⁺ cells compared with that in the scPAS⁻ cells were related primarily to inflammation, whereas the genes whose expression was downregulated were enriched for secreted proteins and extracellular matrix components, mirroring the mouse results (Supplementary Fig. 28d). Human lung scRNA-seq further confirmed the decreased expression of elastic fiber genes in MSCs and lung mesenchymal cells (LMCs) from COPD patients, whereas SERPING1-high MSCs/LMCs presented increased expression (Supplementary Fig. 28e, f).

Immunofluorescence in smoke-exposed wild-type mice revealed reduced Bmp4 expression around airways and disrupted alveolar-septal elastin integrity, with septal Bmp4 unaltered. AAV treatment partially restored peribronchial Bmp4 and preserved septal elastin integrity (Fig. 7i) and partially rescued the lung mRNA levels of *Eln*, *Bmp4*, and *Mfap4* (Fig. 7j). Together with SERPING1 having the highest abundance in mesenchymal stem cells (Supplementary Fig. 23b), these data suggest a protective, reparative role for Serping1 in alveolar septa, although the precise molecular mechanisms involved remain to be elucidated.

## Discussion

This study integrated COPD and pulmonary function data from multiple GWASs with multicohort eQTL and pQTL evidence. By employing diverse MR approaches to establish an evidence hierarchy, we identified a potential link between elevated *SERPING1* levels and reduced COPD risk as well as improved lung function. Further integration of the UKB and ECOPD cohort resources with single-cell sequencing data from patients with COPD and murine models combined with multidimensional GWAS, in vitro cellular experiments, and in vivo animal studies revealed the functional role of SERPING1 in lung fibroblasts and its contribution to COPD progression.

Given the substantial heterogeneity of COPD across populations, we integrated GWAS data from multiple independent cohorts, encompassing both COPD diagnoses and pulmonary function metrics, with cross-referenced analyses of published eQTL/pQTL databases. Our analyses revealed variations in results across different QTL datasets and cohort-specific GWAS data, indicating that multisource data integration is the optimal approach for mitigating inherent COPD heterogeneity and intercohort discrepancies. By implementing integrated MR approaches, including SMR, TSMR, and colocalization analyses, we established a tiered evidence system to prioritize genes with core regulatory functions at both the RNA and protein levels during COPD progression, a methodology previously validated for other diseases.^16^

Consistent with our hypothesis, MR analyses of COPD samples revealed significantly greater heterogeneity across the GWAS and QTL datasets than did pulmonary function metrics. Substantial variation was observed in the results derived from different analytical combinations, with notably fewer Tier 1–evidenced genes identified for COPD than for pulmonary function traits. This pattern underscores substantial genomic divergence in COPD patients diagnosed with pulmonary function criteria. Conversely, pulmonary function analyses revealed overlapping Tier 1 genes across the datasets. Integrative assessment of Tier 1 genes from diverse analytical combinations revealed established COPD-associated genes, including *SERPINA1*,^17^ *SCARF2*,^12^ and *SERPINF1*,^18^ confirming their methodological reliability. Furthermore, this approach identified novel genes, such as *ASPSCR1, TNFRSF6B*, and *TRIM25*, which are linked to COPD pathogenesis and pulmonary function decline, warranting further experimental validation to demonstrate their functional roles in COPD progression.

*SERPING1* encodes C1 esterase inhibitor (C1-INH), a member of the serine protease inhibitor (serpin) superfamily. As a key regulator of complement and contact system activation, C1-INH functions as an anti-inflammatory protein.^19^ It maintains immune homeostasis by inhibiting C1r/C1s proteases and kallikrein, thereby modulating bradykinin production and vascular permeability.^20^ Although extensively studied in hereditary angioedema, SERPING1 mutations cause C1-INH deficiency, leading to disease pathogenesis.^21^ *SERPING1* has also demonstrated therapeutic efficacy in animal models of multiple inflammatory disorders.^22^

Emerging evidence implicates *SERPING1* in pulmonary disease. For example, *SERPING1* expression is significantly reduced in the peripheral serum of patients with lung adenocarcinoma (LUAD), and experimental studies have confirmed its role in suppressing LUAD progression via the mTOR pathway.^23^ Additionally, *SERPING1* alleviates bleomycin-induced lung injury.^24^ Despite MR studies reporting associations between *SERPING1* and pulmonary function,^25^ the prognostic significance and mechanistic role of *SERPING1* in COPD remain unclear.

By integrating multiple pQTL datasets and a cohort GWAS, we established significant causal associations between *SERPING1* and both pulmonary function decline and COPD risk. Notably, *SERPING1* was not associated with IPF and showed only minimal association with asthma, highlighting its potential as a realatively specific therapeutic target for COPD. Importantly, *SERPING1* expression in peripheral blood was significantly negatively correlated with the rate of decrease in FEV₁ in both the UKB cohort and the ECOPD cohort. Since accelerated FEV₁ decline (≥60 ml/year) has been widely recognized as a marker of early COPD and a predictor of increased risk for disease progression and mortality, *SERPING1* may serve as a biomarker for the early identification of high-risk individuals.^26^ Given its consistent association with short-term lung function decline but lack of significance at longer-term follow-up, *SERPING1* may be more suitable as a short-term biomarker than for long-term prognosis. Furthermore, we observed ethnic differences in basal *SERPING1* levels: Europeans presented higher baseline SERPING1 expression and further upregulation in COPD patients, whereas East Asians presented lower baseline SERPING1 expression with further downregulation in disease patients. This divergence was consistent across age groups and smoking statuses, suggesting potential population-specific regulatory mechanisms influencing COPD pathogenesis.

Furthermore, colocalization analysis identified a novel SNP, rs28362951, which was strongly associated with *SERPING1* expression, pulmonary function, and COPD risk. The homozygous mutant genotype correlated with reduced *SERPING1* expression, decreased baseline pulmonary function, and accelerated FEV_1_ decline in the UKB cohort. Although the minor allele frequency of rs28362951 is greater in Europeans than in Asians, the prevalence of homozygous mutations across ethnic groups remains uncharacterized. Critically, *rs28362951* does not account for basal *SERPING1* expression differences between European White and East Asian populations. Given the elevated *SERPING1* RNA levels in the lung tissues and peripheral blood of European patients with COPD, this ethnic disparity likely arises from transcriptional regulatory mechanisms. Resolving this hypothesis requires a large-scale comparative analysis of Asian cohorts.

*SERPING1* and complement family expression occur in diverse pulmonary cells, particularly interstitial cells, which is consistent with our findings.^25^ Integrated multiomics analysis^14^ revealed that lung fibroblasts are the primary effectors of *SERPING1*. In addition to autonomous inflammatory modulation, *SERPING1* regulates macrophage complement activation via C3 secretion. Lung-specific *Serping1*-AAV attenuates alveolar destruction and suppresses cytokine expression in CSE-treated mice. Murine lung mapping confirmed that fibroblasts are key AAV targets, confirming the fibroblast-centric function of SERPING1. Notably, *SERPING1*-high mesenchymal cells and scPAS⁺ fibroblasts (inversely linked to CSE risk and positively linked to AAV treatment) upregulated elastin assembly genes. Mfap4 knockout mice spontaneously develop emphysema,^27^ and plasma MFAP4 serves as a potential COPD biomarker;^28^ pan-*LOX* inhibition disrupts peribronchial collagen I organization, impairing functional integrity;^29^ and *BMP4* suppresses fibroblast-to-myofibroblast differentiation by reducing autophagy and cellular senescence.^30^ Additionally, the finding that *SERPING1* is expressed at the highest level in MSCs within fibroblasts suggests that it has unexplored repair functions, which requires further experimental validation. Endothelial cells also correlated with the AAV treatment groups, which is consistent with the reported roles of SERPING1 in vascular regulation.

However, this study has several limitations. *SERPING1* was not significantly associated with the rate of decrease in lung function in the UKB visit 2 cohort, indicating that its viability as a long-term indicator requires further investigation. The underlying causes of differential baseline expression and disease-associated changes in *SERPING1* between European and Asian populations remain unexplained. We did not elucidate the direct molecular mechanisms by which *SERPING1* regulates elastin-associated genes in lung tissue. Future studies should employ *SERPING1* fibroblast-specific knockout mice and pulmonary fibroblast-tracing models to construct CS-exposed mouse models, thereby clarifying the role of SERPING1 in COPD progression.

Overall, we integrated multiple QTL datasets with independent cohorts, employed multimodal analytical approaches, and performed multiscale cross-validation analyses with evidence-based gene prioritization. By leveraging both the UKB and ECOPD cohorts, we demonstrated that peripheral blood *SERPING1* expression is a potential biomarker for predicting lung function decline. Furthermore, AAV-mediated interventions in CS-induced mouse models validated the therapeutic potential of *SERPING1* as a COPD-specific biological target. Notably, *SERPING1* expression was significantly lower in Asians than in Europeans under baseline conditions and further decreased during disease progression, whereas it increased in Europeans with COPD. This raises the question of whether *SERPING1* is a more suitable therapeutic target for Asian patients with COPD than for European patients.^10^

## Methods and materials

### GWAS summary statistics for eQTLs and pQTLs of genes, COPD risk, and lung function

Summary statistics for COPD susceptibility^31,32^ and lung function parameters, including FEV₁, FEV_1_% predicted, and FVC ratio^33,34^ were acquired from the *GWAS Catalog* (https://www.ebi.ac.uk/gwas/), the *Integrative Epidemiology Unit* (*IEU*) *GWAS database* (https://gwas.mrcieu.ac.uk/),^35^ and *the FinnGen study* (freeze 10, https://r10.finngen.fi/). The discovery-phase datasets were identified via the following accession codes: *ieu-b-106*, *GCST90244092*, *ukb-a-235*, and *GCST90244094*. The validation cohorts included *FinnGen_COPD, ukb-b-19657*, *ukb-b-13405*, and *GCST90029026*. *The* cis-eQTL data were obtained from the *eQTLGen* Consortium^36^ and the *CAGE* database,^37^ whereas the cis*-*pQTL data were derived from four datasets: *Decode*,^38^ *UKB-PPP*,^39^ *Finngen_R10_olink*, and *Finngen_R10_Somascan*.^40^ These were generated by integrating Olink and SomaScan sequencing data (from the Decode, UK Biobank (UKB), and FinnGen studies) with genomic sequencing-converted pQTLs. Four more independent cohorts were included: EA et al.,^41^ Gudjonsson_A et al.,^42^ Sun_BB et al.,^41^ and Pietzner et al.^43^

pQTL summary statistics were transformed into Summary-data-based Mendelian Randomization (SMR) BESD format in a two-step process. First, per-protein cis-association results were exported as tab-delimited ESD files and cataloged in a single “.flist” index file. Second, we used SMR software (https://yanglab.westlake.edu.cn/software/smr/) to convert this index into a sparse BESD file and its associated *.epi/.esi* indices, applying a ± *1* *Mb* cis window and *P* < 1 × 10⁻⁵ threshold. The detailed BESD format specifications are available at https://cnsgenomics.com/software/smr/download/SMR.pdf. Methylation QTL (mQTL) summary data were obtained from the SMR data resource repository (https://yanglab.westlake.edu.cn/software/smr/#DataResource).^44^ All instruments and variables for subsequent two-sample MR analyses of xQTLs and target genes were extracted directly from these SMR-formatted files. Finally, all instrumental variables (IVs) for two-sample MR analyses of target‒gene xQTLs were extracted directly from these SMR-formatted files.

The analyses were restricted to individuals of European ancestry with 1000 genome-imputed genotypes. All the data used are publicly accessible GWAS summary statistics. The original study provided written informed consent and ethical approval.

### Study population in the UKB and ECOPD cohorts

The UKB is a population-based prospective cohort with longitudinal proteomic profiling and phenotypic characterization and has been approved by the Northwest Multicenter Research Ethics Committee (Ref: 16/NW/0274). All participants provided written informed consent, and analyses were conducted under application *ID 150455*. From 53,013 baseline participants with available proteomic profiles, individuals lacking essential physiological parameters or baseline spirometry data were excluded. Lung function outliers were removed via a 99% confidence interval, resulting in the exclusion of 6644 individuals and yielding a final analytical cohort of 46,369 subjects.

COPD diagnosis was defined via International Classification of Diseases (ICD) codes, identifying 1907 patients with COPD and 39,485 non-COPD controls, with an additional 4977 individuals lacking ICD codes. For each visit, the FEV₁/FVC ratio was calculated from the highest recorded FEV₁ and FVC values. Normal lung function was defined as an FEV₁/FVC ratio ≥ 70% and an FEV₁% that was ≥80%, whereas impaired lung function was defined as either an FEV₁/FVC ratio < 70% or an FEV₁% that was <80%. Demographic and clinical characteristics are provided in Data S23. External validation was conducted using the ECOPD cohort (309 controls and 267 COPD patients), of which 237 controls and 197 patients with COPD completed a 3-year follow-up assessment.^45^ The baseline characteristics are presented in Data S29. The rate of lung function decline was calculated as follows: (baseline measurement − follow-up measurement)/follow-up duration.

#### gsMAP

Spatial transcriptomic datasets for human (STDS0000114) and murine (STDS0000062) tissues were sourced from the STOmics Database.^46–48^ Cellular subpopulation clustering followed original unsupervised frameworks. Cell type reclassification leverages metadata-documented subgroup-specific marker genes. Trait-associated cells were mapped via gsMAP,^14^ which integrates ST data with GWAS summary statistics. Preprocessing uses graph neural networks (GNNs) to cluster spatially adjacent spots with homogeneous expression. Gene specificity scores (GSSs) were computed by comparing local cluster gene ranks against global tissue expression and then mapped to SNPs (±100 kb from the TSS) via Roadmap Epigenomics chromatin annotations. S-LDSC was used to evaluate SNP-level pleiotropic enrichment (*p* < 0.05). Cross-species validation was used to align mouse/macaque ST datasets to human loci via LiftOver and Procrustes transformation.

#### seismicGWAS

Cell type‒trait associations were analyzed via seismicGWAS.^13^ scRNA-seq data, which were formatted as single-cell experimental objects, underwent *log2* ^*(CPM + 1)*^ normalization. Cell type-specific gene expression scores were computed via a modified Jensen–Shannon divergence (JSD) metric, accounting for background transcriptional variability (10,000 bootstraps). Multi-marker Analysis of GenoMic Annotation (MAGMA) derived gene-level associations from GWAS summary statistics (LD reference: 1000 Genomes Phase III EUR).^49^ Weighted least squares regression was used to correlate the MAGMA z scores with the cell type-specific expression profiles, adjusting for gene length, GC content, and covariates. Significant associations (*p* < *0.05*) were subjected to influential gene analysis, where Cook’s distance >4/N identified driver genes.

### scPAS

The scPAS framework identified phenotype-associated subpopulations in murine lung scRNA-seq data. A gene‒gene interaction network was constructed via Pearson correlations (|*r*| > 0.25) and shared nearest neighbors (k = 20). A network-regularized sparse regression (NRSR) model trained on single-cell data combined *L*_1_-penalized feature selection (α = 0.3) with topology-aware regularization is used to align coefficients with network connectivity. The parameters (*λ* = *0.05*) were optimized via 10-fold cross-validation. Phenotype association scores (PASs) were computed per cell, followed by permutation testing (500 iterations) to derive normalized PASs (*|NPAS*| > *1.96, p* < *0.05*). scPAS^+^/scPAS^−^ cells were identified, with pulmonary fibroblasts showing transcriptional profiles congruent with or inversely correlated with sequencing-derived signatures.

### Statistical analyses

Statistical analyses were performed via the Statistical Package for the Social Sciences (version 27.0), GraphPad Prism (version 10.0.1), and R (version 4.2.2) software. Clinical associations were evaluated via correlation analysis, univariable regression, and multivariable regression models. The UKB multivariable model was adjusted for age, BMI, sex, ethnicity, smoking status, alcohol consumption, and income, whereas the ECOPD model was adjusted for age, BMI, sex, smoking status, and pack-years. Logistic regression was used to analyze binary outcomes (COPD/impaired lung function), whereas linear regression was used to assess continuous outcomes (lung function, questionnaire scores, and computed tomography [CT] metrics). Differences between paired means were evaluated via two-tailed Student’s *t* tests, and comparisons among multiple groups were conducted via one-way analysis of variance (ANOVA). Significance levels are indicated as follows: ns (not significant) for *p* ≥ 0.05, *p < 0.05, **p < 0.01, and ***p < 0.001.

## Supplementary information

Sigtrans_Supplementary_Materials_Word

Raw band for Western Blod

Supplemental Data S1-S10

Supplemental Data S11-S18

Supplemental Data S19-S122

Supplemental Data S23-S36

Supplemental Data S37-S41

## Data Availability

All transcriptome sequencing and mass spectrometry identification data referenced in our manuscript are available in the CNCB-NGDC database under BioProject ID PRJCA043817 and OMIX IDs OMIX011185 and OMIX011186. Additional publicly available sequencing data were obtained from the GEO and ArrayExpress databases. The GWAS summary statistics used in this study are available in Supplemental Data 1 and 2.
