# Supplementary material for: Multiomics Mendelian randomization identifies serpin family G member 1 as a chronic obstructive pulmonary disease modulator
Source: Signal Transduct Target Ther. 2026 Jan 21;11:34. doi: 10.1038/s41392-025-02547-7 (PMC12819415; doi:10.1038/s41392-025-02547-7)
Supplement: Supplementary file 1 — Sigtrans_Supplementary_Materials_Word [file 41392_2025_2547_MOESM1_ESM.docx]

Supplementary Materials for

**Multiomics Mendelian randomization identifies serpin family G member 1 as a chronic**

**obstructive pulmonary disease modulator**

Erkang Yi^1,2#^, Jieda Cui^1,2#^, Hairong Wang^2#^, Fan Wu^1,2#^, Qiyang Hong^3#^, Qingyang Li^4^, Chengshu Xie^2^, Huahua Xu^1,2^, Yu Liu^1,2^, Xinru Ran^5^, Xiaohui Wu^1^, Qi Wan^1^, Gaoying Tang^1^, Leqing Zhu^1,2^, Junling Pang^3^, Yumin Zhou^1,2^, Erping Long^3*^, Pixin Ran^1,2*^

Correspondence to: pxran@gzhmu.edu.cn

**This PDF file includes:**

Materials and Methods

Figures. S1 to S28

Captions for Data S1 to S41

**Other Supplementary Materials for this manuscript include the following:**

Data S1 to S10

Data S11 to S18

Data S19 to S22

Data S23 to S36

Data S37 to S41

Raw band for Western Blod

Materials and Methods

Genetic correlation of the pQTL of *SERPING1* with COPD and lung function parameters using LD score regression (LDSC)

To quantify the genetic covariance between extra-pulmonary *SERPING1* pQTLs, COPD susceptibility, and pulmonary function indices, we implemented a cross-trait linkage disequilibrium score regression (LDSC) framework^1^ (LDSC, https://github.com/bulik/ldsc/wiki/Heritability-and-Genetic-Correlation). This analytical approach evaluated genome-wide genetic correlations across 47 chronic inflammatory disorders, COPD phenotypes (FEV_1_/FVC ratio, FEV_1_% predicted), and spirometric parameters. The algorithm leveraged pre-computed European-ancestry LD scores derived from 1,201,235 HapMap3^2^ SNPs (MAF >5%, imputation quality INFO >0.9). Genetic correlation coefficients (rg) were scaled between -1 and 1 using the HapMap3 reference panel, with statistical significance thresholded at two-sided *p < 0.05*.

Selection of Instrumental Variables (IVs) of MR Analysis

Genetic variants serving as exposure proxies were extracted from eQTL/pQTL datasets under the following criteria: (1) genome-wide significant association with target exposures (threshold: *P < 5×10⁻⁸*); (2) linkage disequilibrium independence (*pairwise r² < 0.01 within 500 kb windows*). Variants demonstrating weak instrument bias (*F-statistic < 10*) were systematically excluded. Reverse MR analyses were subsequently implemented by redefining COPD phenotypes and pulmonary function measures (FEV_1_/FVC, FEV_1_% predicted) as exposures, with corresponding eQTLs/pQTLs as outcomes. This analytical phase employed stricter LD clumping parameters *(r² < 0.001, physical distance > 10000 kb*) while maintaining equivalent significance thresholds. Heterozygosity status for candidate SNPs was annotated via the Ensembl browser (release 110, https://www.ensembl.org).

Summary-data-based Mendelian (SMR) randomization analyses

We performed SMR and Heterogeneity in Dependent Instruments (HEIDI) analyses within cis-regulatory regions using the SMR software (https://yanglab.westlake.edu.cn/software/smr/#Overview)^3^. This methodology, based on the original SMR framework, employs a single nucleotide variant (SNV) at a primary cis-eQTL as an instrumental variable (IV). Leveraging summary-level eQTL, pQTL, mQTL and GWAS data, this IV enabled investigation of potential causal or pleiotropic associations between gene expression and target traits. While SMR alone cannot definitively distinguish causal from pleiotropic associations due to its reliance on a single IV, the HEIDI test supplements this by differentiating causality from linkage. We further extended these analyses using summary-level xQTL data as exposures. Standard SMR software parameters were maintained, including a p-value threshold of *5.0 × 10⁻⁸* for top eQTL selection and a ±1 Mb window centered on the probe for cis-eQTL identification. Consistent with single-probe constraints, SMR analyses were restricted to cis regions with statistical significance defined as *p ≤ 0.05*. For HEIDI, *p < 0.05* indicated significant linkage, rejecting a purely causal association.

Statistical analysis of MR Analysis

For the Mendelian Randomization (MR) analysis, the Wald ratio was employed for proteins with a single genetic instrument. In contrast, the inverse variance weighted MR (MR-IVW) method was utilized for proteins with multiple applicable instruments, accompanied by heterogeneity analysis, and the MR-Egger Intercept was applied to evaluate horizontal pleiotropy, with a *p-value > 0.05* indicating minimal pleiotropy. Cochran’s Q Statistic was utilized to detect heterogeneity in IV associations, where a *p-value > 0.05* signified non-heterogeneity and justified the use of a fixed-effects model, while a *p-value < 0.05* indicated heterogeneity, necessitating a random-effects model. The MR-PRESSO Global Test was employed to detect and correct for horizontal pleiotropy by identifying and adjusting for outliers when significant (*p < 0.05*). Finally, Leave-One-Out Analysis was conducted to examine the influence of individual SNPs on the overall estimate, ensuring that the results were not driven by any single SNP. Multivariable Mendelian Randomization (MVMR)^4^ was employed to evaluate the causal relationships between multiple exposures simultaneously. This approach allows for the adjustment of potential confounding factors, providing a more comprehensive understanding of the causal pathways involved. MVMR utilizes multiple genetic instruments for each exposure and assesses their combined effects on the outcome of interest.

Mediation MR analysis

We implemented two-step Mendelian randomization (https://mr-dictionary.mrcieu.ac.uk/term/two-step/)^5^ to assess mediation effects within an MR framework. Step 1 involved univariable MR examining associations among exposure, outcome, and mediators, specifically applying inverse variance weighted (IVW) MR to estimate causal effects of: (i) smoking (exposure) on *SERPING1* pQTL levels (mediator); and (ii) *SERPING1* pQTL (mediator) on COPD susceptibility and lung function indices (outcomes).

Colocalization analysis

We implemented COLOC algorithm (https://chr1swallace.github.io/coloc/index.html)^6^ to evaluate shared genetic causality between *SERPING1* pQTL and COPD/lung function phenotypes at identical loci. This Bayesian framework computes posterior probabilities for five scenarios: H0 (null association); H1 (causal variant exclusive to pQTL); H2 (causal variant exclusive to COPD/lung function); H3 (distinct variants per trait); H4 (shared causal variant). Using coloc R package (v5.1.0), we quantified H4 posterior probabilities (PP) for colocalized SNPs, restricting analysis to ±1 Mb windows around lead GWAS variants aligned with European LD patterns. Statistical evidence required *PP ≥0.70*.

Multi-trait colocalization (moloc) evaluated shared causal variants across eQTL, pQTL, and COPD phenotypes within *1 Mb* regions. The Bayesian framework calculates posterior probabilities (PPA) for five models: H1 (distinct variants); H2 (shared eQTL-pQTL variant + independent COPD variant); H3 (shared pQTL-COPD variant + independent eQTL variant); H4 (shared eQTL-COPD variant + independent pQTL variant); H5 (single causal variant for all traits). Analyses prioritized H2-H5 with *PPA >0.5* (moderate) and *>0.7* (strong) evidence thresholds, integrating regional LD patterns and effect size correlations.

HyPrColoc evaluated pleiotropic mechanisms linking *SERPING1* eQTLs, pQTLs, and mQTLs to COPD/pulmonary function. This Bayesian framework computes PP for causal variant co-occurrence across molecular traits and clinical phenotypes, adopting PP >0.7 significance threshold within *±500 kb* genomic windows centered on lead variants, incorporating LD patterns and allelic effect concordance.

Pleiotropy-informed conditional false discovery rate (*PleioFDR*) analysis and conditional FDR (*condFDR*) method

We implemented pleiotropy-informed conditional false discovery rate (pleioFDR) methodology^7^  to detect genetic overlap between *SERPING1* (primary trait) and COPD (secondary trait) by jointly applying conditional FDR (condFDR) and conjunctional FDR (conjFDR) analyses. The condFDR method computationally incorporates auxiliary trait associations using empirical Bayesian inference, amplifying SNP detection power with genome-wide significance defined at FDR <0.01. Pleiotropic enrichment was visualized through conditional quantile-quantile plots; progressive leftward divergence from the null distribution across secondary trait association quantiles indicate enhanced true discovery rates (*1-FDR*) proportional to cross-phenotype effect sizes.

The conjFDR method identified loci associated with both *SERPING1* and COPD, assessing the posterior probability that SNPs show concurrent associations under joint phenotypic p-value thresholds. Statistical significance required condFDR <0.01 and conjFDR <0.05. While condFDR prioritizes novel locus discovery under strict type I error control, conjFDR tolerates higher false positive rates to detect pleiotropic mechanisms, thus detecting shared biological pathways.

Gene expression, RNA-seq analysis and Bioinformatics

Peripheral blood RNA-seq datasets for COPD (GEO accessions: GSE76705^8^, GSE71220^9^) and lung tissue transcriptomes (GSE47460^10^, GSE76925^11^)  were acquired from the NCBI GEO repository. Functional annotation of overlapping genes was performed through Metascape (https://metascape.org) ^12^ and Clusterprofiler v4.0^13^, integrating Kyoto Encyclopedia of Genes and Genomes (KEGG) pathway analysis with Gene Ontology (GO) biological process enrichment. The lung tissue proteome data (E-MTAB-8251) were obtained from the ArrayExpress database. RNA sequencing was conducted using the NovaSeq™ X Plus platform (Illumina) at Wekemo (Shenzhen, China), with raw reads aligned to the Ensembl GRCh38.v102 reference transcriptome. Differential expression analysis implemented the Limma R package, utilizing linear models with empirical Bayes moderation to identify genes exhibiting significant expression changes (*p* <0.05).

Mfuzz

Groups-series transcriptomic data were clustered using Mfuzz^14^ based on ​fuzzy c-means algorithm​ to identify co-expression patterns. Expression matrices (genes × time-ordered samples) were normalized to ​log₂(CPM/TPM)​, filtered for missing values (>25% threshold), imputed via ​k-nearest neighbors, and Z-score standardized. Optimal cluster number (c) was determined by ​Dmin elbow-point analysis​ (c = 6–20 range), while fuzzifier parameter (m) was auto-calculated using mestimate. Clustering executed with mfuzz() generated membership matrices (0–1), with core genes (membership >0.7) extracted via acore. Cluster trajectories were visualized by mfuzz.plot() with color gradients indicating membership strength. Functional enrichment of cluster-specific genes used clusterProfiler (*p <0.05*)

.

Single-cell sequencing and analysis of mouse model

The integrated publicly available single-cell RNA sequencing (scRNA-seq) dataset was obtained from the GEO database (accessions: GSE173896^15^, GSE171541^16^, GSE227691^17^ and GSE167295^18^), comprising 16 control and 20 COPD patient samples. This integrated dataset encompassed 156,483 cells and 40,714 genes. ​Single-cell RNA sequencing data of lung tissues from cigarette smoke-exposed mice were sourced from the GEO dataset (accession GSE168299)^19^. The R environment and Seurat package (https://satijalab.org/seurat/)^20^ were utilized for unsupervised clustering and cell annotation of single-cell RNA-seq data, as well as for data analysis and visualization. The standard pipeline included data normalization, dimensionality reduction, and clustering following established protocols. Differentially expressed genes (DEGs) across cell subsets were identified and subjected to enrichment analysis via Metascape. Intercellular communication was assessed using CellChat (v1.1.3). Visualization of single-cell analysis results was implemented with ggplot2. The pseudotime analysis was validated using the "Monocle 2" R package.

Cell culture and co-culture

Human lung fibroblast 1 (HFL1) cells were sourced from ATCC (CCL-153, USA), which was cultured in F-12K medium (Kaighn's modification of Ham's F-12) supplemented with 10% FBS (Gibco) and 1% P/S (Invitrogen). THP-1 cells obtained from Procell (Wuhan, China) were maintained in 1640 medium containing 0.05 mM β-mercaptoethanol, 10% FBS, and 1% P/S. To differentiate THP-1 cells into M0 macrophages, they were treated with 100 ng/ml PMA as previously described^21^. In the co-culture setup, HFL1 cells were placed in the lower chamber of a transwell for transfection, while THP-1 cells were added to the upper chamber to facilitate differentiation and adherence. After 48 hours of transfection and 72 hours of differentiation, THP-1 cells were co-cultured with HFL1 cells, and recombinant S100A9 (rp-S100A9) was introduced into the lower chamber culture. All primary cells and cell lines were incubated at 37°C in a humidified atmosphere with 5% CO2.

RNA extraction, complementary deoxyribonucleic acid synthesis, and quantitative real-time polymerase chain reaction

RNA extraction from whole blood was carried out using a commercially available RNA isolation kit, adhering to the manufacturer's instructions^22^. For complementary DNA synthesis, a reverse transcription kit suitable for quantitative polymerase chain reaction (qPCR) was employed, with 1,000 ng of total RNA as the input material. Quantitative real-time PCR (qRT-PCR) was performed using a SYBR Green-based PCR master mix on an RT-PCR detection system. Gene expression levels were analyzed using the comparative CT (2^–∆∆CT^) method, with Glyceraldehyde-3-Phosphate Dehydrogenase (GAPDH) serving as the endogenous control. A comprehensive list of primer sequences utilized in this study can be found in Supplementary Data 41.

Western blot (WB)

Western blot analysis was conducted using established protocols^23^. Briefly, cells or lung tissues were lysed in RIPA lysis buffer (89901, Thermo, USA) supplemented with a protease inhibitor cocktail (78430, Thermo, USA) at 4°C for 20 minutes. Protein extracts were then separated by 10% SDS-polyacrylamide gel electrophoresis and transferred to polyvinylidene difluoride (PVDF) membranes (Bio-Rad, USA). After blocking, the membranes were incubated overnight at 4°C with antibodies against S100A9 (26992-1-AP, Proteintech, China), p-p65 (ab76302, Abcam, UK), p65 (ab32536, Abcam), p38 MAPK (#8690S, CST, USA), Phospho-p38 MAPK (#4511S, CST), Actin (23660-1-AP, Proteintech, China), and GAPDH (10494-1-AP, Proteintech, China). Following washing, membranes were incubated with a peroxidase-conjugated secondary antibody (Proteintech). Protein bands were visualized using chemiluminescence on an Amersham Imager 680 (Thermo Fisher Scientific).

Clinical sample collection

Peripheral blood samples were obtained from participants enrolled in the Early COPD (ECOPD) study (Chinese Clinical Trial Registry, ChiCTR1900024643)^24^ and randomly selected for inclusion in this analysis. Data from a subset of these participants has been reported previously. Venous blood was collected by trained personnel into EDTA-containing tubes, centrifuged at 3,000 rpm for 10 minutes at room temperature, and the resulting plasma was aliquoted and stored at –80 °C until further use.

Lung tissue samples from patients with COPD and from relatively healthy donors were collected in accordance with the ethical principles of the Declaration of Helsinki. The study protocol was reviewed and approved by the Medical Ethics Committee of the First Affiliated Hospital of Guangzhou Medical University (approval number: ES-2024-080-03). All procedures were conducted in compliance with relevant institutional and national ethical guidelines and regulations.

Immunofluorescence (IF) assay

The immunofluorescence (IF) assay was performed according to previously described methods. Lung tissue sections were incubated with specific antibodies, including *SERPING1* (A1717, ABclonal, China), FN1 (A23830, ABclonal, China), F4/80 (A23788, ABclonal, China), ECAD (A20798, ABclonal, China), C1R (A6360, ABclonal, China), C1s (A6878, ABclonal, China), C3 (A16781, ABclonal, China); PDGFRA (A2103, ABclonal, China) for 2 hours at 37°C. Following this, sections were treated with secondary antibodies: Alexa Fluor 488 goat anti-rabbit IgG (H+L) (1:500, Invitrogen) and Alexa Fluor 594 rabbit anti-mouse IgG (H+L) (1:500, Invitrogen) for 40 minutes. Images were captured using the Leica DM6 M microscope.

ELISA assay

Mouse IL-6 (EK206/3-96, Multi Sciences, China), MCP-1 (CCL2, EK287/2-96, Multi Sciences, China), IL-1β, TNF-α (EK182-96, Multi Sciences, China) and *SERPING1* (CSB-EL021086HU, CUSABIO, China) ELISA kits were utilized to assess the expression of IL-6, TNF-α and CCL2 in BALF, serum, lung tissue homogenates or cell supernatant, following the manufacturer's instructions. Human IL-6 (88-7066-88, ThermoFisher, USA), CCL2 (88-7399-88, ThermoFisher, USA) and TNF-α (88-7346-88, ThermoFisher, USA) were used to measure the secretion of IL-6, TNF-α and CCL2 in cell culture supernatants from HFL1 and THP-1 cells.

The lung tissue homogenates were prepared as previously described with diluted at a ratio of 1:9. Following lung function tests, lung tissues and whole blood were promptly collected from the mice. Their lungs were lavaged with two 0.5-ml instillations of ice-cold PBS, and serum was obtained by centrifuging whole blood at 2,000 g for 10 minutes at -4°C. The smallest lung lobe was excised and homogenized in 0.3 ml of ice-cold PBS. In the case of cell culture supernatant, it was collected by centrifugation at 2000×g for 10-15 minutes at room temperature.

Animals, COPD modeling and Lung function

All female C57/BL6 mice were obtained from Gempharmatech (Jiangsu), aged 7 weeks and weighing between 22 to 25 g. Lung-specific adeno-associated virus vector (AAV) encoding full-length *Serping1* (AAV-Serping1) and an empty control vector were obtained from Obio Technology (Shanghai, China). To evaluate the therapeutic effects of *Serping1*, mice were first exposed to cigarette smoke for 3 months to initiate COPD development. After this initial exposure, mice were anesthetized and intratracheally administered AAV at a dose of 2 × 10¹¹ genomic copies. Following a 2-week recovery period, the mice continued to receive cigarette smoke exposure for an additional 3 months. The chronic cigarette smoke-induced COPD model was established as previously described, with mice exposed to cigarette smoke for 2 hours in the morning and afternoon, 6 days per week, over a total duration of 6 months. After this exposure, mice were harvested for subsequent analyses. Ethical approval for the animal study was obtained from The First Affiliated Hospital of Guangzhou Medical University (Reference number: SYXK 2020-0227). Lung function in mice was assessed using a specialized invasive pulmonary function system, as previously described^22^. After calibrating the system, mice were anesthetized with an intraperitoneal injection of sodium pentobarbital at a dosage of 50 mg/kg, followed by a tracheostomy. The mice were then placed in the DSI Buxco Pulmonary Function Test (PFT) apparatus (Buxco Research Systems, USA) for further evaluation. A range of measurements were taken, including lung chord compliance (Cchord), dynamic compliance (Cdyn), functional residual capacity (FRC), airway resistance (RI), and forced expiratory volume at 50 milliseconds (FEV_50_).

Hematoxylin-Eosin (HE), Masson, and Immunohistochemistry (IHC) staining

HE and Masson staining techniques were conducted as previously described^25^. In brief, tissue samples were embedded in paraffin, deparaffinized, and rehydrated before undergoing HE, and Masson staining. The mean linear intercept (MLI) was determined to estimate the average diameter of an individual alveolus, using the formula: MLI = total length / number of alveolar septa. To evaluate airway wall thickness, the segmental airway wall area percentage was calculated, defined by the equation: Segmental WA% = [outer bronchus area - airway luminal area] / outer bronchus area. Airway area and the Masson-positive area were measured with ImageJ software, enabling the calculation of the percentage of stained area in relation to the total airway wall area (collagen area / airway area).


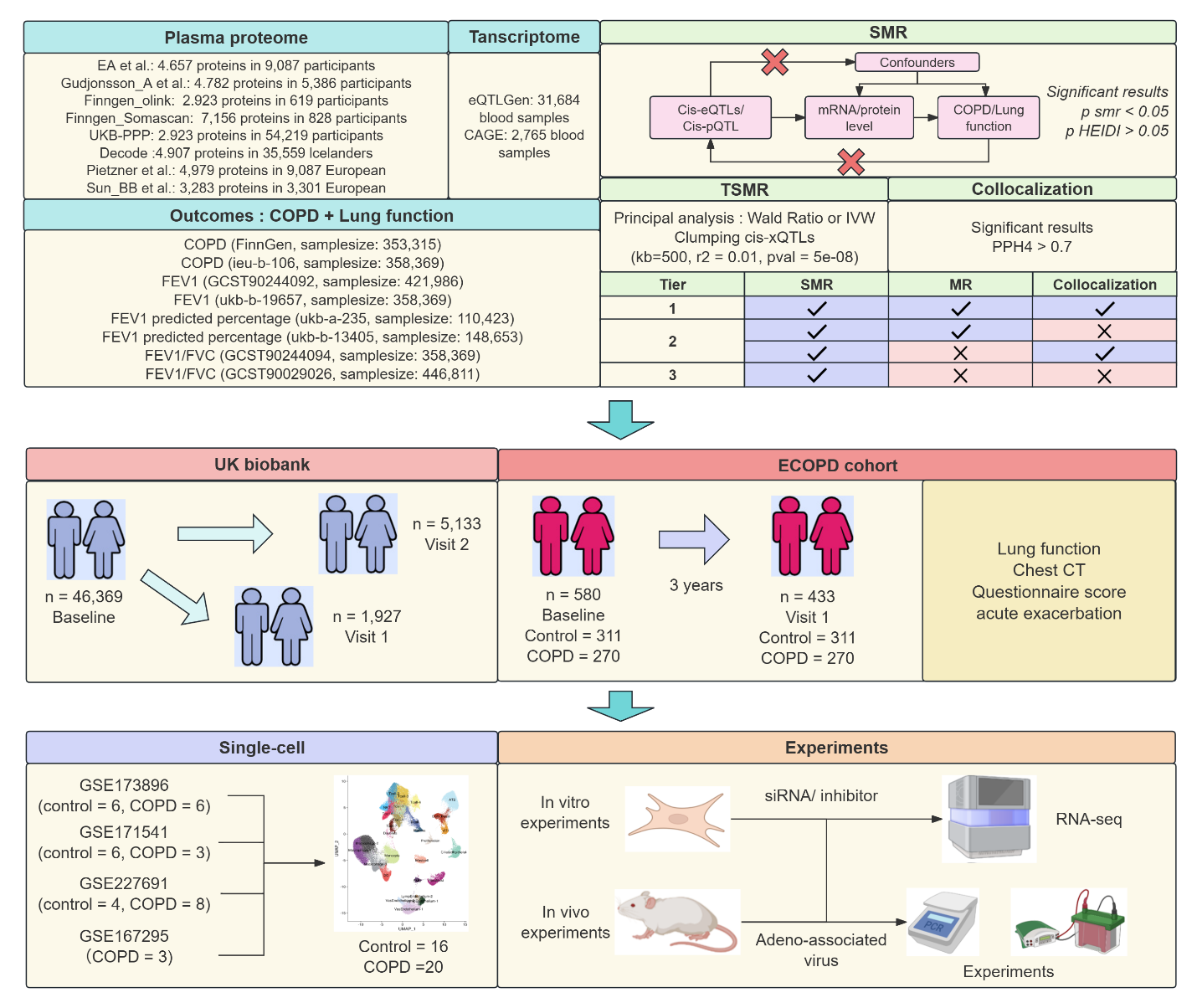


Figure. S1.

The schematic workflow of the study was created with *BioRender.com*.


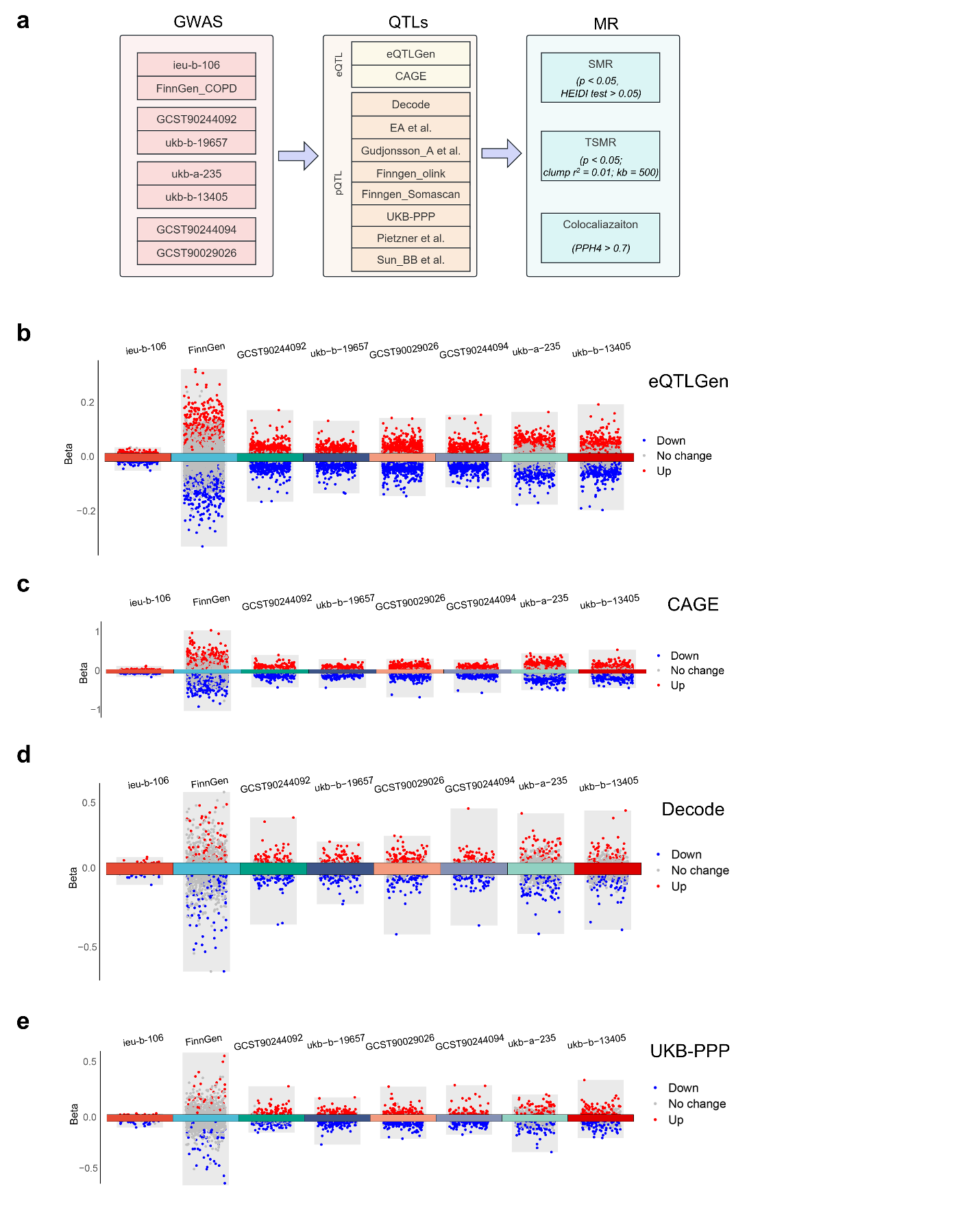


Figure. S2.

**a** four-stage causal prioritization framework: (1) Integration of genome-wide association study (GWAS) data for COPD risk, FEV_1_, FEV_1_% predicted, and FEV_1_/FVC ratio incorporating discovery and replication cohorts; (2) Summary-data-based Mendelian Randomization (SMR) analysis leveraging two expression quantitative trait loci (eQTL) repositories and eight plasma protein QTL (pQTL) datasets; (3) Two-sample MR validation of SMR-significant genes using inverse-variance weighted and MR-Egger methods; (4) Bayesian colocalization analysis (*PPH4 >0.8*) to confirm shared causal variants between molecular traits and pulmonary outcomes. **b-e** Volcano plots illustrating SMR associations between COPD-related phenotypes (COPD risk, FEV_1_, FEV_1_% predicted, FEV_1_/FVC ratio) and molecular QTLs from four databases: eQTLGen (**b**), CAGE (**c**), Decode (**d**), and UKB-PPP(**e**). Red points denote significant positive associations; blue points indicate significant negative associations. Y-axis represents effect size magnitude (|β|).


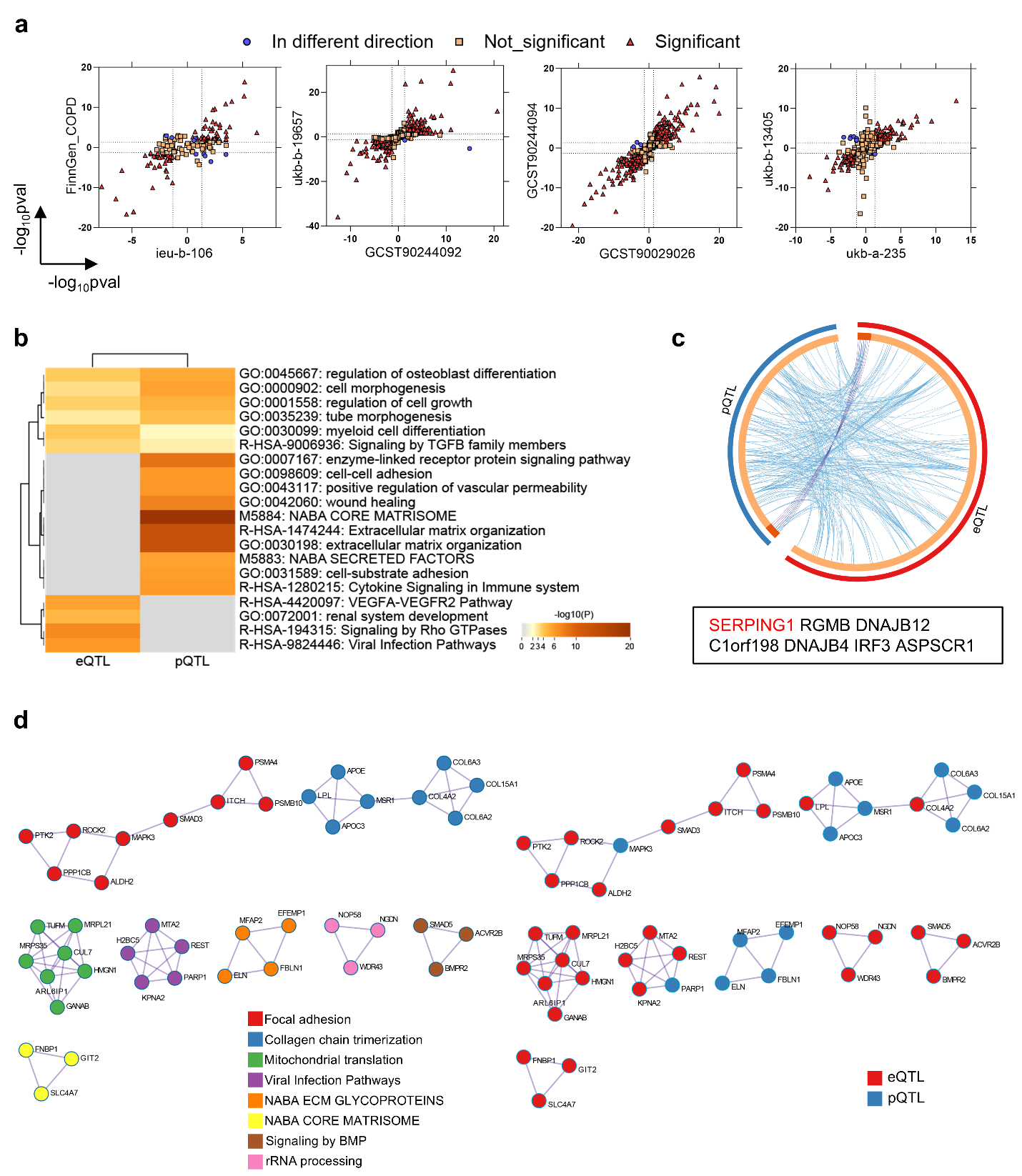


Figure. S3.

**a** Volcano plots demonstrate cross-cohort concordance of transcriptome-wide SMR associations for COPD-related phenotypes across four GWAS datasets: (**1**) FinnGen COPD (x-axis: ieu-b-106) vs. UK Biobank COPD (y-axis: ukb-b-19657), (**2**) FEV_1_:GCST90244094 vs ukb-b-13405, (**3**) FEV_1_/FVC: GCST90244092 vs. GCST90244092, and (**4**) FEV_1_% predicted: ukb-a-235 vs ukb-b-13045. Data points are color/symbol-coded: red triangles (*p < 0.05* in both cohorts with concordant beta directions), blue circles (*p < 0.05* in both cohorts with discordant directions), and brown squares (non-significant in either cohort). Axes display *-log^10^-transformed p-values* with genome-wide significance thresholds marked by horizontal/vertical dashed lines. **b** The Circos plot demonstrates overlapping genes showing significant positive or negative correlations with Tier 1 evidence levels for both pQTL and eQTL. Purple connecting lines depict identical genes, while blue lines represent genes with functional similarity or homology. **c** Combined enrichment analysis of significant genes with Tier 1 evidence levels**. d** Protein-protein interaction (PPI) network of Tier 1 eQTL and pQTL genes, highlighting enriched functional pathways.​


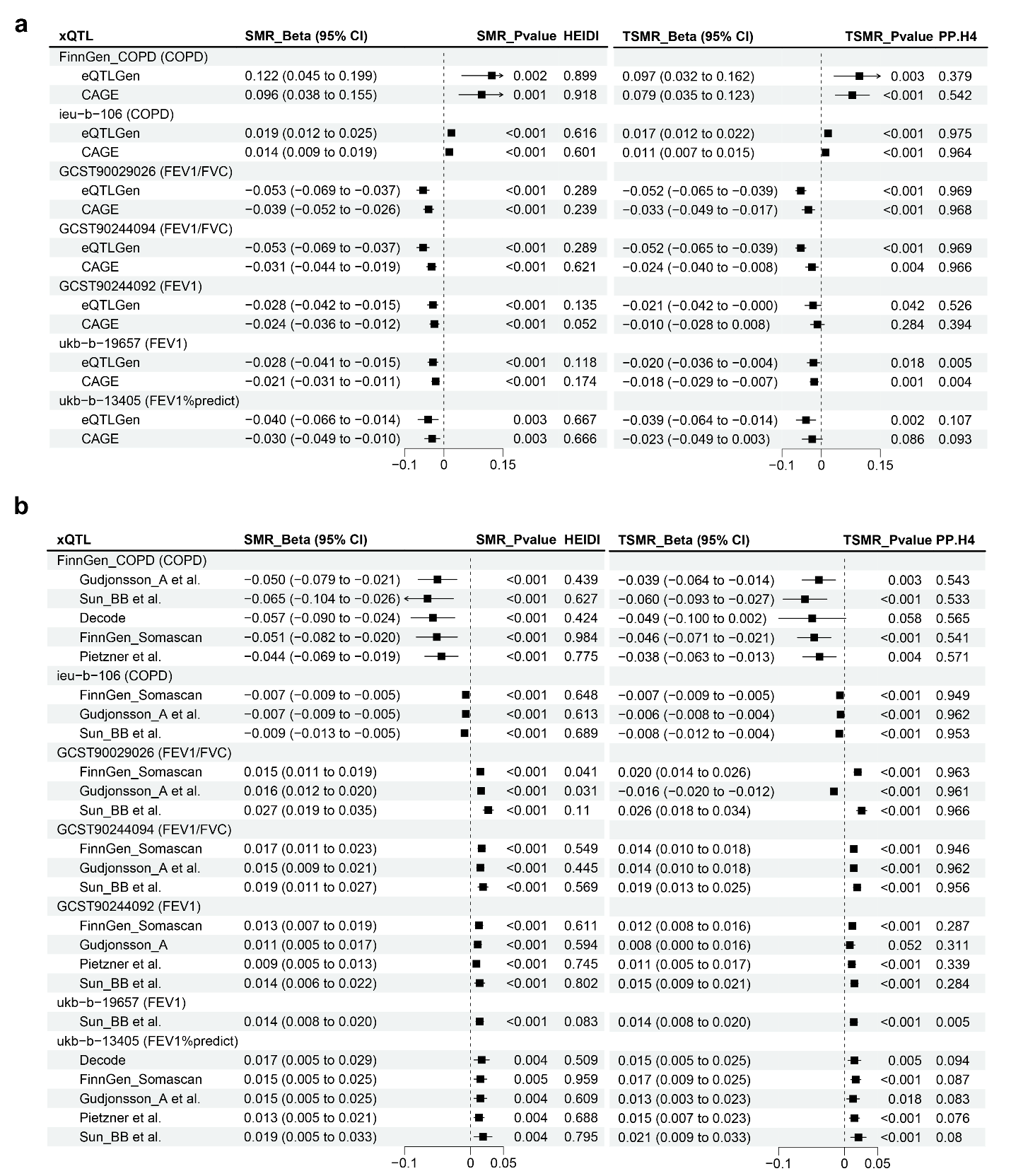


**Figure. S4.**

**a** The Forest plot shows *SERPING1* eQTL associations with COPD risk and lung function traits (FEV_1_, FEV_1_% predicted, FEV_1_/FVC with replication cohorts) via two-sample SMR analyses, SMR and Bayesian colocalization analyses. **b** The Forest plot shows *SERPING1* pQTL associations with COPD risk and lung function traits (FEV_1_, FEV_1_% predicted, FEV_1_/FVC with replication cohorts) via two-sample SMR analyses, SMR and Bayesian colocalization analyses.


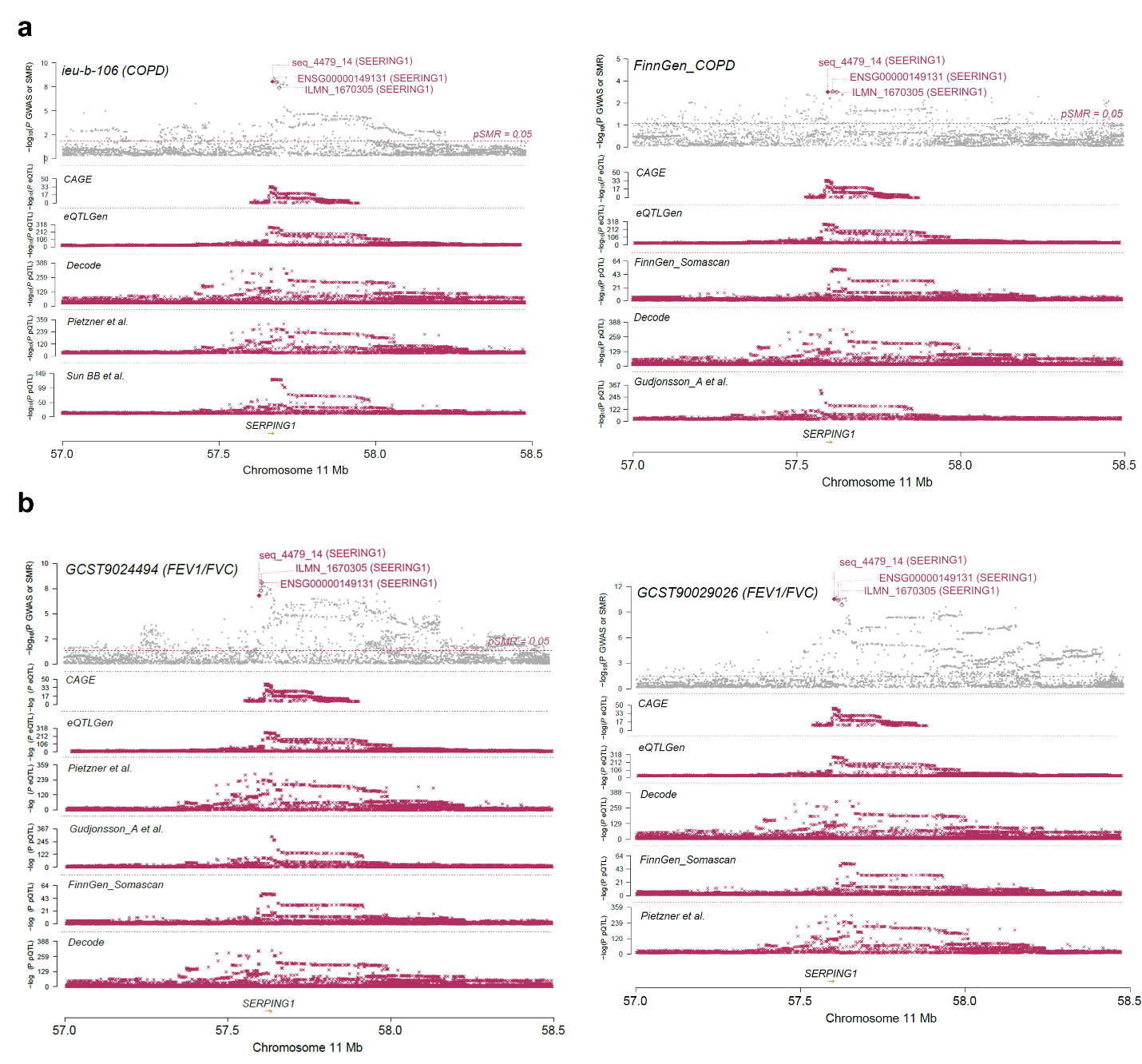


**Figure. S5.**

Manhattan plot displays chromosome 11 (57.0-58.5 Mb) associations with COPD including ieu-b-106 and FinnGen_COPD (**a**) or GCST9024494 and GCST90029026 (**b**) through GWAS and SMR analyses. Three loci [seq_4479_14 (pQTL), ENSG00000149131 (eQTL), ILMN_1670305 (mQTL)] exhibit genome-wide significant signals (*p_SMR < 0.05*) annotated to *SERPING1*. Multi-omics concordance across the datasets (xQTLs) is demonstrated as visualized in Figure​.


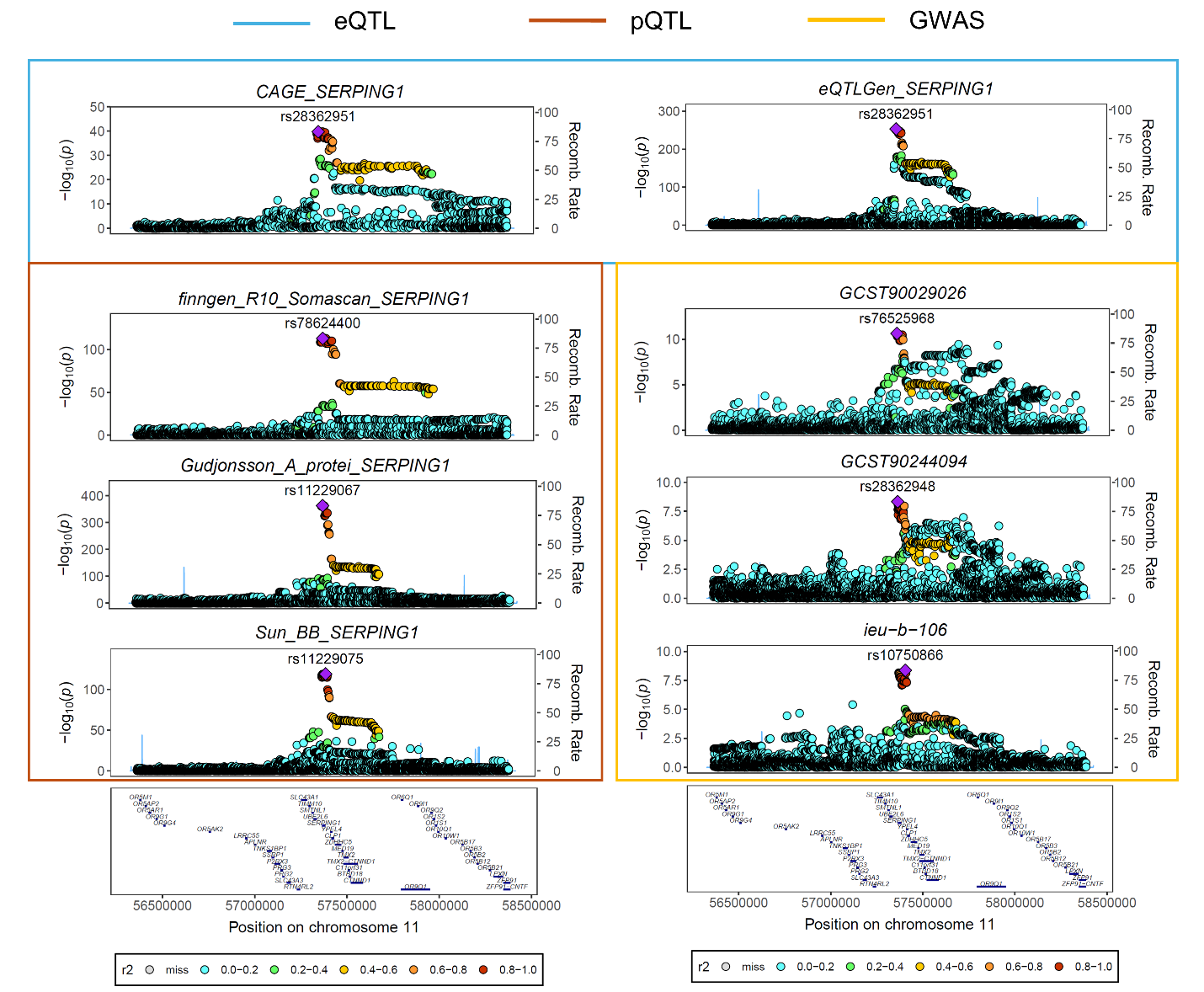


**Figure. S6.**

This integrative plot demonstrates multi-omics colocalization of *SERPING1* at chr11:57.0-58.5 Mb (hg37). Left panels illustrate cis-regulatory effects on transcripts (blue, eQTL) and proteins (brown, pQTL) across five cohorts: CAGE, eQTLGen, FinnGen_Somascan, Gudjonsson_A et al., and Sun_BB et al. Right panels map genome-wide significant COPD associations (yellow, GWAS) from three studies (GCST90029026, GCST90244094, ieu-b-106), highlighting key SNPs: rs28362951 (eQTL), rs78624400 (pQTL) and rs11229075 (pQTL). Color gradients on data points reflect linkage disequilibrium strength (LD; *r²*: blue=0 → red=1) relative to the index variant. The x-axis spans genomic coordinates (GRCh37, chr11:57,362,951–57,428,067), while the y-axis quantifies association significance as −log₁₀(*p*) for single-nucleotide variants (SNVs).


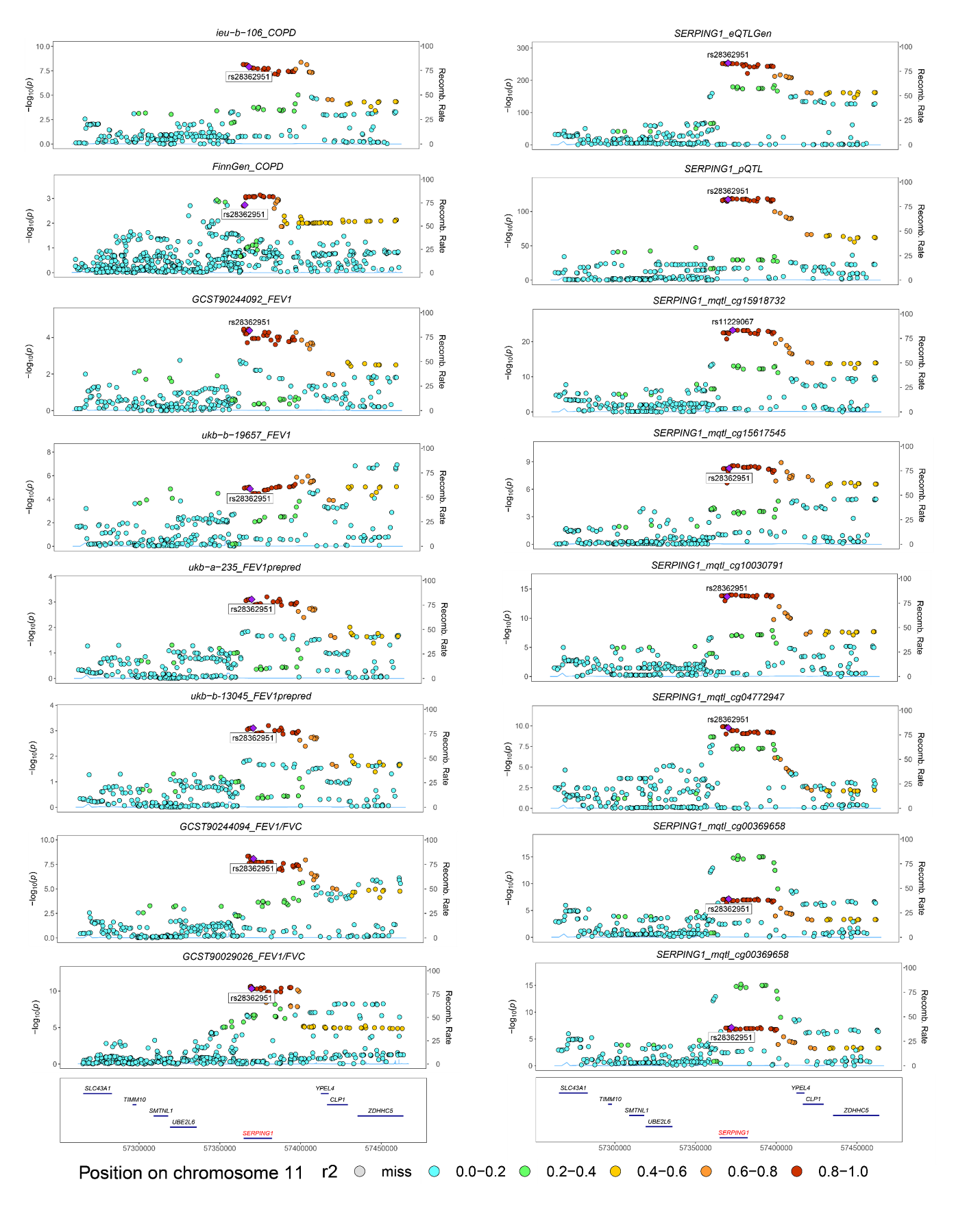


**Figure. S7.**

Manhattan plot presenting co-localization analysis via​Hyprcoloc​of multi-omics QTLs—including eQTLs (RNA expression), pQTLs (protein expression), and mQTLs (methylation)—with COPD risk and lung function parameters across independent cohorts. Colors denote LD correlation coefficients (r2): ​light blue​ (0–0.2), ​green​ (0.2–0.4), ​yellow​ (0.4–0.6), ​orange​ (0.6–0.8), ​red​ (0.8–1.0). ​Potential driver SNPs​ (annotated rsIDs) are highlighted, and protein-coding genes in the chr11:57.0–58.5 Mb region are mapped below.


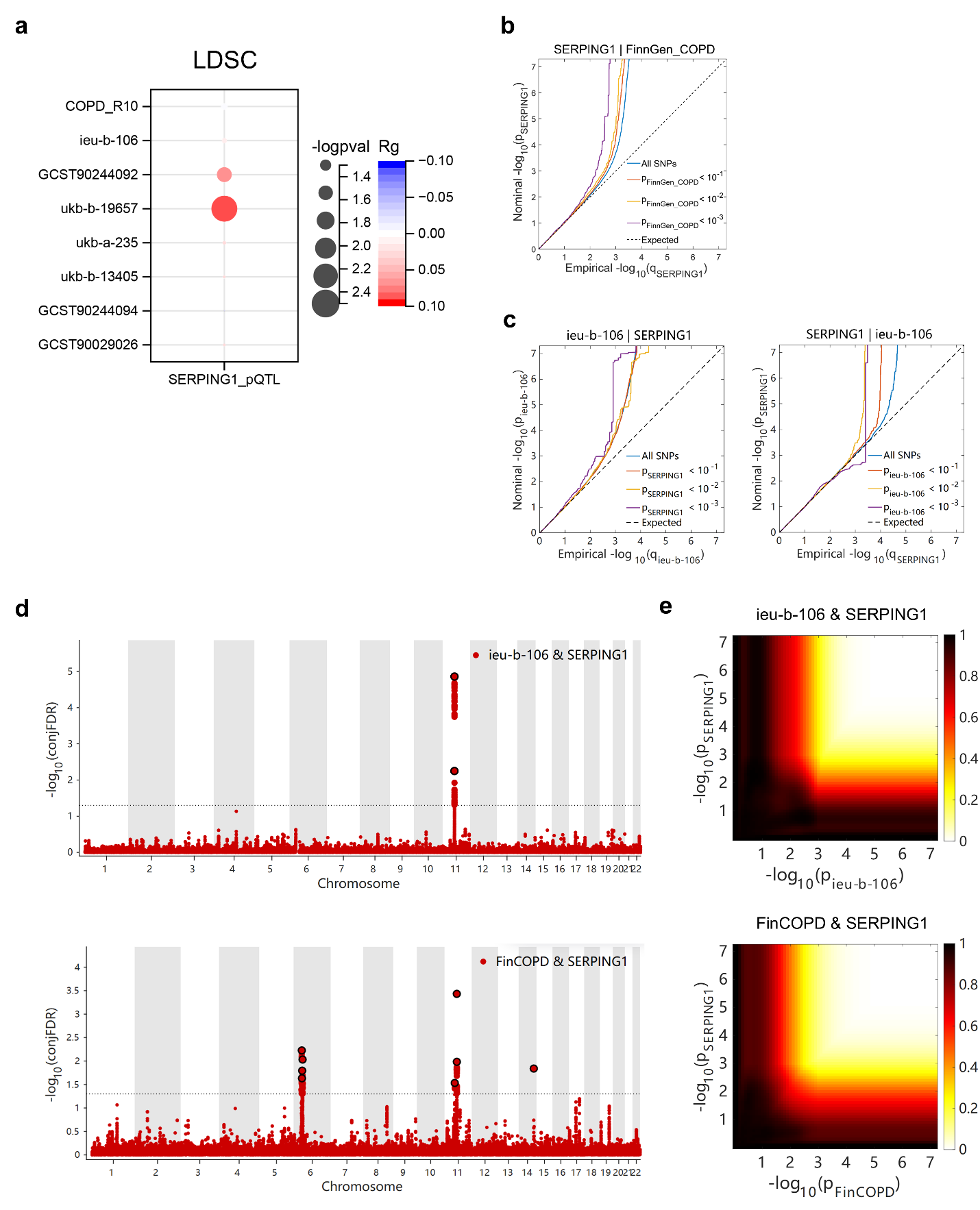


**Figure. S8.**

**a** This LD score regression (LDSC) plot illustrates genetic correlations between *SERPING1* pQTL and COPD-related phenotypes (COPD risk, FEV_1_, FEV_1_% predicted, FEV_1_/FVC with replication cohorts). Point size encodes association significance, while color gradients reflect genetic correlation coefficients. Polygenic overlap between *SERPING1* pQTL and COPD including ieu-b-106 (**b**) and FinnGen_COPD (**c**). Conditional Q-Q plots of nominal versus empirical -log10(*p*) values (corrected for inflation) for the primary trait (*SERPING1* pQTL) are presented below the standard GWAS threshold of *p < 5 x 10⁻⁸*, as a function of the significance of association with the secondary trait at the levels of *p ≤ 0.1, p ≤ 0.01*, and *p ≤ 0.001*, respectively. Blue line: all SNPs; dashed line: null hypothesis. **d** Manhattan plot provides the association results for single marker analysis of *SERPING1* pQTL and COPD (ieu-b-106 and FinnGen_COPD). The independent significant lead SNPs are encircled in black. **e** Heatmap showing the FDR for pleiotropic signals between *SERPING1* pQTL and COPD (ieu-b-106 and FinnGen_COPD). *-Log10(p) values* for COPD are on the x-axis, and for *SERPING1* pQTL on the y-axis. Color intensity represents *FDR* values, ranging from black (high *FDR*, low pleiotropic probability) to white (low *FDR*, high pleiotropic probability). SNPs with high significance in both traits (top right corner) exhibit lower *FDR*, suggesting potential pleiotropic signals.


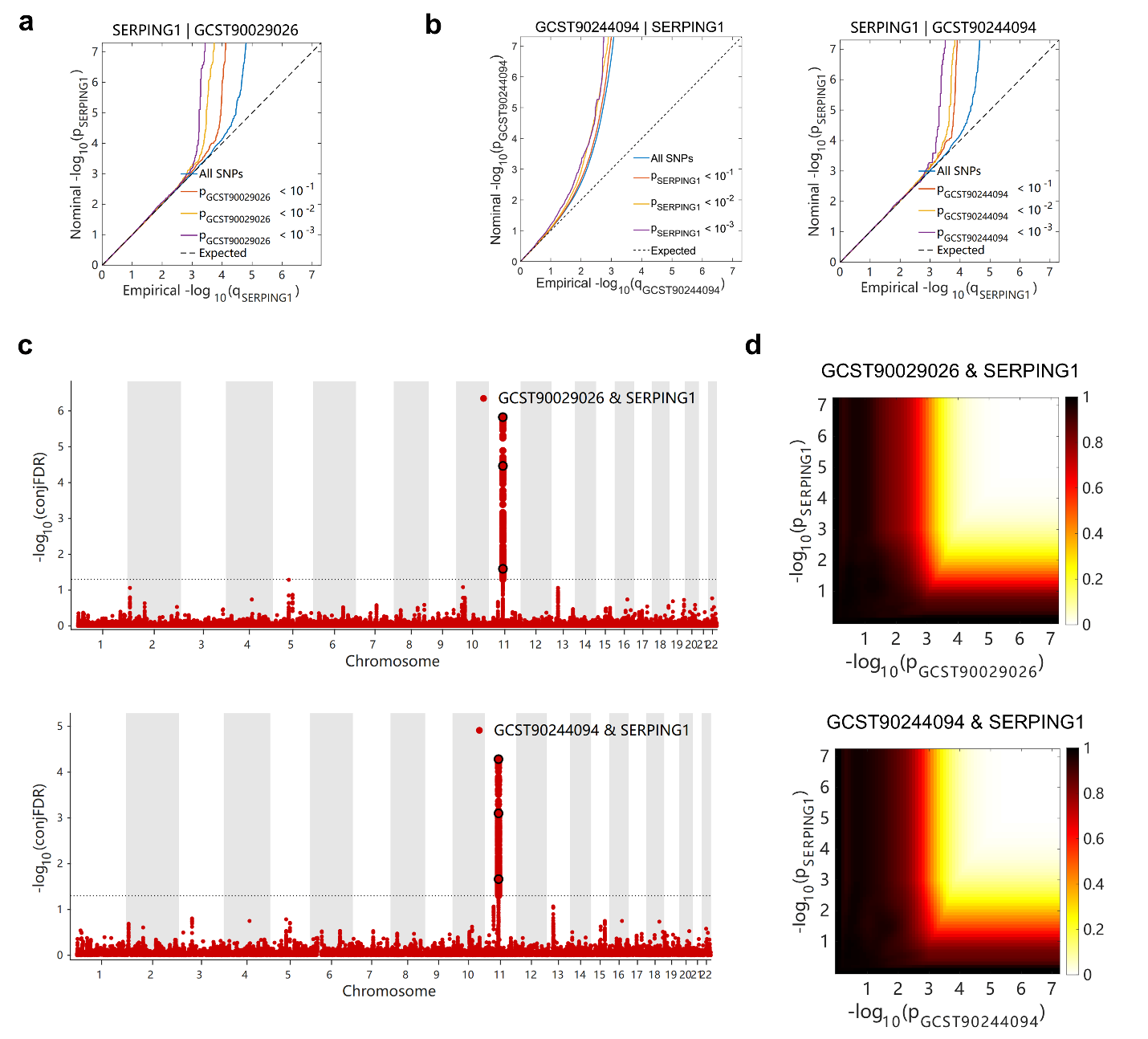


**Figure. S9.**

Polygenic overlap between *SERPING1* pQTL and FEV_1_/FVC in GCST90244094 (**a**) and GCST90029026 (**b**) cohort. Conditional Q-Q plots of nominal versus empirical -log10(p) values (corrected for inflation) for the primary trait (*SERPING1* pQTL) are presented below the standard GWAS threshold of *p < 5 x 10⁻⁸*, as a function of the significance of association with the secondary trait at the levels of *p ≤ 0.1, p ≤ 0.01*, and *p ≤ 0.001*, respectively. Blue line: all SNPs; dashed line: null hypothesis. Manhattan plot provides the association results for single marker analysis of *SERPING1* pQTL and FEV_1_/FVC including GCST90244094 and GCST90029026 (**c**). The independent significant lead SNPs are encircled in black. **d** Heatmap showing the FDR for pleiotropic signals between *SERPING1* pQTL and COPD (GCST90244094 and GCST90029026). *-Log10(p) values* for COPD are on the x-axis, and for *SERPING1* pQTL on the y-axis. Color intensity represents *FDR* values, ranging from black (high *FDR*, low pleiotropic probability) to white (low *FDR*, high pleiotropic probability). SNPs with high significance in both traits (top right corner) exhibit lower *FDR*, suggesting potential pleiotropic signals.


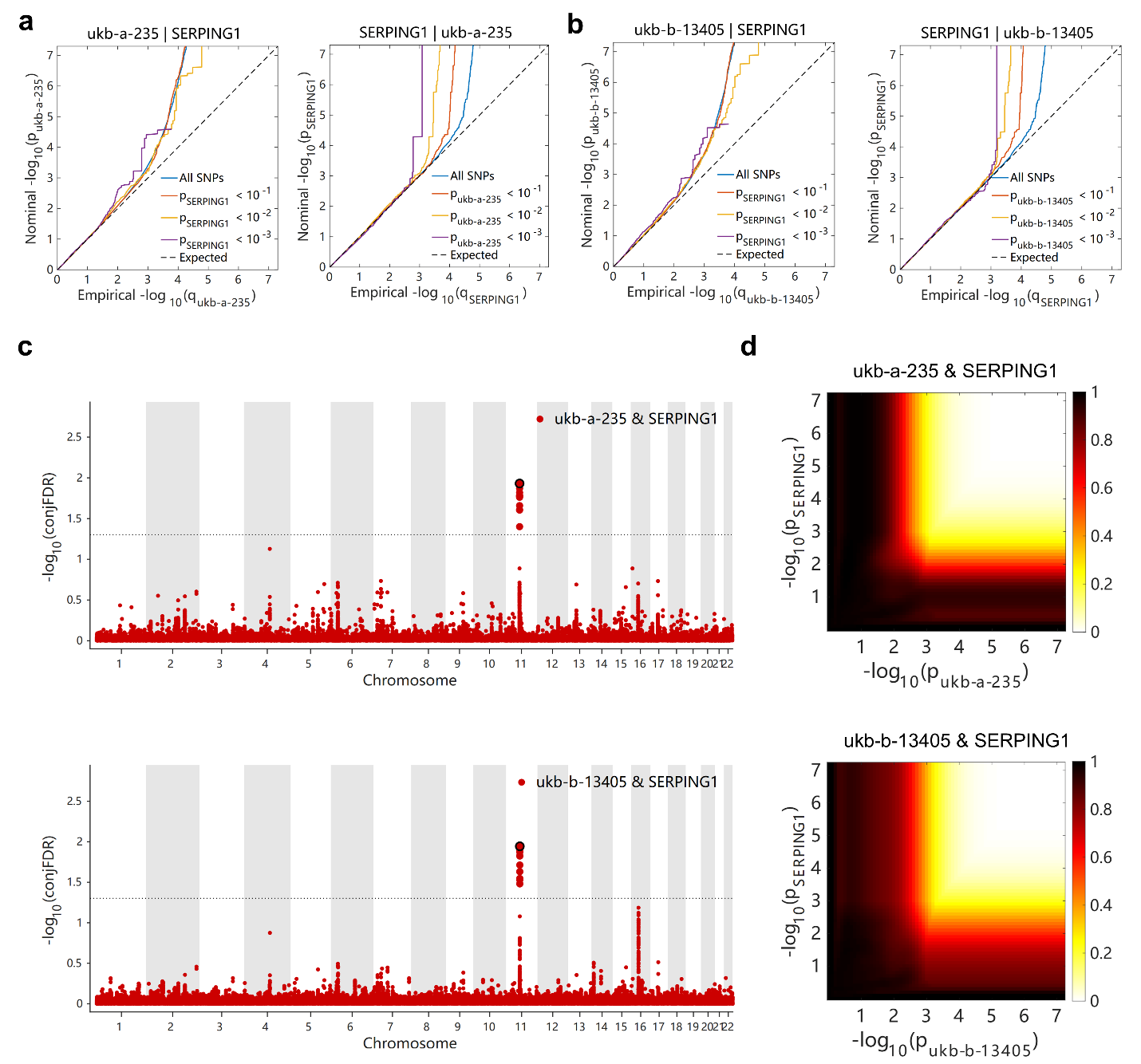


**Figure. S10.**

Polygenic overlap analysis of *SERPING1* pQTL and FEV₁% predicted in ukb-b-235 (**a**) and ukb-b-13405 (**b**) cohorts. Conditional Q-Q plots of nominal versus empirical -log10(*p*) values (corrected for inflation) for the primary trait (SERPING1 pQTL) are presented below the standard GWAS threshold of *p < 5 x 10⁻⁸*, as a function of the significance of association with the secondary trait at the levels of *p ≤ 0.1, p ≤ 0.01*, and *p ≤ 0.001*, respectively. Blue line: all SNPs; dashed line: null hypothesis. Manhattan plot provides the association results for single marker analysis of *SERPING1* pQTL and FEV_1_/FVC including ukb-b-235 and ukb-13405 (**c**)**​**, with independent significant lead SNPs encircled in black. **d** Heatmap showing the FDR for pleiotropic signals between *SERPING1* pQTL and COPD (ukb-b-235 and ukb-13405). *-Log10(p) values* for COPD are on the x-axis, and for SERPING1 pQTL on the y-axis. Color intensity represents *FDR* values, ranging from black (high *FDR*, low pleiotropic probability) to white (low *FDR*, high pleiotropic probability). SNPs with high significance in both traits (top right corner) exhibit lower *FDR*, suggesting potential pleiotropic signals.


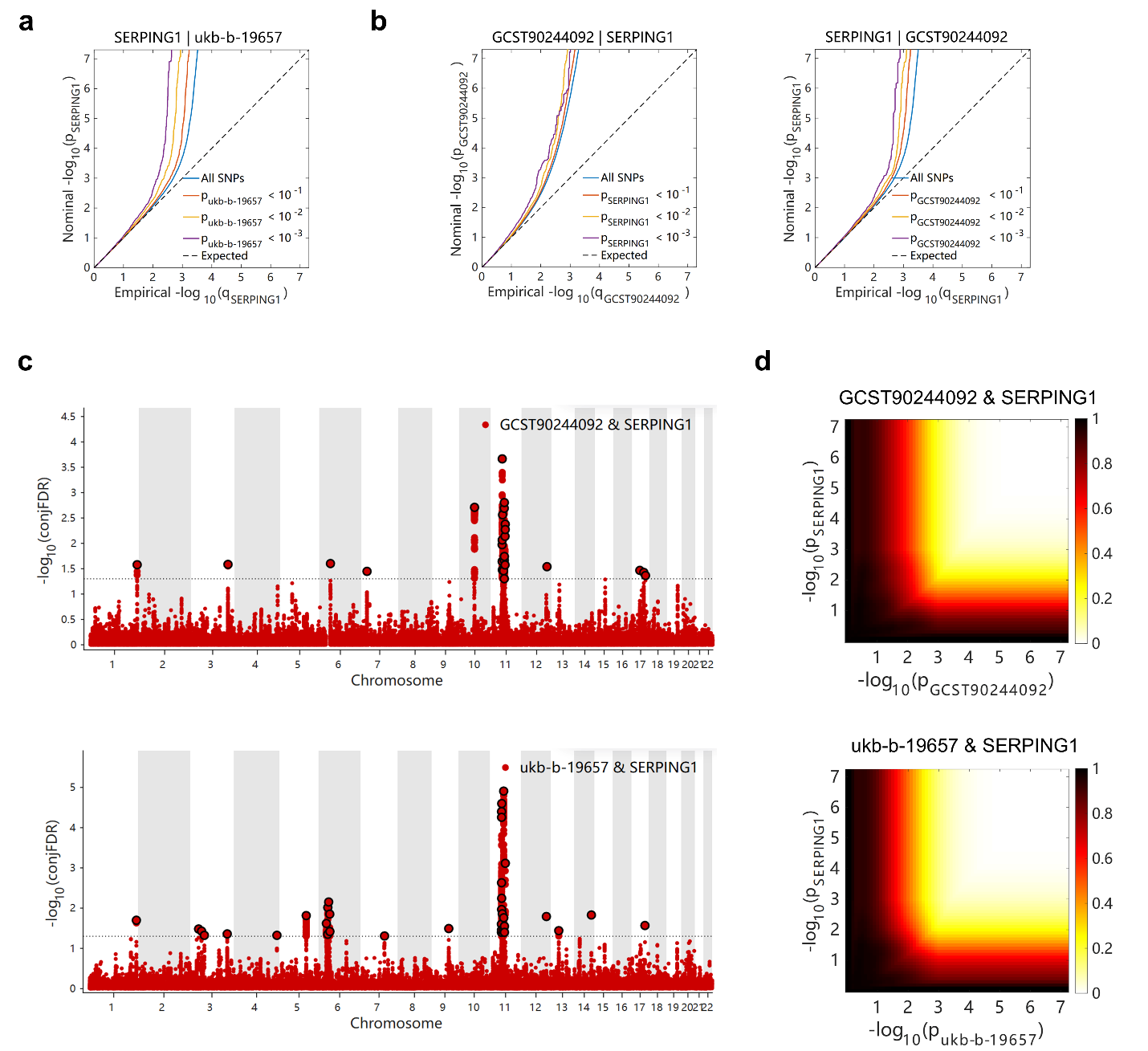


**Figure. S11.**

Polygenic overlap between SERPING1 pQTL and FEV_1_ including ukb-b-19657 (**a**) and GCST90244092 (**b**). Conditional Q-Q plots of nominal versus empirical -log10(p) values (corrected for inflation) for the primary trait (SERPING1 pQTL) are presented below the standard GWAS threshold of *p < 5 x 10⁻⁸*, as a function of the significance of association with the secondary trait at the levels of *p ≤ 0.1, p ≤ 0.01*, and *p ≤ 0.001*, respectively. Blue line: all SNPs; dashed line: null hypothesis. **c** Manhattan plot provides the association results for single marker analysis of *SERPING1* pQTL and FEV_1_ including GCST90244092 and ukb-b-19657. The independent significant lead SNPs are encircled in black. **d** Heatmap showing the FDR for pleiotropic signals between *SERPING1* pQTL and COPD (GCST90244092 and ukb-b-19657). *-Log10(p) values* for COPD are on the x-axis, and for *SERPING1* pQTL on the y-axis. Color intensity represents *FDR* values, ranging from black (high *FDR*, low pleiotropic probability) to white (low *FDR*, high pleiotropic probability). SNPs with high significance in both traits (top right corner) exhibit lower FDR, suggesting potential pleiotropic signals.


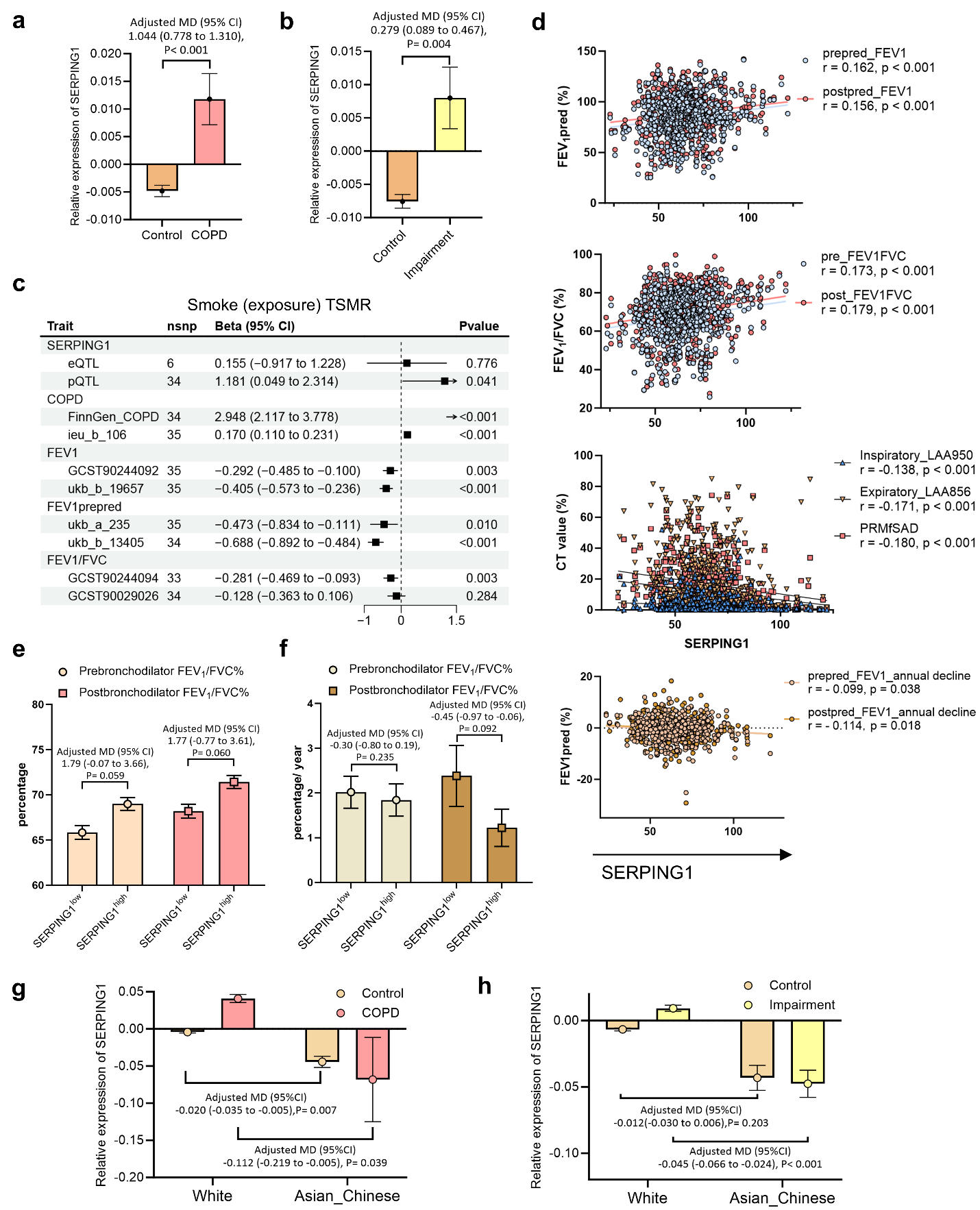


**Figure. S12.**

The bar chart shows adjusted differences in *SERPING1* expression (measured by Olink assay) between the Control group versus COPD (**a**) and Control versus Impaired Lung Function (**b**) in the UK Biobank cohort. Analyses were adjusted for age, sex, BMI, race, smoking status, alcohol intake, and income. Error bars represent the standard error of the mean (SEM). **c** Forest plot summarizing two-sample Mendelian randomization (TSMR) analyses the causal effects of smoking exposure on *SERPING1* regulation (via eQTL/pQTL instruments) and lung function. Effect estimates were derived using inverse-variance weighted (IVW) regression. **d** Correlation analysis using Pearson's method​between *SERPING1* levels (measured by ELISA) and baseline pulmonary function parameters (FEV₁% predicted, ​FEV₁/FVC ratio), CT-derived metrics (Inspiratory LAA₉₅₀, Expiratory LAA₈₅₆, and ​PRMᶠˢᵃᴰ), and the annual decline rate of FEV₁% predicted in the ECOPD cohort. Bar chart showing adjusted differences in (**e**) pre-/post-bronchodilator FEV₁/FVC ratios and (**f**) annual decline rates of FEV₁/FVC ratios between high- and low-*SERPING1* expression groups in the ECOPD cohort. Groups were stratified by median serum *SERPING1* levels (measured by ELISA) and analyses were adjusted for age, sex, BMI, smoking history (status and pack-year index). Error bars represent SEM. Bar chart depicting adjusted differences in serum *SERPING1* expression between ethnic groups (White, Asian and Chinese), stratified by Control vs. COPD status (**g**) and Control vs. Impaired Lung Function (**h**) in the UK Biobank cohort. Analyses adjusted for age, sex, BMI, smoking status, alcohol intake, and household income; error bars represent standard SEM.


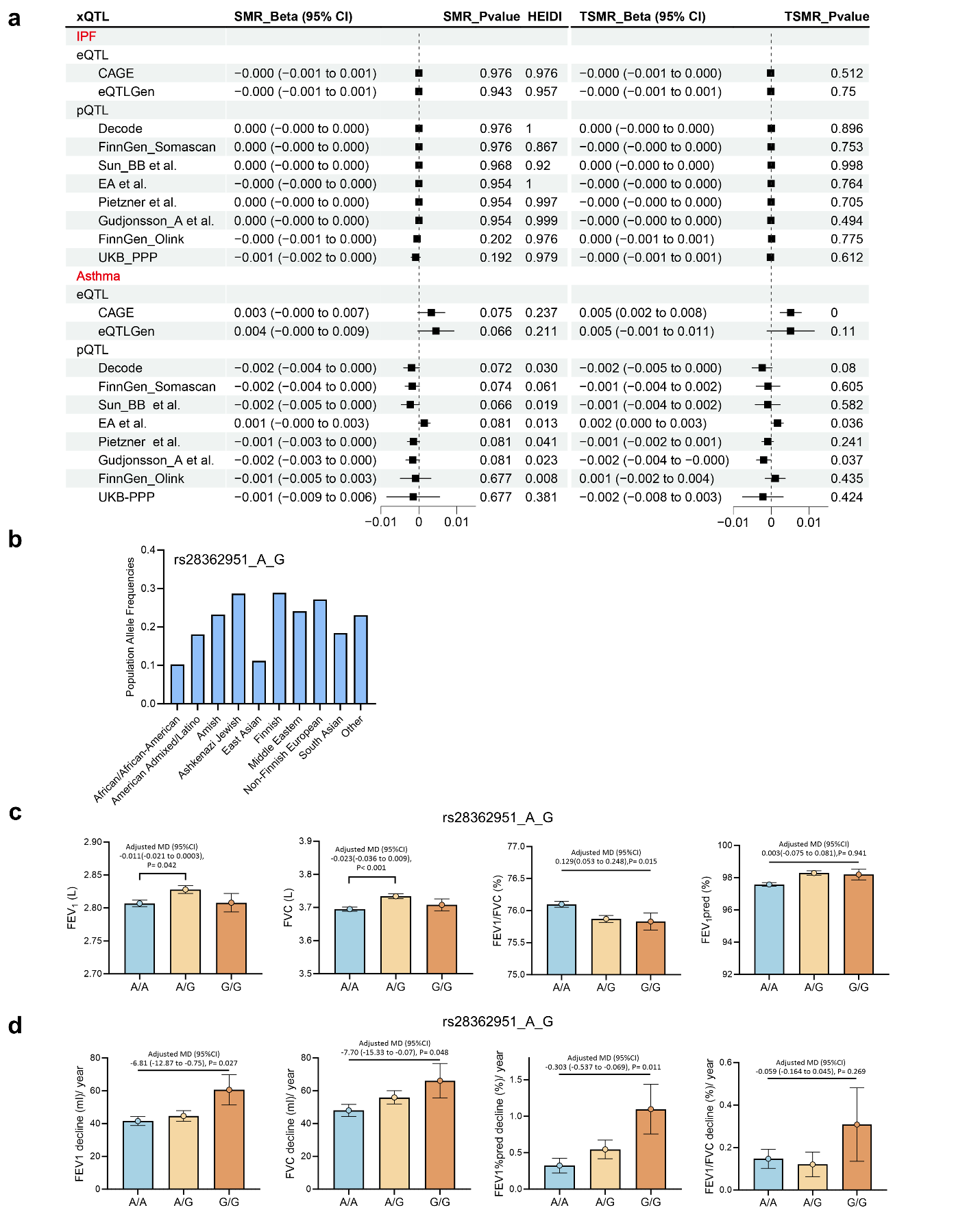


**Figure. S13.**

**a** Forest plot summarizing Mendelian randomization analyses of the causal effects of *SERPING1* regulation (via eQTL/pQTL instruments) on idiopathic pulmonary fibrosis (IPF) and asthma. Effect estimates include: SMR and TSMR with IVW. **b** Population-specific allele frequency of the rs28362951 variant in the gnomAD database (v4.1.0).​ Bar chart showing adjusted differences in baseline lung function parameters: FEV₁, FVC, FEV₁/FVC ratio, and FEV₁% predicted (**c**), and their annual decline rates at Visit 1 (**d**) among rs28362951 genotype groups (wild-type, heterozygous, homozygous variant) within the UK Biobank cohort. Analyses were adjusted for age, sex, BMI, race, smoking status, alcohol intake frequency, and household income. Error bars represent SEM.


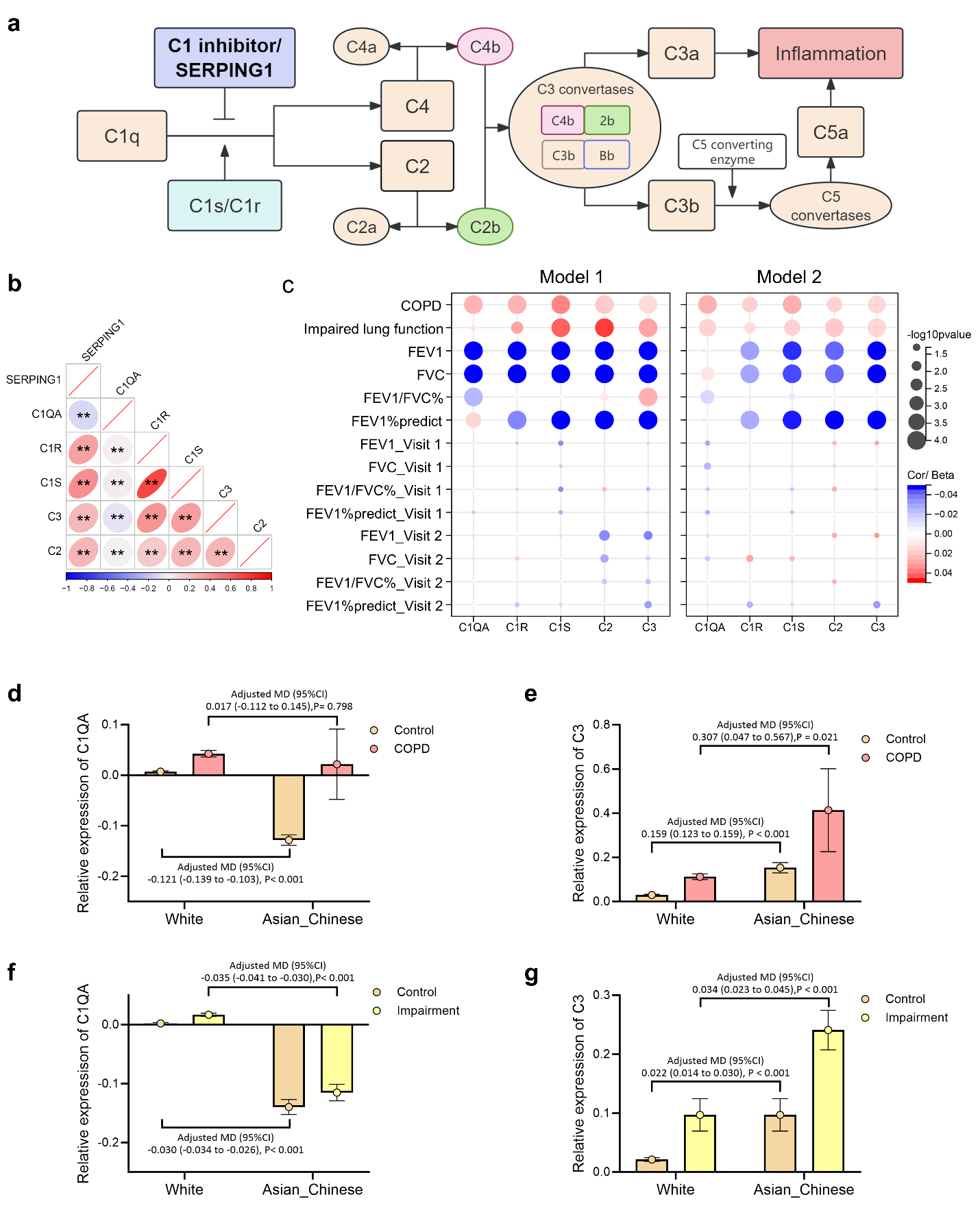


**Figure. S14.**

**a** The schematic illustrates the complement activation pathways. **b** Dot plot of associations between complement proteins (C1r, C1s, C1QA, C1QBP, C2, C3, C5) and lung function metrics in the UK Biobank cohort: baseline parameters (FEV₁, FVC, FEV₁/FVC ratio, FEV₁% predicted) and annual decline rates at Visit 1 and Visit 2 (FEV₁, FVC, FEV₁/FVC ratio, FEV₁% predicted), analyzed by univariate regression (Model 1, unadjusted) and multivariable regression (Model 2, adjusted for age, sex, BMI, and smoking status); red circles indicate positive associations, blue circles negative associations, with circle size proportional to -log₁₀(p-value) (larger circles denote stronger statistical significance). **c** Correlation analysis of *SERPING1* expression with complement cascade components (C1QA, C1R, C1S, C2, and C3) in the UKB cohort. Bar chart showing adjusted differences in serum C1QA (**d**) and C3 (**e**) expression between Control versus COPD, stratified by White, Asian and Chinese ethnic groups within the UK Biobank cohort. Analyses were adjusted for age, sex, BMI, smoking status, alcohol intake frequency, and income. Error bars represent SEM. Bar chart showing adjusted differences in serum C1QA (**f**) and C3 (**g**) expression between Control versus Impaired Lung Function, stratified by White, Asian and Chinese ethnic groups within the UK Biobank cohort. Analyses were adjusted for age, sex, BMI, smoking status, alcohol intake frequency, and income. Error bars represent SEM.


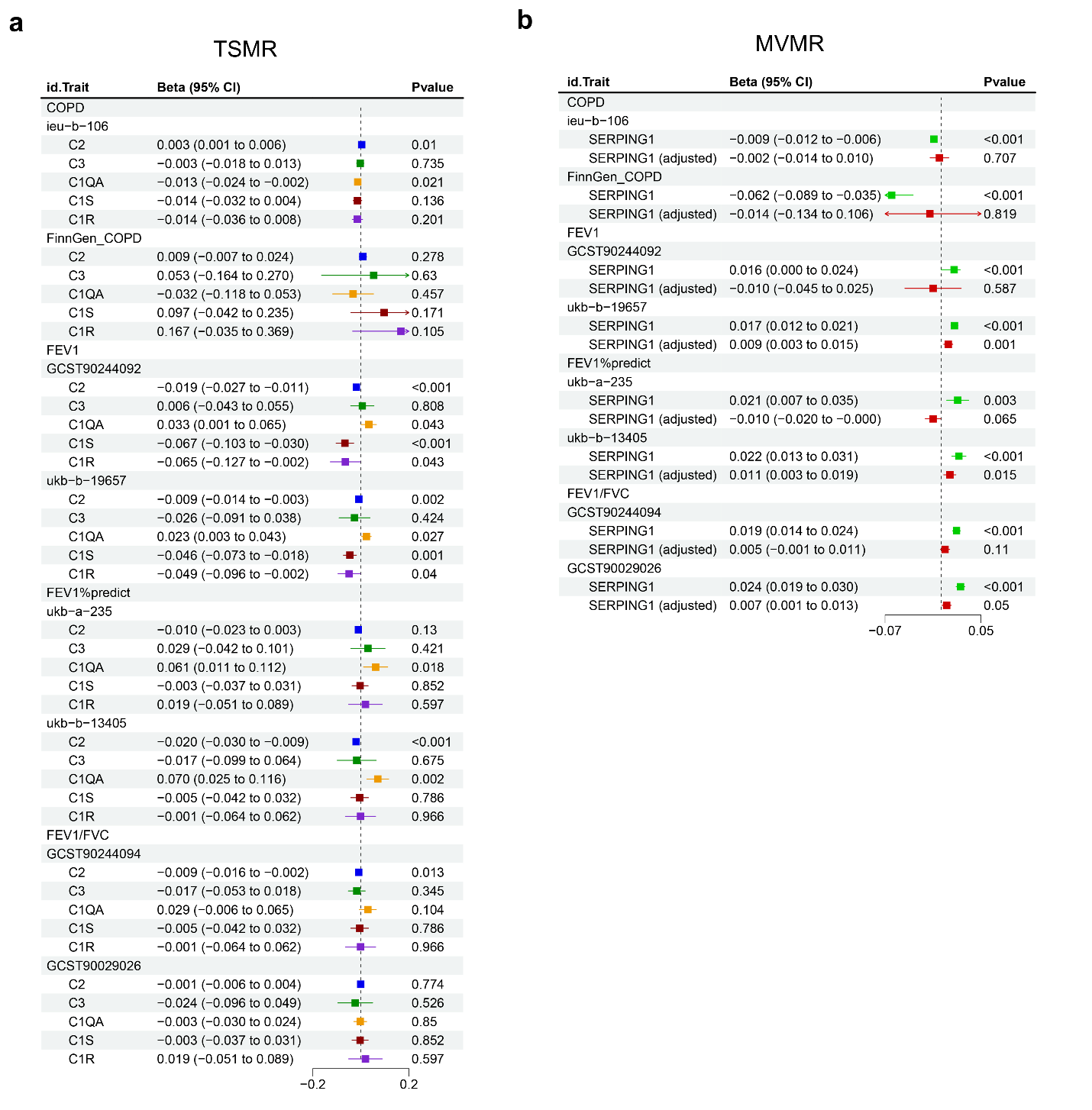


**Figure. S15.**

**a** Forest plot displaying TSMR results for pQTLs of complement proteins (C2, C3, C1QA, C1S, C1R) on COPD risk and lung function metrics (FEV₁, FVC, FEV₁/FVC ratio, FEV₁% predicted). **b** Forest plot presenting TSMR results for *SERPING1* pQTLs, alongside multivariable MR (MVMR) analyses adjusting for pleiotropic effects of complement system proteins (C1QA, C2, and C3 pQTLs incorporated as covariates in the exposure model).


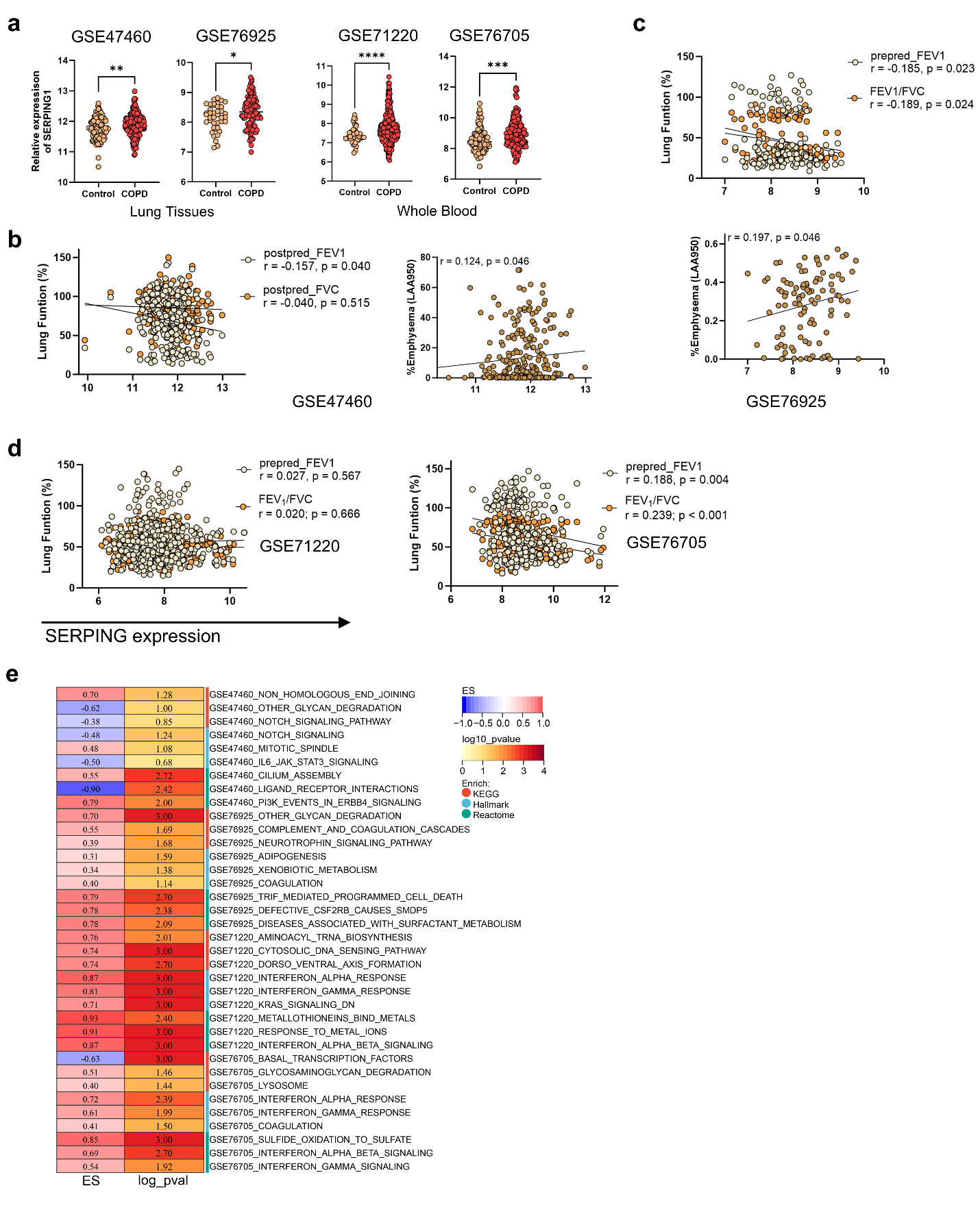


**Figure. S16.**

**a** Expression levels of *SERPING1* in lung tissues from COPD patients (GSE47460, GSE76925) and peripheral blood (GSE71220, GSE76705) are shown as mean ± standard deviation (SD). Correlation analyses between *SERPING1* expression levels and pulmonary function parameters (**b**) and emphysema index (%LAA950) (**c**) in Lung COPD cohorts from GEO datasets GSE47460 and GSE76925. **d** Correlation analyses between *SERPING1* expression levels and pulmonary function parameters in blood COPD cohorts from GEO datasets GSE71220 and GSE76705. **e** Heatmap visualization of Gene Set Enrichment Analysis (GSEA) results comparing *SERPING1* high-expression versus low-expression groups across lung tissues from COPD patients (GSE47460, GSE76925) and peripheral blood samples (GSE71220, GSE76705). Enrichment Score (ES) quantifies pathway activation magnitude. Statistical significance between groups was determined by two-tailed Student’s *t*-tests (**a**), and correlation significance assessed via Pearson analysis (**b-d**).


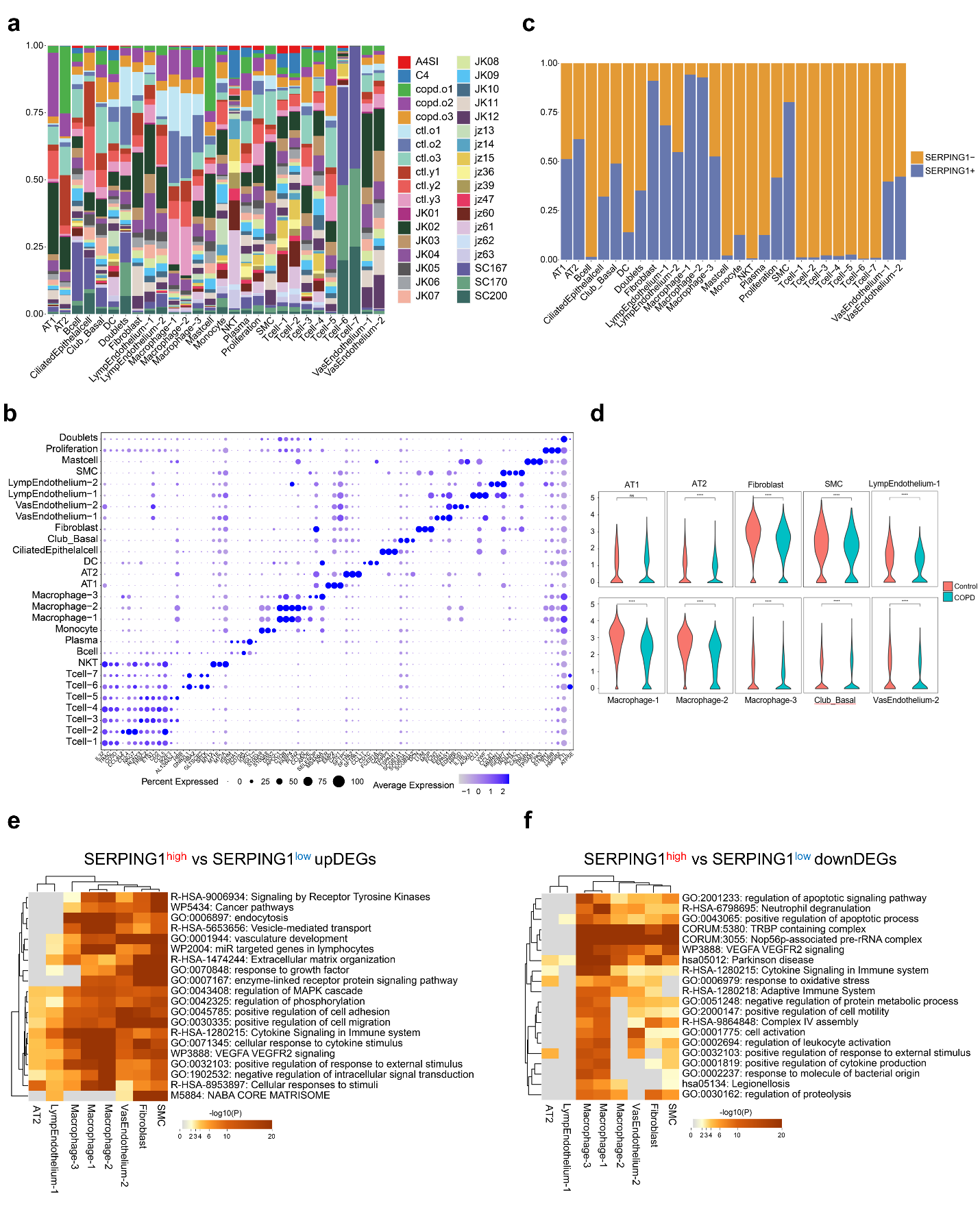


**Figure. S17.**

**a** The stacked bar chart illustrates cell subtype proportions across integrated human lung tissue single-cell RNA sequencing samples. **b** The bubble plot displays the top three marker genes for each cell subpopulation from the integrated human lung tissue single-cell RNA sequencing dataset, with marker specificity quantified by percentage and average expression. **c** Proportional distributions of *SERPING1^+^* versus *SERPING1^-^* cellular subpopulations across integrated human lung tissue scRNA-seq. **d** Differential expression profiles of *SERPING1* across nine annotated pulmonary cell subtypes: alveolar type I/II cells (AT1/AT2), fibroblasts, smooth muscle cells (SMC), lymphatic endothelium-1/-2 (LympEndothelium-1/-2), macrophage clusters 1-3 (Macrophage-1/-2/-3), club/basal cells (Club_Basal), and vascular endothelium-2 (VasEndothelium-2), comparing NC and COPD cohorts. Statistical significance between groups was assessed using two-tailed Student’s t-tests. Metascape Enrichment of up-regulated genes (**e**) and down-regulated genes (**f**) (*FDR < 0.05, log2FC > 0.2*) in *SERPING1^high^* versus *SERPING1^low^* subpopulations across seven pulmonary cell types: AT2, LympEndothelium-1, Macrophage-1/-2/-3, VasEndothelium-2, fibroblasts, and SMC.


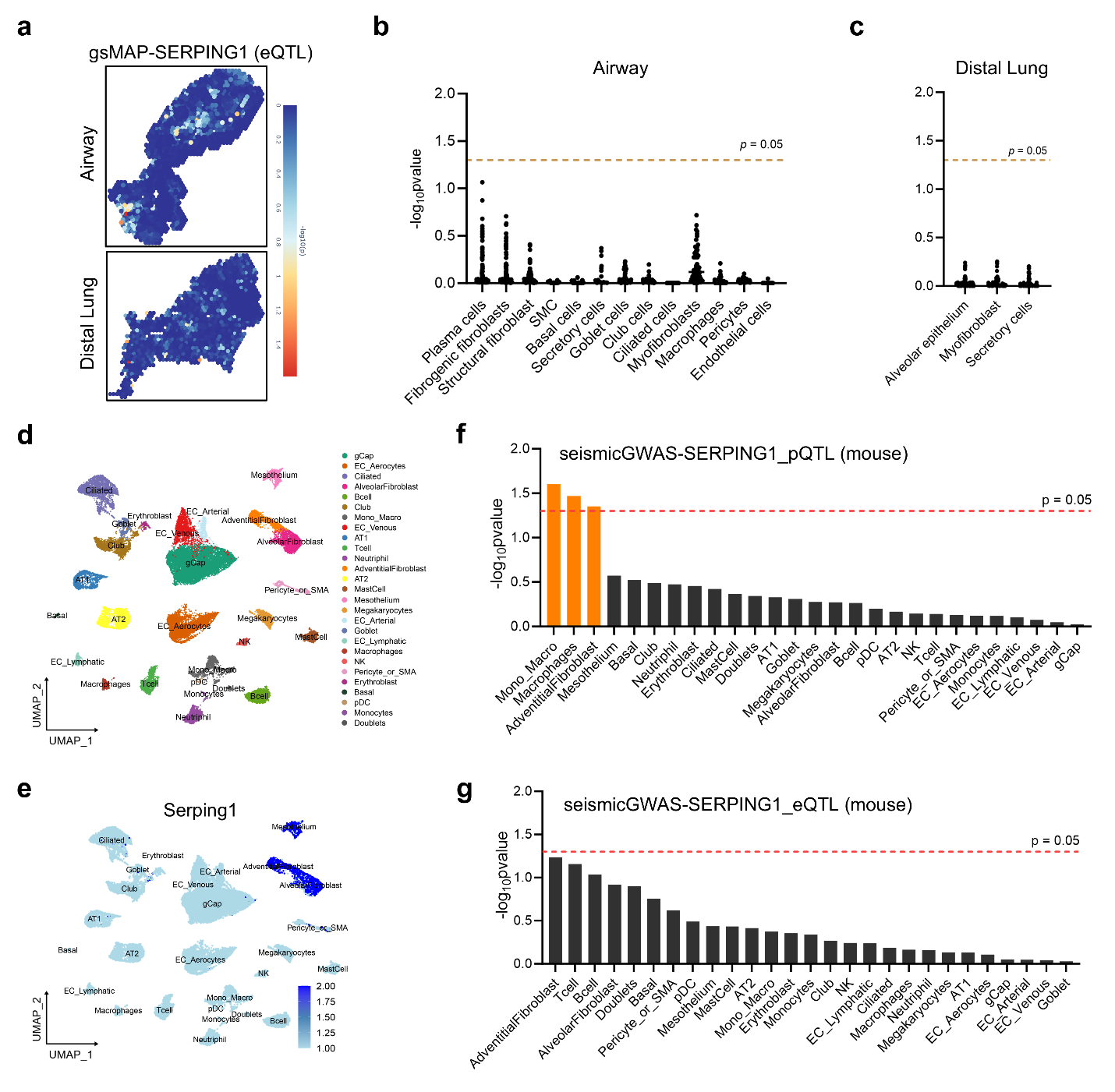


**Figure. S18.**

**a** Spatial transcriptomics of human airway/distal lung tissues from STOmics DB: H&E-stained sections with capture spots color-coded by annotated cell type and *SERPING1* expression overlays, and gsMAP-based multi-omic integration of *SERPING1* eQTL data (eQTLGen) with spatial transcriptomics (**b-c**); color gradient indicates -log₁₀(*p*-value) significance levels per vertical scale bar (blue: low; red: high). UMAP plots showing cell subpopulations (**d**) and *SERPING1* expression (**e**) distribution in mouse lung tissue from single-cell RNA sequencing data (GSE168299 dataset).​ Integration of *SERPING1* pQTL from Decode (**f**) and eQTL from eQTLGen (**g**) GWAS data with​mouse lung tissue scRNA-seq profiles using the ​seismicGWAS framework. Red dashed line indicated Nominal significance threshold (*p* = 0.05); ​ orange bars highlight cell subpopulations with significant seismicGWAS associations.


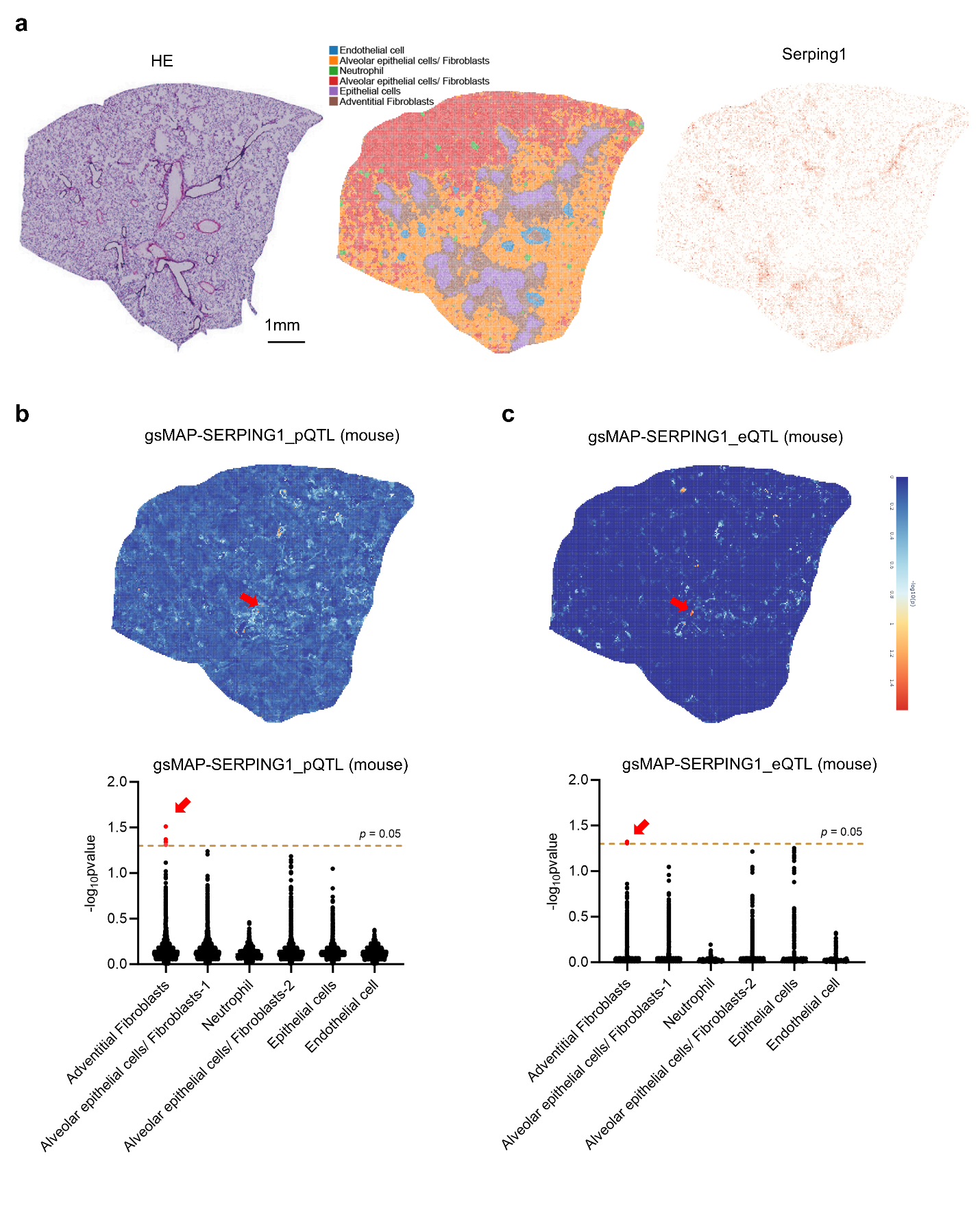


**Figure. S19.**

Spatial transcriptomics of mouse lung tissues from STOmics DB: H&E-stained sections with capture spots color-coded by annotated cell type and *SERPING1* expression overlays (**a**), and gsMAP-based multi-omic integration of *SERPING1* pQTL data (Decode) (**b**) and *SERPING1* eQTL data (eQTLGen) (**c**) with spatial transcriptomics; color gradient indicates -log₁₀(*p*-value) significance levels per vertical scale bar (blue: low; red: high).


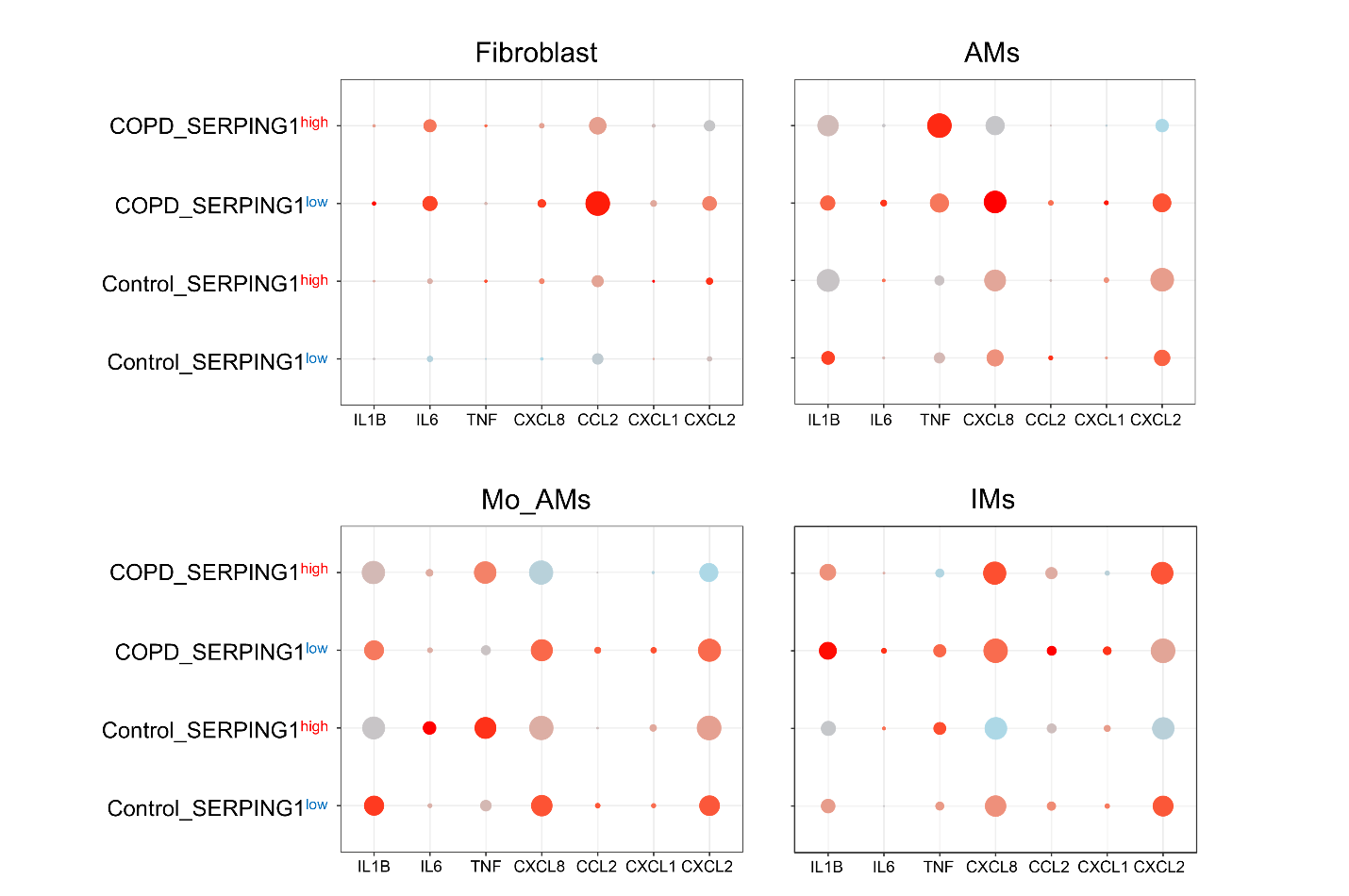


**Figure. S20.**

Expression distributions of *IL-1β*, *IL-6*, *TNF*, and chemokines (*CCL-2*, *CXCL-1/-2/-8*) across fibroblast and macrophage subpopulations in Control and COPD cohorts.


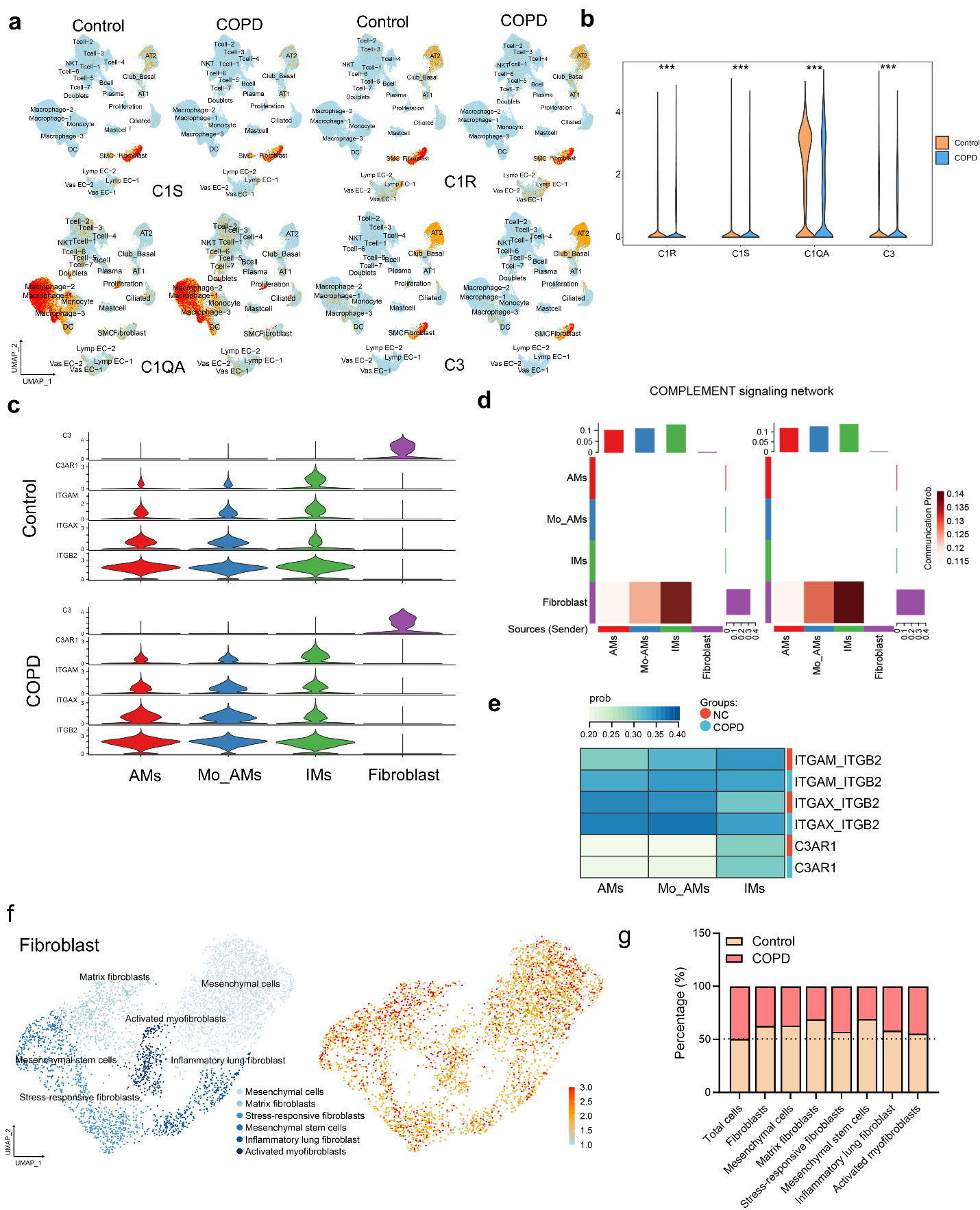


**Figure. S21.**

**a** UMAP projection of integrated human lung tissue scRNA-seq data displays expression. patterns of complement components *C1S*, *C1R*, *C1QA*, and *C3* across NC and COPD groups. **b** Violin plots compare expression levels of *C1S*, *C1R*, *C1QA*, and *C3* between NC and COPD groups. **c** Expression distributions of complement *C3* and its downstream receptors (*C3AR1*, *TIGIT*, *ITGAX*, *ITGB2*) in AMs, IMs, Mo_AMs and fibroblast subpopulations across NC and COPD cohorts. **d** Cell-cell communication network differences in complement signaling pathways between fibroblasts and IMs/AMs/Mo_AMs in Control versus COPD groups. Color intensity represents communication probability. **e** Heatmap showing COPD-associated changes in ligand-receptor interaction strengths (three complement pathways) from fibroblasts to IMs/AMs/Mo_AMs. Color gradient encodes communication probability. **f** UMAP visualization of fibroblast subclusters and *SERPING1* expression distribution. **g** Bar chart comparing proportions of fibroblast subclusters in Control and COPD groups.


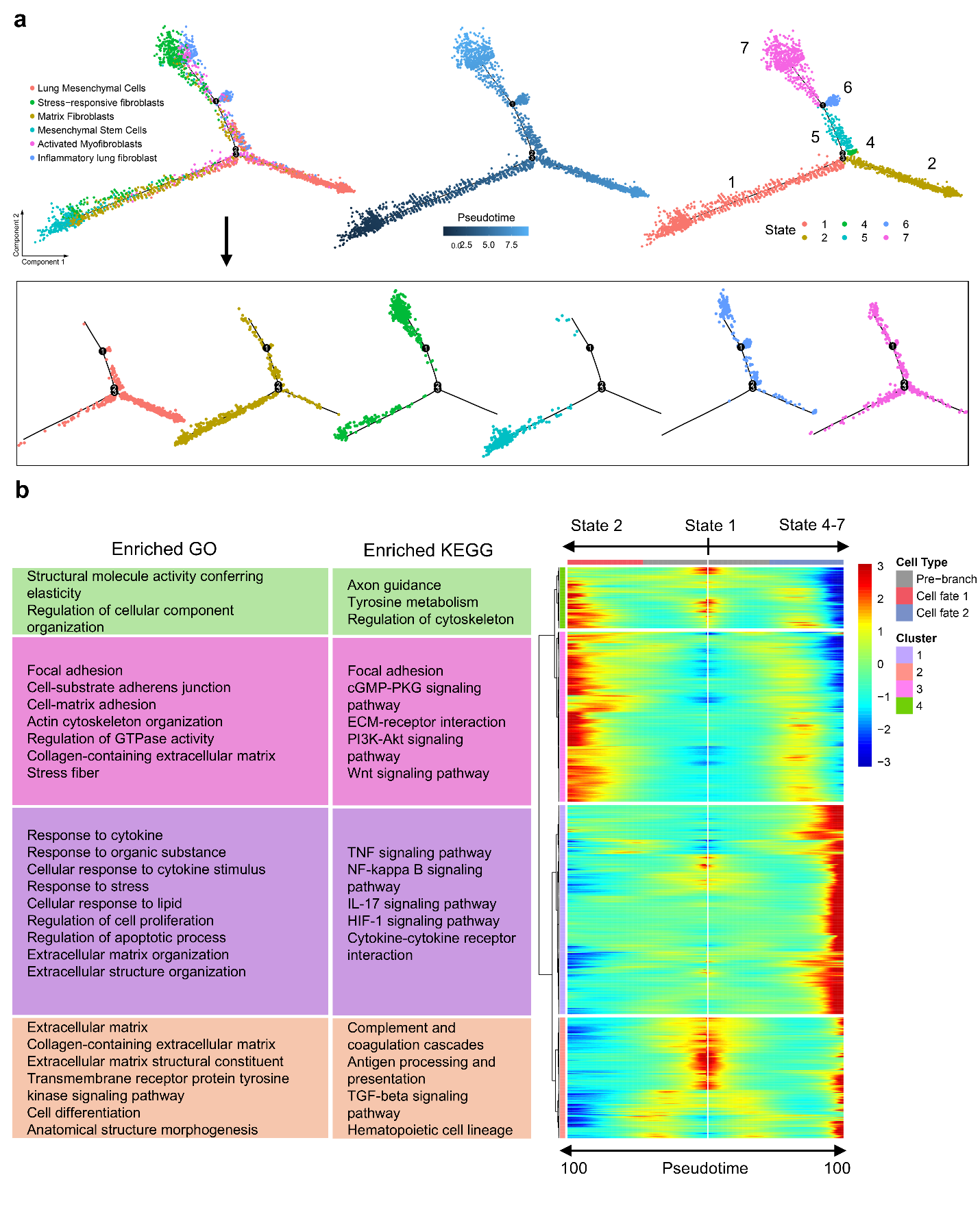


**Figure. S22.**

**a** Pseudotime trajectory analysis illustrating the developmental progression of fibroblasts, color-coded by six distinct states. **b** Heatmap displaying gene expression dynamics across pseudotime (0–100, bottom scale) for three complement signaling pathways, stratified by branch (State 1, State 2, States 4–7) and cell fate (Pre-Branch, Fate 1–2). Right panels: GO and KEGG enrichment results for marker genes of each cluster (Clusters 1–4).


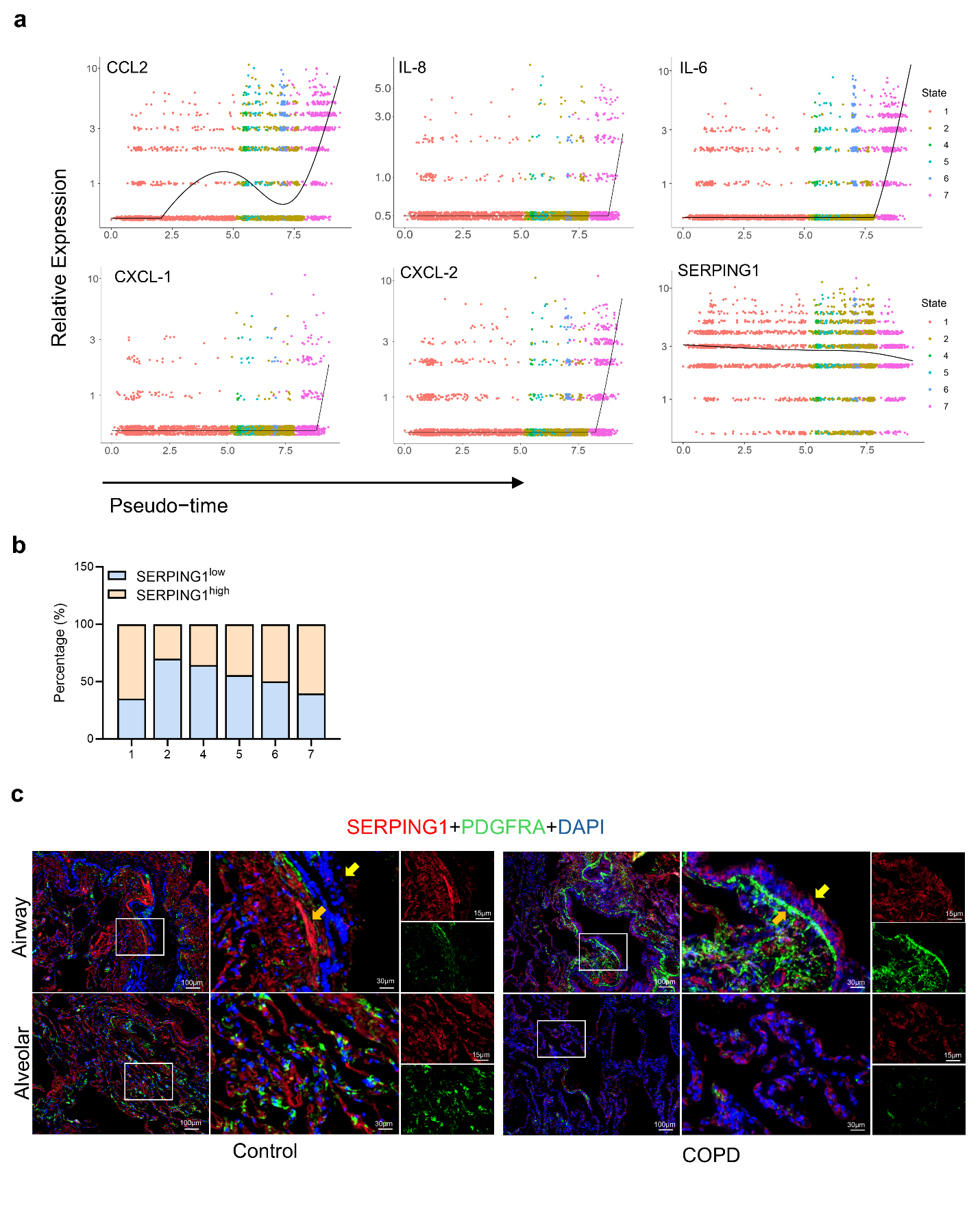


**Figure. S23.**

**a** Expression dynamics of *CCL-2*, *IL-8*, *IL-6*, *CXCL-1/-2*, and *SERPING1* across fibroblast pseudotime states. **b** Proportion of cells with high versus low *SERPING1* expression in distinct pseudotime states. **c** Multiplex immunofluorescence showing expression and colocalization of *SERPING1* (red) and PDGFRA (green) in lung tissue sections from non-COPD and COPD patients (n = 3). Nuclei counterstained with DAPI (blue).


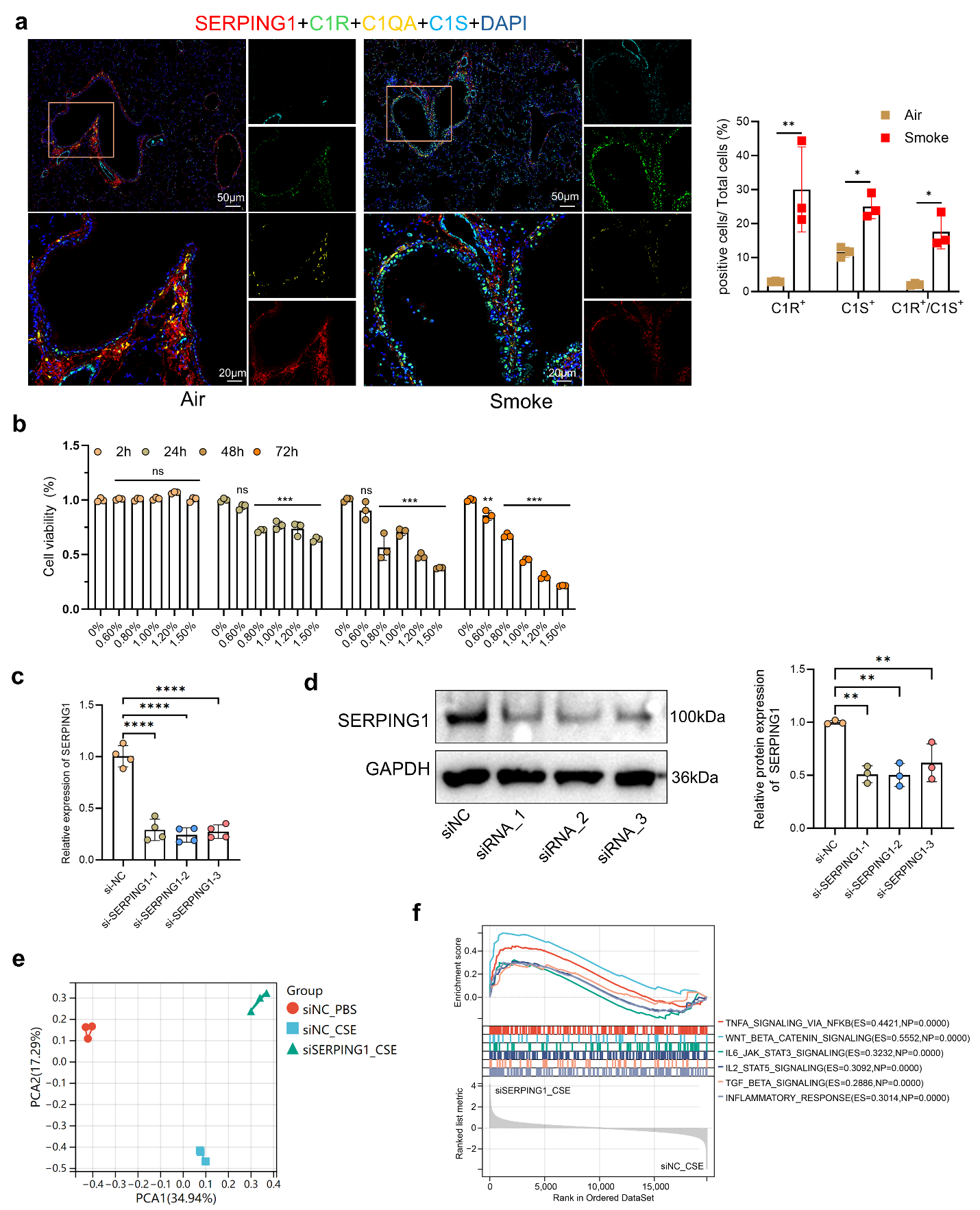


**Figure. S24.**

**a** Multiplex immunofluorescence​showing expression and colocalization of *SERPING1* (red), C1R (green), C1QA (yellow), and C1S (light blue) in lung tissue sections from air-exposed and cigarette smoke-exposed (CSE) mice. Bar plot: Quantification of C1s⁺, C1r⁺, and C1s⁺/C1r⁺ double-positive cells relative to total cells (n = 3). Nuclei counterstained with DAPI (blue). **b** Viability of HFL1 cells exposed to cigarette smoke extract (CSE) at varying concentrations and time points. (n=4). Efficiency validation of *SERPING1* siRNAs in HFL1 cells assessed by qRT-PCR (**c**) and Western blot (**d**) analysis. (n=4). **e** Principal component analysis (PCA)​of transcriptomes from *siNC_PBS*, *siNC_CSE*, and *siSERPING1_CSE* groups. **f** Gene Set Enrichment Analysis (GSEA) of Hallmark pathways for *siSERPING1_CSE* versus *siNC_CSE* comparisons. Data are expressed as mean ± SD. P values shown in charts determined by one-way ANOVA (**b, c, d**), Multiple paired t test (**a**, **b**).


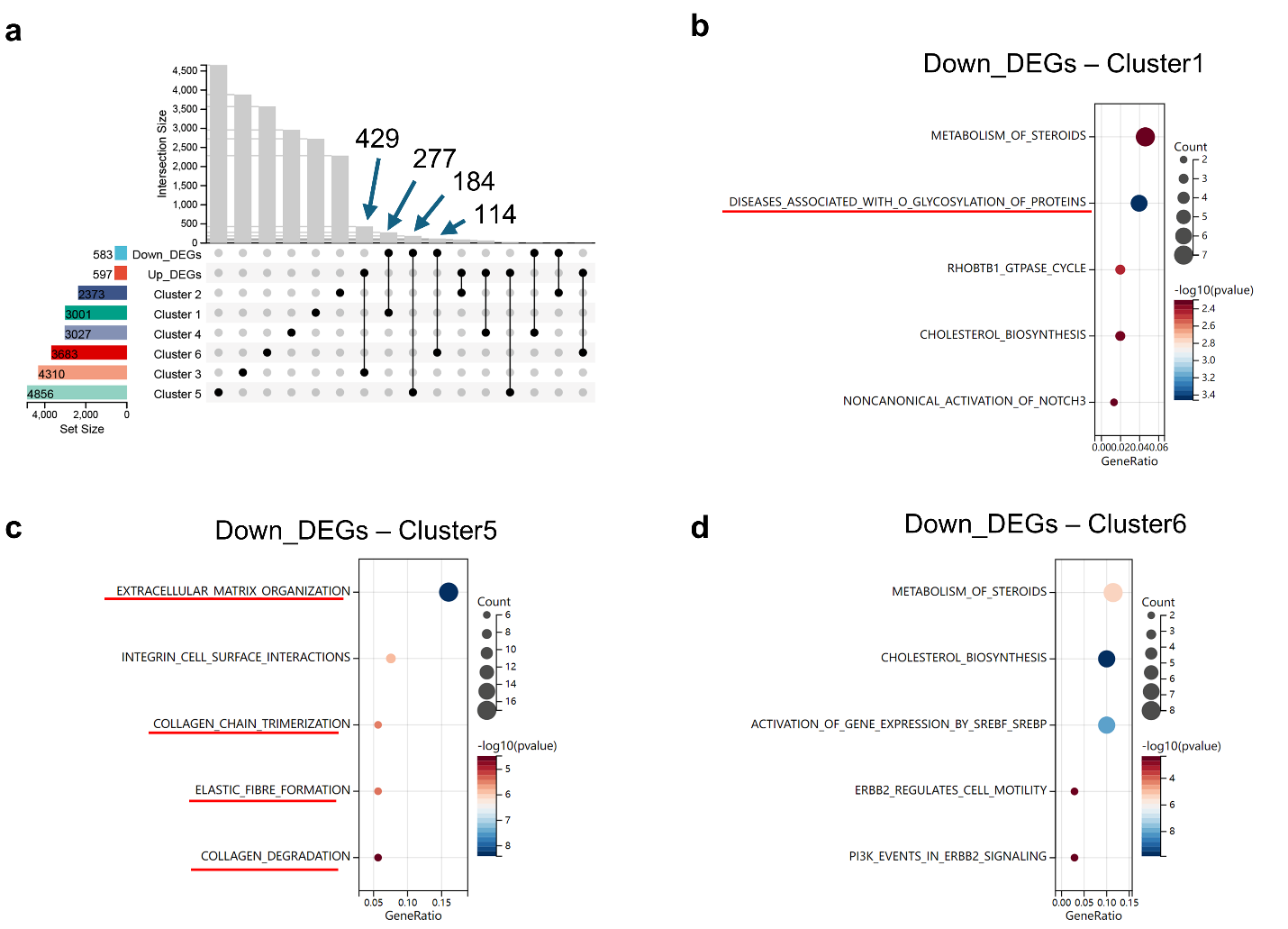


**Figure. S25.**

**a** Upset plot illustrating intersection relationships between Mfuzz clusters (1–6) and significantly up-/down-regulated gene sets from *siSERPING1*_CSE versus siNC_CE comparisons. Enrichment analysis results for intersection genes of Mfuzz cluster 1 with significantly downregulated genes (**b**), cluster 5 with downregulated genes (**c**), and cluster 6 with downregulated genes (**d**).


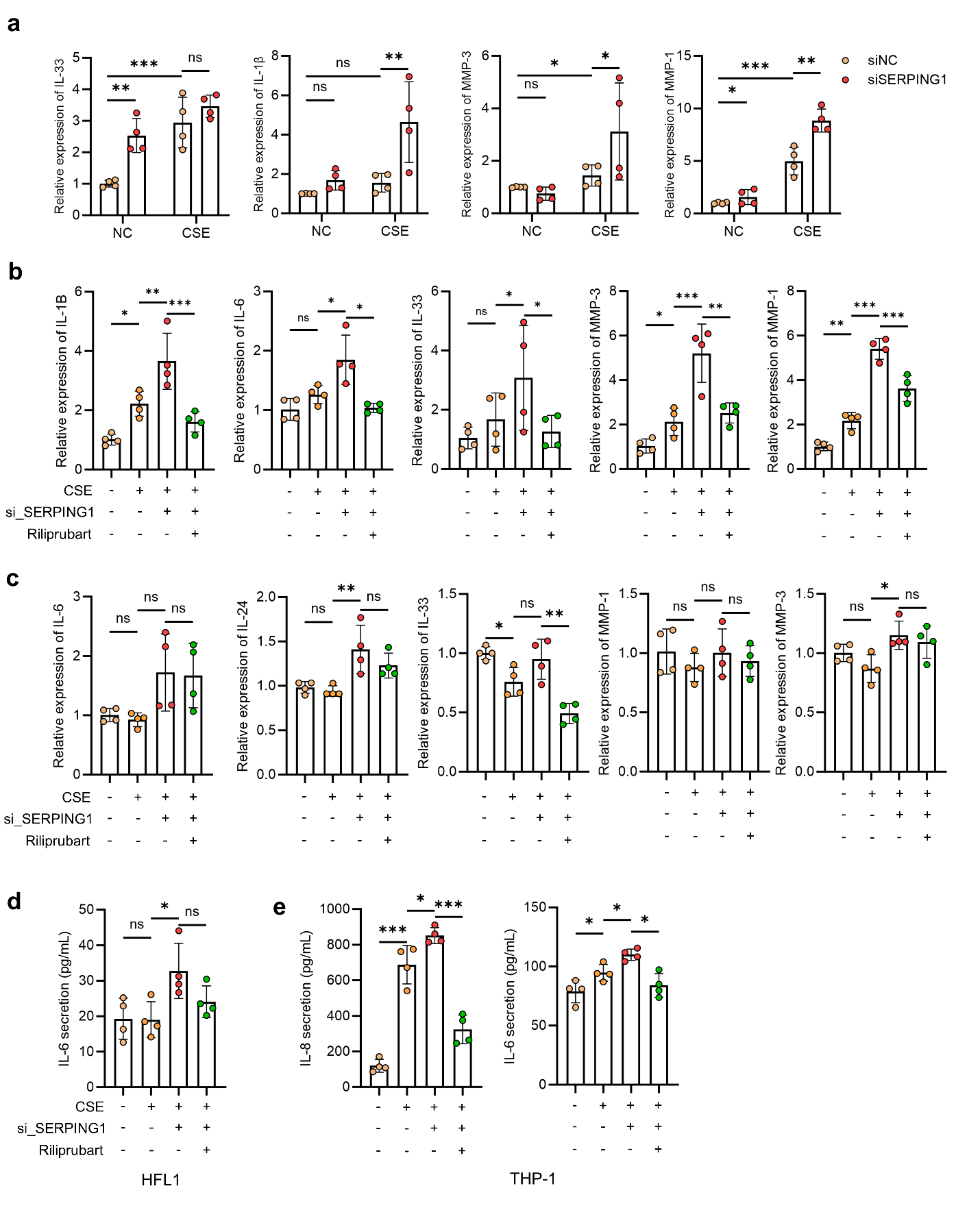


**Figure. S26.**

**a** Expression of *IL-33, IL-1β, MMP3*, and *MMP1* in *si-SERPING1*-transfected HFL1 cells with CSE stimulation, measured by qRT-PCR. (n=4). **b** Expression of *IL-33, IL-1β, IL-6, MMP3*, and *MMP1* in *si-SERPING1*-transfected HFL1 cells co-cultured with THP-1 macrophages under CSE stimulation with Rilzabrutinib treatment, assessed by qRT-PCR. (n=4). **c** Expression of *IL-6, IL-24, IL-33, MMP3,* and *MMP1* in THP-1 macrophages from the co-culture system, measured by qRT-PCR. (n=4). ELISA measured IL-6 secretion from co-cultured HFL cells (**d**) and IL-6/IL-8 secretion from THP-1 cells (**e**) in the co-culture system. (n=4). Data are expressed as mean ± SD. P values shown in charts determined by One-way ANOVA (**a, b, c, d, e**).


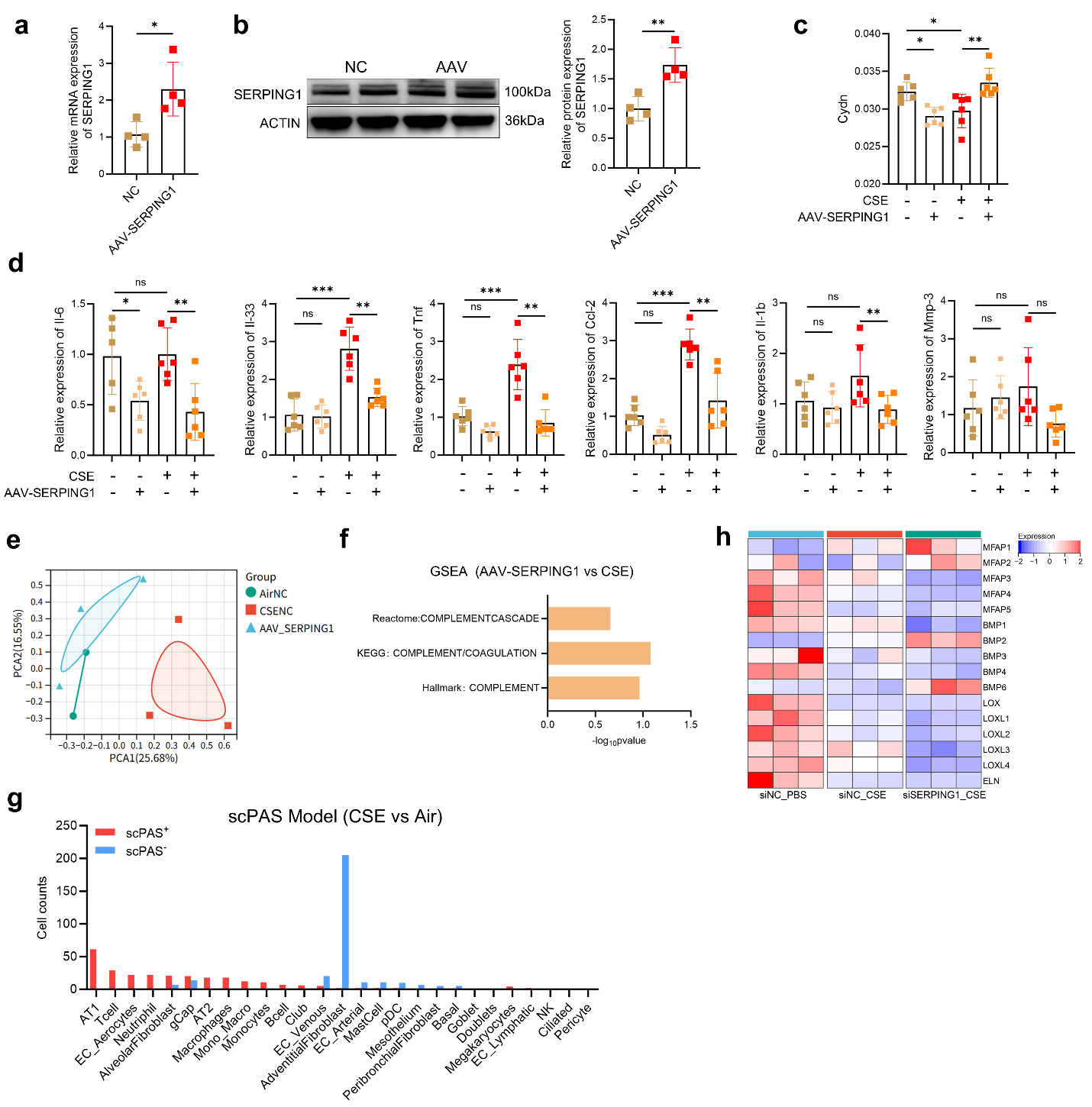


**Figure. S27.**

qRT-PCR (**a**) and Western blot (**b**) of *SERPING1* in AAV treatment mouse lungs (ACTIN reference). (n=4). **c** Pulmonary function evaluation of dynamic compliance (Cydn) in four experimental groups. (*n* = 6). **d** Expression levels of *Il-6, Il-33, Tnf, Ccl-2, Il-1β* and *Mmp3* mRNA in lung tissues of the four experimental groups. (*n* = 6). **e** PCA of lung tissue sequencing data across *Air-NC, CSE-NC* and *CSE-AAV-SERPING1* groups. (*n* = 3). **f** GSEA enrichment results of Reactome, KEGG and Hallmark gene sets in *CSE-NC* group versus *CSE-AAV-SERPING1* group.​ **h** Heatmap displaying expression levels of MFAP family, BMP family, LOX family, and ELN across three experimental conditions: *siNC-DMSO*, *siNC-CSE*, and *siSERPING1-CSE* (n=6). **g** Distribution of scPAS⁺ and scPAS⁻ cells across cellular subpopulations from mouse (GSE168299) in the scPAS results of NC-CSE versus NC-Air groups. Data are expressed as mean ± SD. P values shown in charts determined by two-tailed Student’s *t*-tests (**a**, **b**) and One-way ANOVA (**c**, **d**).


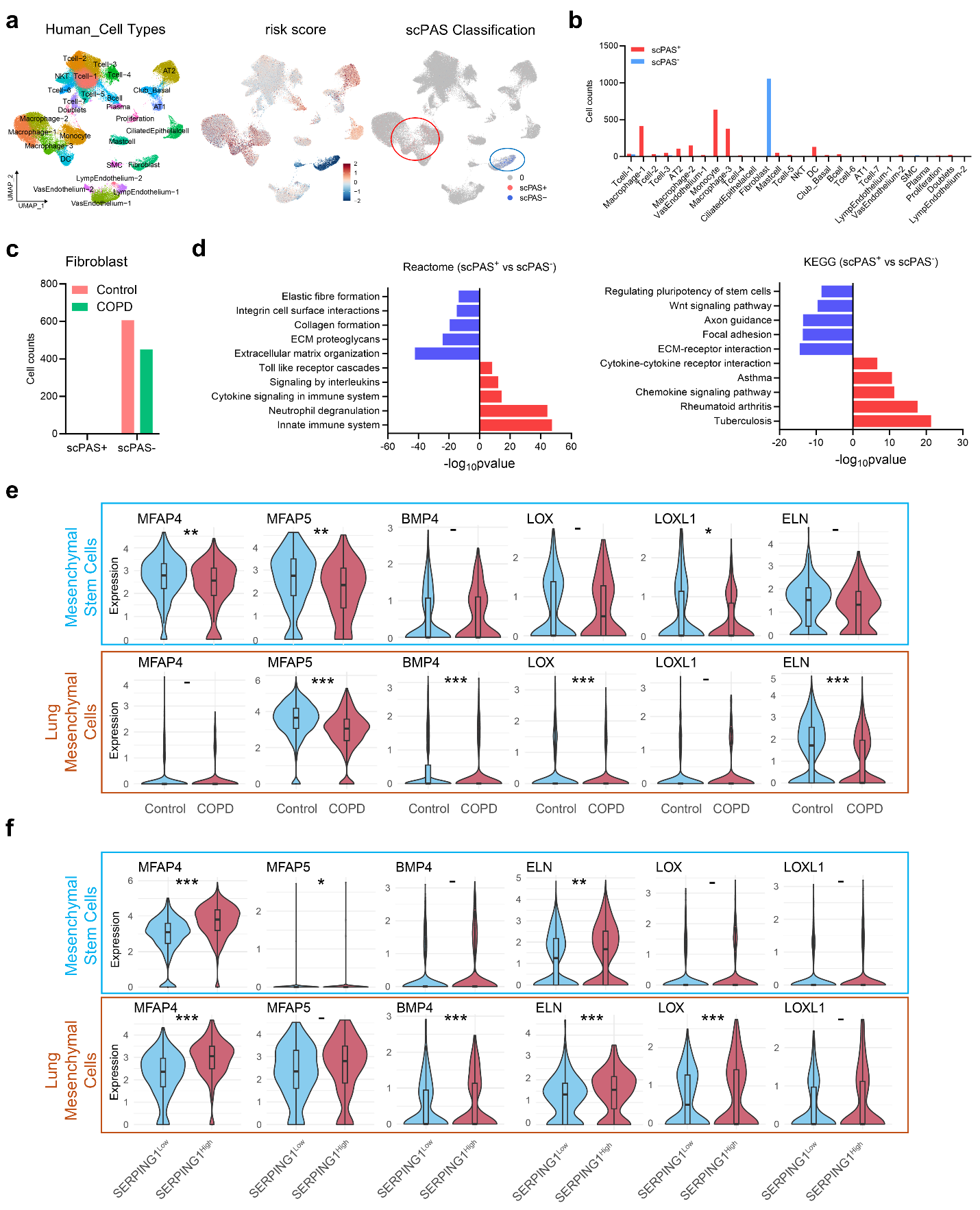


**Figure. S28.**

**a** UMAP visualization of GSE168299 (left), scPAS-derived risk scores (middle) and scPAS-selected cells (right) from integrated human lung tissue single-cell RNA sequencing data, comparing AAV-mediated *SERPING1* gene therapy-treated versus control cigarette smoke-exposed mice. Colors reflect risk score gradients (red: high; blue: low). **b** Distribution of scPAS⁺ and scPAS⁻ cells across cellular subpopulations from human lung tissue single-cell RNA sequencing data in the scPAS results of Smoke-exposed vs AAV-treated groups. **c** Quantity changes of scPAS-negative and scPAS-positive Fibroblasts in Smoke-exposed vs AAV-treated groups. **d** Reactome (left) and KEGG (right) enrichment of DEGs (*FDR* < 0.05, *FC* ≥0.25) in scPAS-positive vs. negative cells; bar length = *-log₁₀(FDR).* **e** Expression distributions of MFAP family, BMP family, LOX family, and ELN in COPD versus control groups for lung mesenchymal stem cells (MSCs) and lung mesenchymal cells (LMCs). **f** Expression distributions of MFAP family, BMP family, LOX family, and ELN in *SERPING1^high^* versus *SERPING1^low^* MSCs and LMCs.

# Reference

1 Bulik-Sullivan, B. K. *et al.* LD Score regression distinguishes confounding from polygenicity in genome-wide association studies. *Nat Genet* **47**, 291-295 (2015).

2 Duan, S., Zhang, W., Cox, N. J. & Dolan, M. E. FstSNP-HapMap3: a database of SNPs with high population differentiation for HapMap3. *Bioinformation* **3**, 139-141 (2008).

3 Zhu, Z. *et al.* Integration of summary data from GWAS and eQTL studies predicts complex trait gene targets. *Nat Genet* **48**, 481-487 (2016).

4 Sanderson, E., Spiller, W. & Bowden, J. Testing and correcting for weak and pleiotropic instruments in two-sample multivariable Mendelian randomization. *Stat Med* **40**, 5434-5452 (2021).

5 Xu, L., Borges, M. C., Hemani, G. & Lawlor, D. A. The role of glycaemic and lipid risk factors in mediating the effect of BMI on coronary heart disease: a two-step, two-sample Mendelian randomisation study. *Diabetologia* **60**, 2210-2220 (2017).

6 Wallace, C. A more accurate method for colocalisation analysis allowing for multiple causal variants. *PLoS Genet* **17**, e1009440 (2021).

7 Andreassen, O. A. *et al.* Improved detection of common variants associated with schizophrenia and bipolar disorder using pleiotropy-informed conditional false discovery rate. *PLoS Genet* **9**, e1003455 (2013).

8 Chang, Y. *et al.* COPD subtypes identified by network-based clustering of blood gene expression. *Genomics* **107**, 51-58 (2016).

9 Obeidat, M. *et al.* The Effect of Statins on Blood Gene Expression in COPD. *PLoS One* **10**, e0140022 (2015).

10 Kim, S. *et al.* Integrative phenotyping framework (iPF): integrative clustering of multiple omics data identifies novel lung disease subphenotypes. *BMC Genomics* **16**, 924 (2015).

11 Morrow, J. D. *et al.* Functional interactors of three genome-wide association study genes are differentially expressed in severe chronic obstructive pulmonary disease lung tissue. *Sci Rep* **7**, 44232 (2017).

12 Zhou, Y. *et al.* Metascape provides a biologist-oriented resource for the analysis of systems-level datasets. *Nat Commun* **10**, 1523 (2019).

13 Xu, S. *et al.* Using clusterProfiler to characterize multiomics data. *Nat Protoc* **19**, 3292-3320 (2024).

14 Kumar, L. & M, E. F. Mfuzz: a software package for soft clustering of microarray data. *Bioinformation* **2**, 5-7 (2007).

15 Watanabe, N. *et al.* Anomalous Epithelial Variations and Ectopic Inflammatory Response in Chronic Obstructive Pulmonary Disease. *Am J Respir Cell Mol Biol* **67**, 708-719 (2022).

16 Huang, Q. *et al.* Single-cell transcriptomics highlights immunological dysregulations of monocytes in the pathobiology of COPD. *Respir Res* **23**, 367 (2022).

17 Li, Y. *et al.* Heme oxygenase-1 determines the cell fate of ferroptotic death of alveolar macrophages in COPD. *Front Immunol* **14**, 1162087 (2023).

18 Li, X. *et al.* Single cell RNA sequencing identifies IGFBP5 and QKI as ciliated epithelial cell genes associated with severe COPD. *Respir Res* **22**, 100 (2021).

19 Sauler, M. *et al.* Characterization of the COPD alveolar niche using single-cell RNA sequencing. *Nat Commun* **13**, 494 (2022).

20 Hao, Y. *et al.* Dictionary learning for integrative, multimodal and scalable single-cell analysis. *Nat Biotechnol* **42**, 293-304 (2024).

21 Yi, E. *et al.* Long Noncoding RNA Interleukin 6 Antisense RNA 1 Promotes Inflammatory Effects in Lung Macrophages via Exosomes Through the S100A9/TLR4 Pathway in Chronic Obstructive Pulmonary Disease Progression. *MedComm (2020)* **6**, e70204 (2025).

22 Yi, E. *et al.* Smad3-mediated lncRNA HSALR1 enhances the non-classic signalling pathway of TGF-beta1 in human bronchial fibroblasts by binding to HSP90AB1. *Clin Transl Med* **13**, e1292 (2023).

23 Yi, E. *et al.* Genetic screening of MMP1 as a potential pathogenic gene in chronic obstructive pulmonary disease. *Life Sci* **313**, 121214 (2023).

24 Wu, F. *et al.* Rationale and design of the Early Chronic Obstructive Pulmonary Disease (ECOPD) study in Guangdong, China: a prospective observational cohort study. *J Thorac Dis* **13**, 6924-6935 (2021).

25 Yi, E. *et al.* An integrated machine learning model of transcriptomic genes in multi-center chronic obstructive pulmonary disease reveals the causal role of TIMP4 in airway epithelial cell. *Respir Res* **26**, 158 (2025).

Data S1-S10. (separate file)

Data S1: eQTL, pQTL, and mQTL datasets used in this study

Data S2: GWAS datasets used in this study

Data S3: SMR-based gene prioritization for COPD using eQTL data (Integrated analysis of GWAS summary statistics with eQTLGen and CAGE databases)

Data S4: SMR-based gene prioritization for FEV_1_ using eQTL data (Integrated analysis of GWAS summary statistics with eQTLGen and CAGE databases)

Data S5: SMR-based gene prioritization for FEV_1_%predict using eQTL data (Integrated analysis of GWAS summary statistics with eQTLGen and CAGE databases)

Data S6: ​SMR-based gene prioritization for FEV_1_/FVC using eQTL data (Integrated analysis of GWAS summary statistics with eQTLGen and CAGE databases)

Data S7: SMR-based gene prioritization for COPD using pQTL data (Integrated analysis of GWAS summary statistics with eight pQTL databases)

Data S8: SMR-based gene prioritization for FEV_1_ using pQTL data (Integrated analysis of GWAS summary statistics with eight pQTL databases)

Data S9: SMR-based gene prioritization for FEV_1_%predcit using pQTL data (Integrated analysis of GWAS summary statistics with eight pQTL databases)

Data S10: SMR-based gene prioritization for FEV_1_/FVC using pQTL data (Integrated analysis of GWAS summary statistics with eight pQTL databases)

Data S11-S18. (separate file)

Data S11: Integration of TSMR and Bayesian colocalization analyses for SMR-prioritized COPD genes (Leveraging eQTL resources: eQTLGen and CAGE).

Data S12: Integration of TSMR and Bayesian colocalization analyses for SMR-prioritized FEV_1_ genes (Leveraging eQTL resources: eQTLGen and CAGE).

Data S13: Integration of TSMR and Bayesian colocalization analyses for SMR-prioritized FEV_1_ %predict genes (Leveraging eQTL resources: eQTLGen and CAGE).

Data S14: Integration of TSMR and Bayesian colocalization analyses for SMR-prioritized FEV_1_/FVC genes (Leveraging eQTL resources: eQTLGen and CAGE).

Data S15: Integration of TSMR and Bayesian colocalization analyses for SMR-prioritized COPD genes (Leveraging pQTL resources with eight pQTL datasets).

Data S16: Integration of TSMR and Bayesian colocalization analyses for SMR-prioritized FEV_1_ genes (Leveraging pQTL resources with eight pQTL datasets).

Data S17: Integration of TSMR and Bayesian colocalization analyses for SMR-prioritized FEV_1_%predict genes (Leveraging pQTL resources with eight pQTL datasets).

Data S18: Integration of TSMR and Bayesian colocalization analyses for SMR-prioritized FEV_1_/FVC genes (Leveraging pQTL resources with eight pQTL datasets).

Data S19-S22. (separate file)

Data S19: Integration of SMR, TSMR and Bayesian colocalization analyses for Tier1 selected genes (Leveraging mQTL resources with LBC_BSGS_meta datasets).

Data S20: MOLOC-based colocalization analysis identifies putative shared genetic variants among SERPING1 eQTLs, pQTLs, and COPD traits

Data S21: HyPrColoc-based colocalization analysis identifies putative shared genetic variants among SERPING1 eQTLs, pQTLs, and COPD traits

Data S22: Reverse MR analysis of the association between COPD and SERPING1 expression (eQTL) and protein Levels (pQTL)

Data S23-S36. (separate file)

Data S23: Clinical information of s​tudy population​ from the UKBiobank cohort.

Data S24: Logistic regression modeling was employed to assess the association of prioritized candidate genes with COPD and Impaired lung function.​

Data S25: Linear regression modeling was employed to assess the association of prioritized candidate genes with spirometric indices

Data S26: Dichotomized SERPING1 expression is associated with COPD risk and lung function trajectories

Data S27: TSMR reveals smoking-driven causal effects on SERPING1 eQTL/pQTL, COPD risk and spirometric impairment

Data S28: Mediation effects of SERPING1 in smoke mediated COPD risk and spirometric impairment analyzed by mediation MR

Data S29: Clinical information of s​tudy population​ from the ECOPD cohort.

Data S30: Prioritized SERPING1 associate with questionnaire scores, spirometry, CT imaging, and accelerated lung function decline

Data S31: Dichotomized SERPING1 expression stratifies questionnaire scores, spirometry, CT imaging, and accelerated lung function decline

Data S32: TSMR and SMR results for the effects of SERPING1 eQTL/pQTL on IPF and Asthma

Data S33: Logistic regression modeling was employed to assess the association of Complement genes with COPD and Impaired lung function from UKB cohort.

Data S34: Longitudinal associations of complement genes with COPD susceptibility, spirometric impairment and accelerated FEV₁ decline across two clinical visits

Data S35: Stratified regression analysis shows differences in complement expression levels between White and Asian populations across disease groups

Data S36: Integration of TSMR and MVMR analyses for C1QA, C2, C3 and SERPING1 pQTL with COPD risk, lung function and spirometric impairment

Data S37-S41. (separate file)

Data S37: SERPING1 expression heterogeneity across experimental groups and cell types in integrated human single-cell RNA sequencing data

Data S38: Complement familys expression in disease states.​

Data S39: Top 30 marker genes in distinct subpopulations of pulmonary fibroblasts

Data S40: DEGs between scPAS⁺ and scPAS⁻ cells in AAV-treated versus smoke-exposed groups.​

Data S41: Primers used in experiments.
