# Supplementary figures and images for: Multiomics Mendelian randomization identifies serpin family G member 1 as a chronic obstructive pulmonary disease modulator
Source: Signal Transduct Target Ther. 2026 Jan 21;11:34. doi: 10.1038/s41392-025-02547-7 (PMC12819415; doi:10.1038/s41392-025-02547-7)

Figure 5B

B

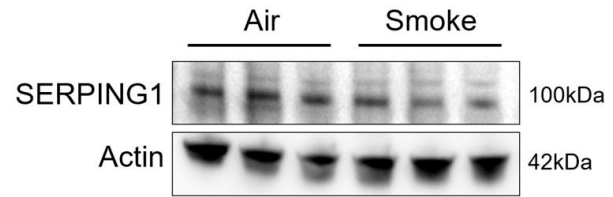

SERPING1

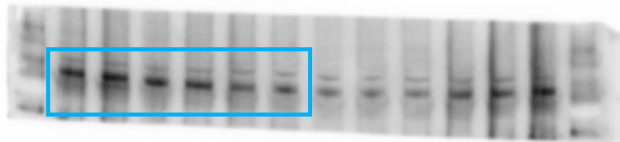

Actin

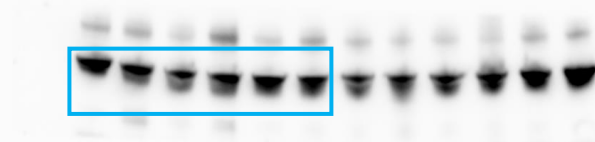

Figure S26B

D

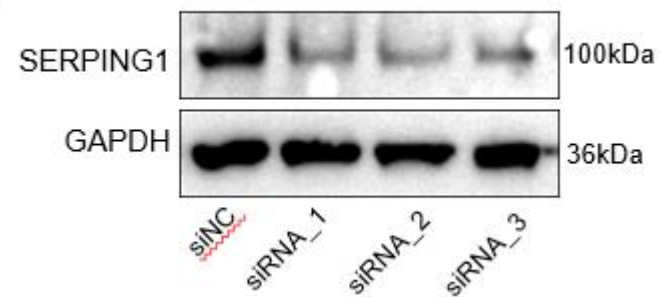

SERPING1

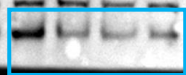

Actin

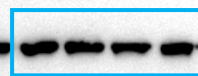

Figure SB

B

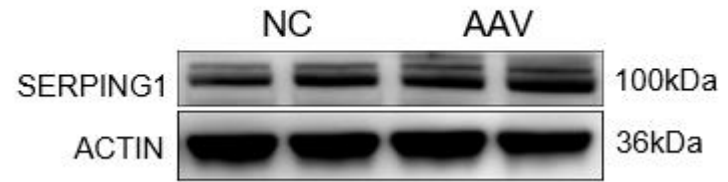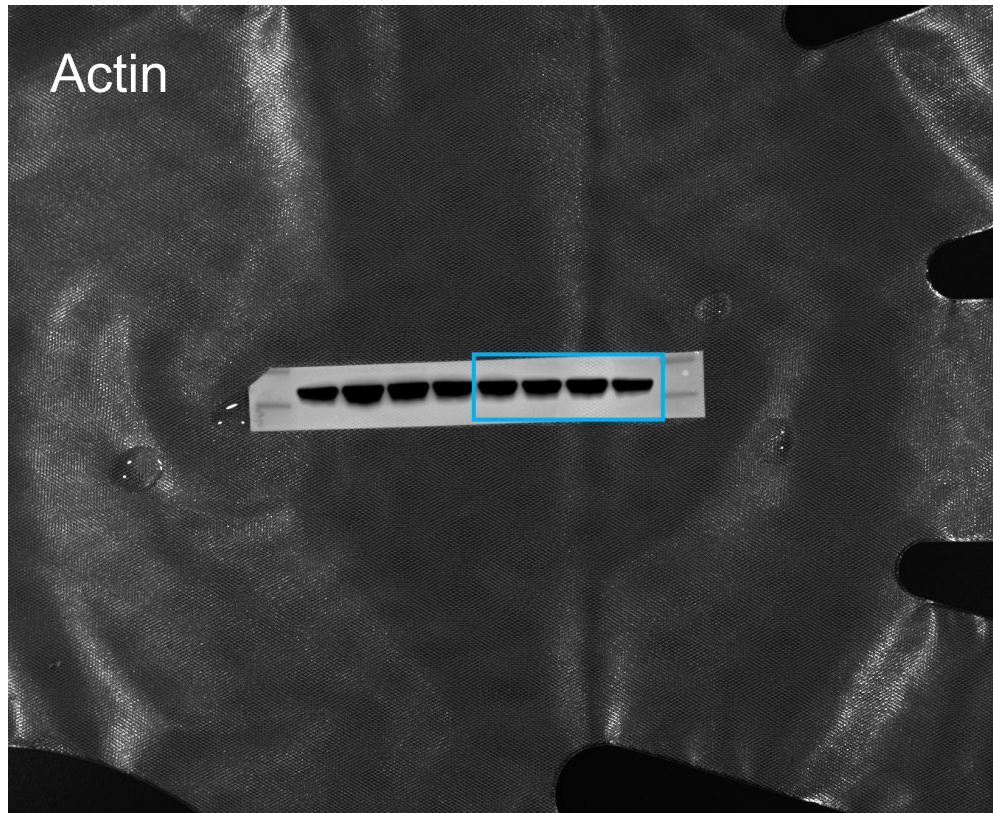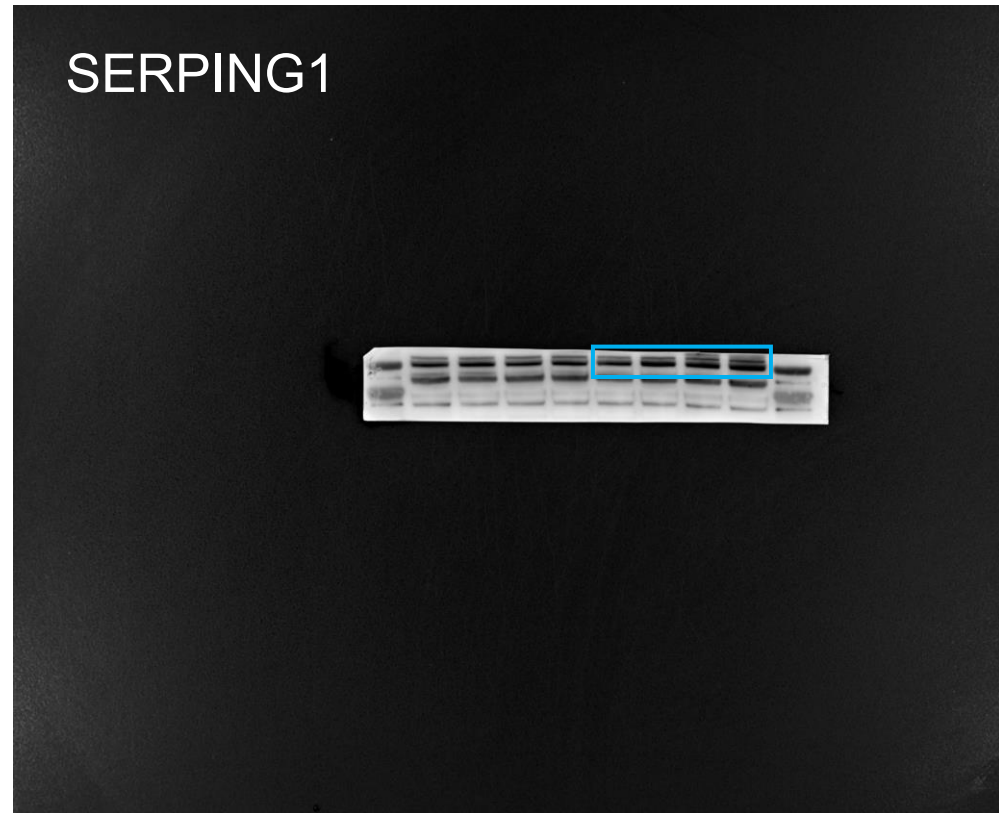

Supplement: Supplementary file 2 — Raw band for Western Blod [file 41392_2025_2547_MOESM2_ESM.pdf]
